# Supplementary material for: Cotton (Gossypium hirsutum) VIRMA as an N6-Methyladenosine RNA Methylation Regulator Participates in Controlling Chloroplast-Dependent and Independent Leaf Development
Source: Int J Mol Sci. 2022 Aug 31;23(17):9887. doi: 10.3390/ijms23179887 (PMC9456376; doi:10.3390/ijms23179887)
Supplement: Supplementary file 1 [file ijms-23-09887-s001.zip › Figures S1 and S6 Plants VIR alignment.pdf]

```
OsVIR           MGRPEPVVLFAQTILHSQLDEYVDEVLFSEPVVITACEFLEQNASPSTPNISLVGATSPP    60
AtVIR           MVRSEPCVLFAQTFVHPQLDEYVDEVIFAEPVIITACEFLEQNASSSSQAVSLVGATSPP    60
GhVIRA          MGRPEPCVLFSQTFVHTHLDEYVDEVLFAEPVVITACEFLEQNASSASQAVSLVGATSPP    60
GhVIRD          MGRPEPCVLFSQTFVHTHLDEYVDEVLFAEPVVITACEFLEQNASSASQAVSLVGATSPP    60
                * * ** ***:**::* :********:*:***:************ ::  :*********

OsVIR           SFALEVFVHCDGESRFRRLCQPFLYSHSSSNVLEVEFNGPYSSSEATAIAAFSMFVLFYQ    120
AtVIR           SFALEVFVRCEGESKFKRLCNPFLYTPSAPYPLEVE------------------------    96
GhVIRA          SFALEVFVQSEGETRFRRLCQPFLYSHSSSNVLEVE------------------------    96
GhVIRD          SFALEVFVQSEGETRFRRLCQPFLYSHSSSNVLEVE------------------------    96
                ********:.:**::*:***:****: *:   ****

OsVIR           AIVTNHLVLRGTYRSLTLVIYGNTAEDLGQFNIELDLDHSLANVVSSPSEGKLEDLPPAL    180
AtVIR           AVVTNHLVVRGSYRSLSLIVYGNIVKDLGQYNIILEGR-SVTDI-VSSTEGNLEDLPLVL    154
GhVIRA          AVVTNHLVVRGSYRSLSLVIYGNTAEDLGQFNIEFDDS-SLTNL-VGSADGKLEDLPLPL    154
GhVIRD          AVVTNHLVVRGSYRSLSLVIYGNTAEDLGQFNIEFDDS-SLTNL-VSSADGKLEDLPLPL    154
                *:******:**:****:*::*** .:****:** ::   *::::  . ::*:*****  *

OsVIR           HSSKFTFEESLSSLKPLSLQATELDLSIEVKKILLLALTMYQIPNVENLIPNLQSAVISA    240
AtVIR           HSVNRTIEECLSSLDIVSLPLAAVDLPVEVKRLLQLLLKIFDKLATNDVVNKFVDTVVSG    214
GhVIRA          RAFNRTFEESLSSLNVLSLPVVTLDLSVEVKQLLQQMLKILELPNLGHEVHKVVHTLALA    214
GhVIRD          CAFNRTFEESL-SLNVLSLPVVTLDLSVEVKQLLQQMLKILELPNLGHEVHEVVHTLALA    213
                 : : *:**.* **. :**  . :** :***::*   *.: :     . : :.  ::  .

OsVIR           VLKYMPASTNCMSRNWNRDPANCFAE-DNVDSQGTSNTLLMEASNELFDIWKN-------    292
AtVIR           VSSYVTDNVDFFLKNK-----NCSAVTSSLD-SGLFHDIVDRVKEDILDLN------EIQ    262
GhVIRA          AASFVTFDLESNAINQ-----KHLTSGRNKDFKELNHGIS-EARKELLELYETLQRKSTN    268
GhVIRD          AASFVTFDLDSNAINQ-----KHLTSGRNKDFKELNHGIS-EAKKELLELYETLQRKSMN    267
                . .::  . :    *      :  :   . * .   : :  .. ::::::

OsVIR           ------------------------VNSIVDNITFDDNGLAFRLEELPTTKHLF-------    321
AtVIR           ESDVALGLFSFLESETYLATSQQLVVMLSPYIQFERDSLCTVLPKLSKGKATLLGLSLAF    322
GhVIRA          KSSESLTECIFMESDADLASSKQLVEMLSPCFHFNRSSSNFGHGQLPESKNVILGLNVAL    328
GhVIRD          ESSESLTECIFMESDADLASSKQLVEMLSPCFHFNRSSSSFGHGQLPESNNVILGLNVAL    327
                                        *  :   : *: ..      :*   :  :

OsVIR           ---TLFDSCFPYYRNCSLLDLECPFQ----------------------------------    344
AtVIR           LLCSGREGCLQFVNSGGMDQLVYLFGHDGQNSTTITLLLLGVVEQATRHSVGCEGFLGWW    382
GhVIRA          FLCSTKESCFHFVNCGGMDQLAYLFDHQTQNSITITLLLLGVIEQATRHSVGCEGFLGWW    388
GhVIRD          FLCSAKESCFHFVNCGGMDQLAYLFDHQMQNSITITLLLLGVIEQATRHSVGCEGFLGWW    387
                   :  :.*: : .  .: :*   *

OsVIR           -------------------------PLVDL--------------------QSAVVNIVSN    359
AtVIR           PREDGSIPSGKSEGYCLLLKLLMQKPCHEIASLAIYILRRLRIYEVISRYEFAVLSALEG    442
GhVIRA          PREDENIPSGTSDGYSYLLKLLLQKPRHDVASLATYILHRLRFYEVISRYESEILSILGG    448
GhVIRD          PREDENIPSGTSDGYSYLLKLLLQKPRHDVASLATYILHRLRFYEVISRYESEILSILGG    447
                                         *  ::                    :  ::. : .

OsVIR           LPS-EELSSDGVNFLSSASIELAELLKMINMCVPIEDPSPVLTARRICKFGHLEGLLSYN    418
AtVIR           LSNSHGAATHNLNMLSDAKSQLQKLQNLMKSLGSVEDPSPSAYAERSLVSDHSEGWLSYK    502
GhVIRA          LSATTKGTNAASNKLRGVGSLLKKLLHLVISHGPIEDPSPVAHASRYFILGQTDGLVSYN    508
GhVIRD          LSATTKGTNVASNKLRGVGSLLKKLLHLVISHGPIEDPSPVAHASRYFILGQTDGLVSHK    507
                *      :.   * * ..   * :* :::     :*****   * *    .: :* :*::

OsVIR           LTIGLITSSKYSFLQFDADPYMLSLIQEDASGNLERRELVGPTGHTLPMRAPATHAYSST    478
AtVIR           ATSKLTSSWTCPFYSSGIDSHILALLKERGFLPLSAALLSMPELH---------------    547
GhVIRA          ATSGLIASSNCCFSDWEIDLHLLALLKDRGFLPLSAALLSTTVLH---------------    553
GhVIRD          ATSGLIASSNCCFSDWEIDLHLLALLKDRGFLPLSAALLSTTILH---------------    552
                 *  * :* .  * .   * ::*:*::: .   *.   *     *

OsVIR           SAPTTLLCPCLPESSSGCGILPAPPSFNPCRCDKPSSDVAHGIPAIPHYCLLSADGLSFL    538
AtVIR           ---------------SKV-----------GDIMDVFTDIAMFIGNIILSFMFSRTGLSFL    581
GhVIRA          ---------------SEA-----------ADVVDTSLEIVSSIGSIILSLLFCRSGLVFL    587
GhVIRD          ---------------SEA-----------ADVVDTSLVIVSSIGSIILSLLFCRSGLVFL    586
                               *                .    :.   *  *    ::.  ** **

OsVIR           LGQPEATELILLSLQDGEDMSKTECMTLRQAFVLLSKGFFCRPQEVAMITELHLKVEHSL    598
AtVIR           LHHPELTATIIQSLKGSVDLNKEECVPLHYASILISKGFTCSLLEIGINLEMHLRVVSAV    641
GhVIRA          LHQPDLTATLIHALKGADAMNKEECVPLRYASVLISKGFTCNPQEVGIIVETHLRVVNAI    647
```

```
GhVIRD      LHQPDLTATLIHALKGADAMNKEECVPLRYASVLISKGFTCSPQEVGIIVETHLRVVNAI      646
            * :*: *  :: :*:..  :.* **: *: * :*:**** *   *:.:  * **:*  ::

OsVIR       FA------------YNLLNLKVSTLLSHID-------DLNYLAVSVLLSSLSSYNDLDSV      639
AtVIR       DRLLKSIQQTEEFLWILWELRDVS-RSDCGREALLTLGVFPEALAVLIEALHSAKDMEPA      700
GhVIRA      DRLLSATPQSEEFLWVLWELCGLA-RSDCGRQALLAMSFFSEVLSVLIEALHSVKESEPV      706
GhVIRD      DCLLSATPQSEEFLWVLWELCGLA-RSDCGRQALLAMSFFSEVLSVLIEALHSVKESEPV      705
                          : * :*   :  *. .       ..   .::**:.:* * :: : .

OsVIR       TNKNGGSPLGHAIFHSTAEILEVLVADSTASSLKSWIGFAIDLHKALHSSSPG-SNRKDA      698
AtVIR       VENSGISPLNLAICHSAAEIFEVIVSDSTASCLHAWIEHAPVLHKALHTLSPGGSNRKDA      760
GhVIRA      IKNSGASPLNLAILHSAAEIVEVIVTDSTATSLSSWIGHAMELHKALHSSSPG-SNRKDA      765
GhVIRD      IKNSGASPLNLAILHSAAEIVEVIVTDSTATSLSSWIGHAMELHKALHSSSPG-SNRKDA      764
             ::.* ***. ** **:***.**:*:****:.* :** .*  ******: *** ******

OsVIR       PTRLLEWIDAGVVYKRNGAVGLLRYSAILASGGDAHLSSGNVLVSDSMDV-ENVVADPNN      757
AtVIR       PSRLLKWIDAGVVYHKHGVGGLLRYAAVLASGGDAQLSSSSILALDLTPA-ENGAGESTN      819
GhVIRA      PTRLLEWIDAGLVYHKNGAVGLLRYAAVLASGGDAHLTSTNILVSDLTDVVDNIVGESSN      825
GhVIRD      PTRLLEWIDAGLVYHKNGAVGLLRYAAVLASGGDAHLTSTNILVSDLTDVVDNIVGESSN      824
            *:***:*****:**:::*. *****:*:*******:*:* .:*. *   . :* ..: .*

OsVIR       TDGQVIDNLLGKLVADKYFDGVALCSTSVVQLTTAFRILAFISEEKAVASSLFEEGAINV      817
AtVIR       VSEMNVLDNLGKVIFEKSFEGVNLSDSSISQLTTALRILALISDNSTVAAALYDEGAVTV      879
GhVIRA      ASDINVMENLGSIISMKSFEGVNLRDSSIAQLTTAFRILAFISENPTVAAALYDEGAITV      885
GhVIRD      ASDINVMENLGSIISMKSFEGVNLRDSSIAQLTTAFRILAFISENPTVAAALYDEGAITV      884
            ..   : : **.::  * *:** * .:*: *****:****:**:: :**::*::***:.*

OsVIR       IYVVLMNCKSMLERLSNSYDYLVDEGAELSSTTELLLDRTHEQTLVDLMIPSLVLLINLL      877
AtVIR       VYAILVNCSFMFERSSNIYDYLVDDDHGCSSISDFLSERNREQSLVDLLIPSLVLLISVL      939
GhVIRA      IYVVLVNCSYMLERSSNSYDYLVDEGTECNSTSDLLLERNREQCLVDLLIPSLVLLITLL      945
GhVIRD      IYVVLVNCSYMLERSSNSYDYLVDEGTECNSTSDLLLERNREQCLVDLLIPSLVLLITLL      944
            :*.:*:**. *:** ** ******:.   .* :::* :*.:** ****:********.:*

OsVIR       HILNETKEQYRNKKLLTALLQLHREVSPRLAACAADLSFMFPSFAVSFGVVCHLVTSAIA      937
AtVIR       QRLQGTKEQYRNTKLMKALLRLHREVSPKLAACAADLSSHYPDSALGFGAVCHLIVSALV      999
GhVIRA      QRLQEAKEQHKNTKLMTALLRLHREVSPKLAACAADLSSPYPDSALGFEAVCHLSVSALA      1005
GhVIRD      QRLQEAKEQHKNTKLMNALLRLHREVSPKLAACAADLSSPYPDSALGFEAVCHLSVSALA      1004
            : *: :***::*.**:.***:*******:*********  :*.  *:.* .**** .**:.

OsVIR       CWPLYNWAPGLFHCLLENVEATNAAVPLGPKDACSLLCLLGDLFPDEGIWLWKVEVPSLT      997
AtVIR       CWPVYGWIPGLFHTLLSGVQTS-SVPALGPKETCSFLCILSDILPEEGVWFWKSGMPLLS      1058
GhVIRA      YWPVYGWSPGLFHTILASVQTT-SSLALGPKETCSLLCLLNDLFPEESIWRWKNGMPLLS      1064
GhVIRD      YWPVYGWSPGLFHTILASVQTT-SSLALGPKETCSLLCLLNDLFPEESIWHWKNGMPLLS      1063
             **:*.* ***** :* .*::: :   ****::**:**:*.*::*:*.:* **  :* *:

OsVIR       AIRSLSTGTVLGCQVEKHMNWYLHPEHVSILLVRLMPQLDRLACVIDNFATSALTVIQDM      1057
AtVIR       GLRKLAVGTLMGPQKEKQINWYLEPGPLEKLINHLTPNLDKIAKIIQHHAVSALVVIQDM      1118
GhVIRA      ALRSLAIGTLLGPHKERQVDWYLECGHLEKLFNQLTPHLDRIAQIIQHYAISALVVIQDM      1124
GhVIRD      ALRSLAIGTLLGPHKERQVDWYLECGHLEKLFNQLTPHLDRIAQIIQHYAISALVVIQDM      1123
            .:*.*: **::* : *::::***.   :. *: :* *:**::* :*::.* ***.*****

OsVIR       LRIFIVRIASEKIECAVVLLRPIFIWLNNKV-DETSLSEREIFKVHQLLQFIAKLSEHPN      1116
AtVIR       LRVFIVRIACQRVEHASILLRPIFSSIRDGILDQSSTRDTEAYMVYRYLNFLASLLEHPH      1178
GhVIRA      LRVFIIRIACQKAEQASKLLRPILSWIHDHSSDLSSLSDTEAYKVYRCLDFLTSLLEHPY      1184
GhVIRD      LRVFIIRIACQKAEQASKLLRPILSWIHDHTSDLSSLSDTEAYKVYRCLDFLTSLLEHPY      1183
            **:**:***.:: * *  *****:  :.:   * :*  : * : *:: *:*::.* ***

OsVIR       GKALLCKMGVARILRKLLQECSSMCYMEDNMISDK-GVYSNDLLMLRWKIPLLRSIASIF      1175
AtVIR       AKGLLLEEGIVQLLVEVLERCYDATYPSENRVLEY--GIVSASSVIQWCIPAFRSISLLC      1236
GhVIRA      AKVLLVGEGFPQILTRVLESCFDATDSDGRQASDCRDSAKYGVALISLCIPVFKSISLLC      1244
GhVIRD      AKVLLVGEGFPQILTRVLESCFDATDSDGRQASDCRDFAKYGFALISLCIPVFKSISLLC      1243
            .* **   *. ::* .:*: * .    . .   :          ::   ** ::**: :

OsVIR       STRPSSKEPTTVEELWNENACVEECSSIMYHLLMLCQVLPVGRDMFACSLAFKEVASSYS      1235
AtVIR       DSQVPLLCFQK--KELLASLSAKDCALIFPFVLKFCQVLPVGNELLSCLGAFKDLSSCGE      1294
GhVIRA      SSRTFSQYDERHEMHKFDSLSPKDCSIFINQLLKFCQVLPVGKELVSCLTAFRDMGSCTE      1304
GhVIRD      SSLTFPQYDERHEMHKFDSLSPKDCSIFINQLLKFCQVLPVGKELVSCLTAFRDMGSCTE      1303
            .:                . . ::*: ::  :* :*******.::.:*  **:::.*. .
```

```
OsVIR        -CRGAVTSIFSQIQTSNKDESQKSESETCH------DTSKVDNWCGFFPLLKCWKRLLQY        1288
AtVIR        GQDGLVSLLFHLFS-GTEES---VSERWCD---TNSLSLDQLDMKKNPPFLSCWIKLLNS        1347
GhVIRA       GCNALLSALLNSSS-STHDE---LESERGNEKNVNFHFLNESEWRKSPPLLCCWIKLLKS        1360
GhVIRD       GRNALLSALLYSSS-STHDE---LESERGNEKNVNFHFLNESEWRKSPPLLCCWIKLLKS        1359
                . :: ::   . ...:.    ...   .          .   :     *:* ** :**:

OsVIR        ICANR-PTDYLVEIVYALTLGAIALSQSGGQNLE--GTIILRRLFGHPSVPSSSEASDEVT        1345
AtVIR        INSKDGLSSLAMKAVNVLSVGSIRLCLDGKSLDSKKVAALKSLFGLPSEFSGTDTFREEN        1407
GhVIRA       IDSKDHLPSYTLEAANVLSLGTLGFCMGGNSLNMNSVVALKKFLFGLPDDTAGIGGFPEDN        1420
GhVIRD       IDSKDHLPPYTLEAANVLALGTLGFCMGGNSWNMNSVVALKKFLFGLPDDTTGIGGFPEDN        1419
             * :.        :: . .*::*:: :. .*:. :    .   *: *** *.   :.      * .

OsVIR        F-LLKTFQEKICQ---GFDNW-SPYVGKPLLHQVRSSVRLLCSIIENSGPFTDSVRMVLE        1400
AtVIR        IGLIEQMVTLLSSMTSGSDSS-ATAEMKPYLHEASQSLLS---LL----K----------        1449
GhVIRA       IKYIQEFSTLLSSRIDNDEDYQTSSDIHISMHQVSESVKSLLLLFQNLTA----------        1470
GhVIRD       IKYIQEFSTLLSSRIHNDEDYQTSSDIHISMHQVSESVKSLLLLFQNLTA----------        1469
             :  :: :   :..   . :.   :     :  :*:. .*:      ::

OsVIR        ESTIPVGVFHNIVMTSHLMPSIDFVSVNDDPALLFTNAWKAFGDFAE---PFGCQVSDFS        1457
AtVIR        ----DGNIDDIISCKGVF----------------VSPGNLDMDDLVSRNIEDDLYQRGLE        1489
GhVIRA       ----AVEVDDAILYGGLSFPQNN-VQ--------VPSGIQHFGQGLDGKADDSLYSGGFE        1517
GhVIRD       ----AVEVDDAILYGSLSFPQNN-VQ--------VPSGIQHFGQGLDGKADDSLYSGGFE        1516
                    : . *   .                 .  .   :.:  .      .    .:.

OsVIR        KRMVWELPDCSIDKQLIPSQSARRKLALGDSASRRVRDNQTH---EPSGQFSRGLNTPSA        1514
AtVIR        DKFWWECPETLPERLPQSSLPAKRKLPTLESSSRRAKGENSSVDIPTQNSIQRGMGSVSL        1549
GhVIRA       DKFSWELPETLPGRLLQTALPTRRKLQAADSANRSARGDNSVAEITNPTAFQRGLGPSTA        1577
GhVIRD       DRFSWELPETLPGRLLQTALPTRRKLQAADSANRSARGDNSVAEITNPTAFQRGLGPSTA        1576
             .:: ** *:     :     :  ::***    :*:.* .:.:::          :.**:.   :

OsVIR        SIGHTRRDTFRQRKPNTSRPPSMHVDDYVARERNIEGASSASNIVSSTPRGALSGRPPSI        1574
AtVIR        PPAPTRRDAFRQRKPNTSRPPSMHVDDYVARERSVDTAGNS-NAITISRAGSSSGRPPSI        1608
GhVIRA       SSGTTRRDSFRQRKPNTSRPPSMHVDDYVARERSVDGVSNS-NVIAVPRVGSSGGRPPSI        1636
GhVIRD       SSGTTRRDSFRQRKPNTSRPPSMHVDDYVARERSVDGVSNS-NVIAVPRVGSSGGRPPSI        1635
               .  ****:************************.:: ...: * ::     *: .******

OsVIR        HVDEFMARQRERQNPVLAPS--GDATQVRSKATLDDNVSTKPEKPRQPKADLDDD-QEIN        1631
AtVIR        HVDEFMARQRERGQNPSTIVVGEAVVQVKNPTPARD-TEKVAGKPKQFKADPDDDLQGID        1667
GhVIRA       HVDEFMARQRERQNPAASGT--ETATQSKNAAPINGPDNEKVNKSKQLKSDLDDDLQGID        1694
GhVIRD       HVDEFMARQRERQNPAASGT--ETAAQSKNAAPINGPDNEKVNKSKQLKSDLDDDLQGID        1693
             ************ :    :       ..* :.  :    .    .      * :* *:* *** * *:

OsVIR        IIFDEE--SGSDDKLPFPQPDDSLQS-PPVIIGENSPGPVVDETENQQNGINLFSGTVV-        1687
AtVIR        IVFDGEECEGPDDKLPFLQPDENLMQPAPVMVEQNSPHSIVEETESDANGSSQFSHMGTP        1727
GhVIRA       IVFDGEESE-TDDKLPFPQPDDNLQQLAPVIFEQSSPQSVVEETESDVNGSSQFSHMATP        1753
GhVIRD       IVFDGEESE-TDDKLPFPQPDDNLQQPAPVIFEQSSPQSVVEETESDVNGSSQFSHMATP        1752
             *:** *   .   ****** ***:.* .   **:.  :.**   :*:***.: ** .  **    .

OsVIR        --SESDEACETVISSQT-------AIRQESNIPSERKFSVSSPEKVMFPDHADESPFISP        1738
AtVIR        VASNVDENAQSEFSSRISVSRPEMSLIREPSISSDRKFVEQADEAK-------KMAPLKS        1780
GhVIRA       LASNADENAQSEFSSRMSVSRPEMSLTREPSVSSEKKIFEQSDDSK-------NAVSIKN        1806
GhVIRD       LASNADENAQSEFSSRMSVSRPEMSLTREPSVSSDKKFFEQSDDSK-------NAVSIKN        1805
               *: ** .:: :**:         :: :* .: *::*:  .: :           :    :.

OsVIR        TTGLKVIPGYSTHA----------AQ-----ATLRQLPPNMHRKRSPHKLAESSVSSGSH        1783
AtVIR        A-------GISESGFIPA--YHMPGSSGGQNSIDPRVGPQGFYSKSGQQHTGHIHGGFSGR        1831
GhVIRA       SSGFDSASGTNSSGFSAPIYSNTPATSVQLSLDSRITPQNFYPKSSAQYAGNIPVAAGSR        1866
GhVIRD       SGGFDSASGTNSSGFSAPIYSNTPATSVQLPLDSRITPQNFYPKSSAQYAGNIPVAAGSR        1865
             :        * .  .              .           *  * .:: * .  :  ..    . ..:

OsVIR        -GHDRTLYNSQPPLPPMPPPVSSTSLQNPDSIQRQPSSYIARDGPPPFPPSYLMQSFDAC        1842
AtVIR        GVYEQKVMPNQPPLPLVPPPSV-SPVIPHSSD-----------------------S----        1863
GhVIRA       GMYELKVLPNQPPLPPMPPPPTILPVQSDYLS--------SVSGSPS----LLQSS----        1910
GhVIRD       GMYEQKVLPNQPPLPPMPPPPTILPVQSDYLS--------SVSGSPS----LLQSS----        1909
                :: .:  .***** :***      :                             *

OsVIR        MPSFVGHQVQTENVLPSTGDSSSNALPSVDAKFLWSTLPVNRIPMEHLSSGSSTRPVSPL        1902
AtVIR        -------------------------LSNQSSPFISHG---------TQSSGGPTRLMPPL        1889
GhVIRA       -------------------------IPVSDSKFMRTS---------MPSPSGTTRPPPPL        1936
```

```
GhVIRD          -------------------------IPVSDSKFMRTS---------MPSPSGTTRPPPSL      1935
                                         :   .: *:              * ..  **    *

OsVIR           PLRPVLATQH-----AAMDSGPPGSLYNQGGSGVLQPSP-PASLINDATLGTNPASGGAL      1956
AtVIR           PSAIPQYSSNPYASLPPRTSTVQSFGYNHAGVGTTEQQ--QSGPTIDHQSG--NLSVTGM      1945
GhVIRA          PSTPAPFASSPYNLASLNTSASQPALYNQSGMGKTELPKGSIGPTIDARL---PTSAAGL      1993
GhVIRD          PSTPPPFASSPYNLASLNTSASQPALYNQSVMGKTELPQGSIGPTIDARL---PTSAAGL      1992
                *      :.          *      **:.  *  :      .   *        *  .:

OsVIR           ASNSLPSLASQYIIGRPSTP--PFFGTPLQIQLSSG----LAQSVSNPQPS-------LS      2003
AtVIR           TSYPPPNLMPSHNFSRPSSLPVPFYGNP--SHQGGDKPQTMLLVPSIPQSLNTQSIPQLP      2003
GhVIRA          ASYPPPPLMQSLVFNRPPSIPVTPYGTSPALHQGENHPPGILQNPSIPQSSMQT-I--HS      2050
GhVIRD          ASYPPPPLMQSLVFNRPPSIPVTPYGTSPALHQGENHPPSILQNPSIPQSSMQT-I--HS      2049
                :*   * *  .  :.** :     :*.    : . .    :    * **

OsVIR           SMQ--P-RAPPPPPPQPHPSQTFQGSLQQPQEQ----PMPYPLN--------TIQPQVPL      2048
AtVIR           SMQLSQLQRPMQPPQHVRPP--IQISQPSEQGVSMQNPFQIPMHQMQLMQQTQ---VQPY      2058
GhVIRA          LNQLQKLQRPLLPTQHLRPS--MQSSQQLEQVVSSQTPVQMQIQSLPMMHQAHISPVNPY      2108
GhVIRD          LNQLQKLQRPLQPTQHLRPS--MQSSQQLEQVVSSQTPVQMQIQSLPMMHQAHISPVNPY      2107
                  *    : *  *  : :*   :* *    *      *.   ::              *

OsVIR           Q---FPNQLHVPQLQFYHQTQESVLQPIGQSAQQQMDSGMNLNHFFSSPEAIQSLLSDRD      2105
AtVIR           YHPPQQQEISQVQQQQQHHA---VQGQQGAGTSQQQESGMSLHDYFKSPEAIQALLSDRD      2115
GhVIRA          YQPQQP-EFSAAQQQMQVELAQQQAPPQTGGTSQQQDSGMSLHEYFQSPEAIQSLLRDRE      2167
GhVIRD          YLPQQP-EFSAAQQQMQVELAQQQAPPQTGGTSQQQDSGMSLHEYFQSPEAIQSLLRDRE      2166
                       ::   * *   .           .:.** :***.*:.:*.******:** **:

OsVIR           KLCKLLEQNPKLMQMLQDRIGQL      2128
AtVIR           KLCQLLEQHPKLMQMLQEKLGQL      2138
GhVIRA          KLCQLLEQHPKLMQMLQVQFLH-      2189
GhVIRD          KLCQLLEQHPKLMQMLQVQFLH-      2188
                ***:****:******** :: :
```

Figure S1 Amino acid sequence alignment of four plant VIR proteins. Amino acid sequences are aligned by clustal omega server. Identical amino acids are shown in asterisk, and similar amino acids are shown by dot or double dot. The four domains was shaded by four colors.AtVIR: Arabidopsis VIRMA, GhVIRA: *Gossypium hirsutum* VIRMA-A, GhVIRD: *Gossypium hirsutum* VIRMA-D, *OsVIR: Oryza sativa* VIRMA.

```
AtVIR      --MVRSEPCVLFAQTFVHPQLD--EYVDEVIFAEPVIITACEFLEQNAS--SSSQAVSLV   54
GhVIRA     --MGRPEPCVLFSQTFVHTHLD--EYVDEVLFAEPVVITACEFLEQNAS--SASQAVSLV   54
GhVIRD     --MGRPEPCVLFSQTFVHTHLD--EYVDEVLFAEPVVITACEFLEQNAS--SASQAVSLV   54
DmVIR      MADVDDGSELLFFDTFSHEEVT-DINLDLVQFPKPVFITQVRIIPLGARVQADFPGGVRL   59
HsVIR      -MAVDSAMELLFLDTFKHPSAEQSSHIDVVRFPCVVYINEVRVIPPGVRAHSSLPDNRAY   59
                    :** :** *        :* * *   * *.  ..:  ..   :

AtVIR      GATSPPSFALEVFVRCEGES---KFKRLCNPFLYTPSAP--YPLEVEAVVTNHLVVRGSY   109
GhVIRA     GATSPPSFALEVFVQSEGET---RFRRLCQPFLYSHSSS--NVLEVEAVVTNHLVVRGSY   109
GhVIRD     GATSPPSFALEVFVQSEGET---RFRRLCQPFLYSHSSS--NVLEVEAVVTNHLVVRGSY   109
DmVIR      GATNPSKFDLEFFVNDLGMPAASAFENLGL-LRYNQNDCIHLDCSQEKIVTDGLVLRGWY   118
HsVIR      GETSPHTFQLDLFFNNVSKPSAPVFDRLGS-LEYDENTSIIFR-PNSKVNTDGLVLRGWY   117
           * *.* .* *:.*..  .      * .*   : *  .         . : *: **:** *

AtVIR      RSLSLIVYGNIVKDLGQYNII-----------------------------LEGRSVTDIV   140
GhVIRA     RSLSLVIYGNTAEDLGQFNIE-----------------------------FDDSSLTNLV   140
GhVIRD     RSLSLVIYGNTAEDLGQFNIE-----------------------------FDDSSLTNLV   140
DmVIR      STITLAIYGIFTNSVTEPIASPTLPCEPVGPEIANLSGEVLLQEDVLKDEWQEPMQAELL   178
HsVIR      NCLTLAIYGSVDRVISHDRDSPPPPPPPPPPPQPQPSL----KR--------NPKH----   161
             ::* :**   . : .

AtVIR      SSTEGNLEDLPLV-------LHSVNRTIEE----------------------------CL   165
GhVIRA     GSADGKLEDLPLP-------LRAFNRTFEE----------------------------SL   165
GhVIRD     SSADGKLEDLPLP-------LCAFNRTFEE----------------------------SL   165
DmVIR      TAHKGNVSDYDPEDMEYGMSRDHYHQHAEEQEQREMRRLRRSTHSTDHSPPPPRRSHTHS   238
HsVIR      ---------------ADGEKEDQFNGSPPR--------PQPRGPRTPPGPPPPDDDED--   196
                                   :    .

AtVIR      SSLDIVSLPLAAVDLPVEVKRLLQLLLKIF-----DKLATNDVVNKFVDTVVSGVSS---   217
GhVIRA     SSLNVLSLPVVTLDLSVEVKQLLQQMLKIL-----ELPNLGHEVHKVVHTLALAAAS---   217
GhVIRD     -SLNVLSLPVVTLDLSVEVKQLLQQMLKIL-----ELPNLGHEVHEVVHTLALAAAS---   216
DmVIR      ESNDREYIRCSRDKGSRDWSRSPEYSSHRSRRKRSERSRSVVDEHKWPRTPPASIDS-PT   297
HsVIR      ---DPVPLPVSGDKEEDAPHR-EDYFEPISPD-----RNSVPQEGGQYSDEGEVEEEQQEE   247
              :   :     .      :  :                     :          .

AtVIR      --YVTDNVDFFLKNKNCSAVTSSLD-SG-------L------------------------   243
GhVIRA     --FVTFDLESNAINQKHLTSGRNKDFKE-------L------------------------   244
GhVIRD     --FVTFDLDSNAINQKHLTSGRNKDFKE-------L------------------------   243
DmVIR      RPRSPDTMDYED---EDSRSHYKMQSSHYRHSSESLHRGERDRDDEDRSCTPQEQFEPIL   354
HsVIR      GEEDEDDVDVEEEEDEDEDDRRTVDSIP-----EEEEEDEEEEGEEDEEGEGDDGYEQIS   302
                  ::      :      . :

AtVIR      --FHDIVDRVKEDILDLN--------EIQESDVALGLFSFLESETYLATSQQLVVMLSPY   293
GhVIRA     --NHGIS-EARKELLELYE--TLQRKSTNKSSESLTECIFMESDADLASSKQLVEMLSPC   299
GhVIRD     --NHGIS-EAKKELLELYE--TLQRKSMNESSESLTECIFMESDADLASSKQLVEMLSPC   298
DmVIR      SDDEIIGDDEEDDAVDAAAIAEYERELE-----AAAAAAPPAIDAFE-------PWQKPL   402
HsVIR      SDEDGIADLERETF----KYPNFDVEYT---AEDLASVPPMTYDPYD-------RELVPL   348
              . *    ..                               :              *

AtVIR      IQFERDS-----------------------------------------------------   300
GhVIRA     FHFNRSS-----------------------------------------------------   306
GhVIRD     FHFNRSS-----------------------------------------------------   305
DmVIR      LVFEGDMAAHFCKELETLKLLFKKLVL-------QTRCENVNAFSEEHGASVDEREQFVY   455
HsVIR      LYFSCPYKTTFEIEISRMKDQGPDKENSGAIEASVKLTELLDLYREDRGAKWVTA--LEE   406
           : *.

AtVIR      ------------------LCTVLPKL-----------S-------------------KG   311
GhVIRA     ------------------SNFGHGQL-----------P-------------------ES   317
GhVIRD     ------------------SSFGHGQL-----------P-------------------ES   316
DmVIR      LGEQLNNQLGYLAQHYKRRNFVLQQFFGNDELHLRQAANVLQIALSFQAACMQPQPAFKI   515
HsVIR      IPSLIIKGLSYLQLKNTKQ------------DSLGQLVDWTMQALNLQVALR-QPIALNV   453
                                                                     :

AtVIR      KATLLGLSLAFLLCSGREGCLQFVNSG--GMDQLVYLFGHDGQNSTTITLLLLGVVEQAT   369
GhVIRA     KNVILGLNVALFLCSTKESCFHFVNCG--GMDQLAYLFDHQTQNSITITLLLLGVIEQAT   375
GhVIRD     NNVILGLNVALFLCSAKESCFHFVNCG--GMDQLAYLFDHQMQNSITITLLLLGVIEQAT   374
DmVIR      RHIKLGARMAELLGSSEELFQHLLKEHKFDIFEAVFRLYHEPYMALSIKLQLLKAVYALL   575
HsVIR      RQLKAGTKLVSSLAECGA--QGVTGLLQAGVISGLFELLFADHVSSSLKLNAFKALDSVI   511
           .    *  :.  * .       .      .: .  : : .    : ::.*  : .:
```

```
AtVIR      RHSVGCEGFLGWWPREDGSIPSGKSEGYCLLLKLLMQKPCHEIASLAIYILRRLRIYEVI    429
GhVIRA     RHSVGCEGFLGWWPREDENIPSGTSDGYSYLLKLLLQKPRHDVASLATYILHRLRFYEVI    435
GhVIRD     RHSVGCEGFLGWWPREDENIPSGTSDGYSYLLKLLLQKPRHDVASLATYILHRLRFYEVI    434
DmVIR      DTRMGIEHFMGAK-----------NNGYQMIVEAIKTAKLTRTKYALQAIIKKLHLWEGL    624
HsVIR      SMTEGMEAFLRGRQN--------EKSGYQKLLELILLDQTVRVVTAGSAILQKCHFYEVL    563
               * * *:              ..**  ::: :              *::: :::* :

AtVIR      SRYEFAV------LSALEGLSNSHGAATHNLNMLS-------DAKSQLQKLQNLMKSLGS    476
GhVIRA     SRYESEI------LSILGGLSATTKGTNAASNKLR-------GVGSLLKKLLHLVISHGP    482
GhVIRD     SRYESEI------LSILGGLSATTKGTNVASNKLR-------GVGSLLKKLLHLVISHGP    481
DmVIR      ESVQIWCRRLFVDRIIIPGNRDQMEDTVITCQQIE------FAFEMLMDALFSSQLSYLQ    678
HsVIR      SEIKRLGDHLAEKTSSLPNHSEPDHDTDAGLERTNPEYENEVEASMDMDLLESSNISEGE    623
           .  :            : .       :    :               :. *     *

AtVIR      VEDPSPSAYAERSLVSDHSEG-----WLSYKATSKLTSSWTCPFYSSGIDSHILALLKER    531
GhVIRA     IEDPSPVAHASRYFILGQTDG-----LVSYNATSGLIASSNCCFSDWEIDLHLLALLKDR    537
GhVIRD     IEDPSPVAHASRYFILGQTDG-----LVSHKATSGLIASSNCCFSDWEIDLHLLALLKDR    536
DmVIR      PRRFLPVSK-KFE---------------------V----VTDPTAQRSFGNALQSYLGQN    712
HsVIR      IERLINLLEEVFHLMETAPHTMIQQPVKSFPTMAR----ITGPPERDDPYPVLFRYLHSH    679
            .                                      .           :   * ..

AtVIR      GFLPLSAALLSMPELHSKVGDIMDVFTDIAMFIGNIILSFMFSRTGLSFLLHHPE-LTAT    590
GhVIRA     GFLPLSAALLSTTVLHSEAADVVDTSLEIVSSIGSIILSLLFCRSGLVFLLHQPD-LTAT    596
GhVIRD     GFLPLSAALLSTTILHSEAADVVDTSLVIVSSIGSIILSLLFCRSGLVFLLHQPD-LTAT    595
DmVIR      SLAESLLVMLAN-CKELPATT--------YLSMLDLMHTLLRSHVGIDYFVDDAFPVTQT    763
HsVIR      HFLELVTLLLSIPVTSAHPGV--------LQATKDVLKFLAQSQKGLLFFMSEYEA-TNL    730
            :      :*:                       .::  :  .: *: ::: .    *

AtVIR      IIQSLKGSVDLNKEECVPLHY--ASILISKGFT---------------CSLLEIGINLEM    633
GhVIRA     LIHALKGADAMNKEECVPLRY--ASVLISKGFT---------------CNPQEVGIIVET    639
GhVIRD     LIHALKGADAMNKEECVPLRY--ASVLISKGFT---------------CSPQEVGIIVET    638
DmVIR      IVAILLGLDEVPRNPEEKEEKAEKSDAEDKAMEVENEAVEAGGEKPTPPTADEEGKPVA-    822
HsVIR      LIRALCHFY------DQ--------------------------------DEEEGLQSD-    750
           ::  *                                               * *

AtVIR      HLRVVSAVDRLLKSIQQTEEFLWILWELRDVSRSDCGREALLTLGVFPEALA-VLIEALH    692
GhVIRA     HLRVVNAIDRLLSATPQSEEFLWVLWELCGLARSDCGRQALLAMSFFSEVLS-VLIEALH    698
GhVIRD     HLRVVNAIDCLLSATPQSEEFLWVLWELCGLARSDCGRQALLAMSFFSEVLS-VLIEALH    697
DmVIR      -----APISV-----PAPAAAPQVRP--RPILRPVLPRLARLGIEMSYKVQTRYHLDAIA    870
HsVIR      -----GVIDD--------AFALW--------LQDSTQTL---------QCIT-ELFSHFQ    779
                  :.                      :               :  :   :. :

AtVIR      SA---KDMEPAVENSGISPLNLAICHSAA--EIFEVIVSDSTASCLHAWIEHAPVLHKAL    747
GhVIRA     SV---KESEPVIKNSGASPLNLAILHSAA--EIVEVIVTDSTATSLSSWIGHAMELHKAL    753
GhVIRD     SV---KESEPVIKNSGASPLNLAILHSAA--EIVEVIVTDSTATSLSSWIGHAMELHKAL    752
DmVIR      YAAAAPEYDAVKLATHMHAIYSQTCDPAGRQHTVEVLGLNNNLKIFMDLIKKEQRLQTQR    930
HsVIR      RCTASEETDHSDLLGTLHNLYLITFNPVGRSAVGHVFSLEKNLQSLITLMEYYSKEALGD    839
                 : :          :     . ..     .*:  :..   :   :

AtVIR      HTLSPGGSNRKDAPSRLLKWIDAGVVYHKHGVGGLLRYAAVLASGGDAQLS---SSSILA    804
GhVIRA     HSSSPG-SNRKDAPTRLLEWIDAGLVYHKNGAVGLLRYAAVLASGGDAHLT---STNILV    809
GhVIRD     HSSSPG-SNRKDAPTRLLEWIDAGLVYHKNGAVGLLRYAAVLASGGDAHLT---STNILV    808
DmVIR      QLSSPGTKYKSPVLSYAVDMVDACVRYCEQ-LDYLIEHGGVILELAKNHETFEPSVSAVL    989
HsVIR      SK-----SKKSVAYNYACILILVVVQSSSD-VQMLEQHAASLLKLCKADEN-----NAKL    888
                  . :. . .     : . :   ..    * .:.. : .  . . .     .

AtVIR      LDLTPA-ENGAGESTNVSEMNVLDNLGK-----VIFEKSFEGVNLSDSSISQLTTALRIL    858
GhVIRA     SDLTDVVDNIVGESSNASDINVMENLGS-----IISMKSFEGVNLRDSSIAQLTTAFRIL    864
GhVIRD     SDLTDVVDNIVGESSNASDINVMENLGS-----IISMKSFEGVNLRDSSIAQLTTAFRIL    863
DmVIR      QEMYVYMK--PLEAINVFVYDDIMPLVEVIGRSLDYLTTFPG---------DLIMAMRIL    1038
HsVIR      QELGKWLE--PLKNLRF-EINCIPNLIEYVKQNIDNLMTPEGV--------GLTTALRVL    937
            ::    .    :  .    : :  * .     :    :  *          *  *:*:*

AtVIR      ALISDNSTVAA-------------------ALYD-EGAVTVVYAILVNCSFMFERSSNIYD    899
GhVIRA     AFISENPTVAA-------------------ALYD-EGAITVIYVVLVNCSYMLERSSNSYD    905
GhVIRD     AFISENPTVAA-------------------ALYD-EGAITVIYVVLVNCSYMLERSSNSYD    904
DmVIR      RYLSIS-KPLAGQKAPPVTEELKHRFVALQLYAADGVQLCIQIMERLCAYFEQPGAHA--    1095
HsVIR      CNVACPPPPVEG-----QQKDLKWNLAVIQLFSAEGMDTFIRVLQKLNSILTQPWRLH--    990
```

```
               ::                          *:  :*    :  :    : : :

AtVIR          YLVDDDHGCSSISDFLSERNREQSLVDLLIPSLVLLISVLQR-LQGTKEQYRNTKLMKAL     958
GhVIRA         YLVDEGTECNSTSDLLLERNREQCLVDLLIPSLVLLITLLQR-LQEAKEQHKNTKLMTAL     964
GhVIRD         YLVDEGTECNSTSDLLLERNREQCLVDLLIPSLVLLITLLQR-LQEAKEQHKNTKLMNAL     963
DmVIR          --PA-----------LMTIQGVHC-CQIMLPTLQILRELLSYAILCRDGTYKDLTAIDHL     1141
HsVIR          --VNMGTTLH----RVTTISMARC-T------LTLLKTMLTELLRGGSFEFKDMRVPSAL     1037
                              :   .  :.        * :*  :*   :   .  .::      *

AtVIR          LRLHREVSPKLAACAADLSSHYPDSALGFGAVCHLIVSALVCWPVYGWIPGLFHTLLSGV     1018
GhVIRA         LRLHREVSPKLAACAADLSSPYPDSALGFEAVCHLSVSALAYWPVYGWSPGLFHTILASV     1024
GhVIRD         LRLHREVSPKLAACAADLSSPYPDSALGFEAVCHLSVSALAYWPVYGWSPGLFHTILASV     1023
DmVIR          VKVYYLLYYFPTRCQAGPEV----------EQCKM---------------EVVQTLLAYT     1176
HsVIR          VTLHMLLCSIPLSGRLDSDE----------QKIQN---------------DIIDILLTFT     1072
               : ::  :         . .              :                 :.. :*: .

AtVIR          QTSSVPALGP-----KETCSF--LCILSDILPEEGVWFWKSGMPLLSGLRKLAVGTLMGP     1071
GhVIRA         QTTSSLALGP-----KETCSL--LCLLNDLFPEESIWRWKNGMPLLSALRSLAIGTLLGP     1077
GhVIRD         QTTSSLALGP-----KETCSL--LCLLNDLFPEESIWHWKNGMPLLSALRSLAIGTLLGP     1076
DmVIR          QPN----EQDEESLHKSLWTLMIREVLKNV---DGPAHFIPGLKLLAELLPLPLPM----     1225
HsVIR          QGVNEKLTISEETLANNTWSLMLKEVLSSIL--KVPEGFFSGLILLSELLPLPLPM----     1126
               *              :.  ::    :*..:   .    :  *: **: *  * :

AtVIR          QKEKQINWYLEPGPLEK-------LINHLTPNLDKIAKIIQHHAVSALVVIQDMLRVFIV     1124
GhVIRA         HKERQVDWYLECGHLEK-------LFNQLTPHLDRIAQIIQHYAISALVVIQDMLRVFII     1130
GhVIRD         HKERQVDWYLECGHLEK-------LFNQLTPHLDRIAQIIQHYAISALVVIQDMLRVFII     1129
DmVIR          -PQPLCD-QLQQQHKQRLITERKLWSAHLHPQSGQIAKLVEALAPSSFPQLSELLQRVCM     1283
HsVIR          -QTTQV---IEPHDISVALNTRKLWSMHLHVQAKLLQEIVRSFSGTTCQPIQHMLRRICV     1182
                        ::    .           :*  :   : :::.  : ::   :..:*: . :

AtVIR          RIACQRVEHASILLRPIFSSIRDGILDQSSTRDTEA--YMVYRYLNFLASLLEHPHAKGL     1182
GhVIRA         RIACQKAEQASKLLRPILSWIHDHSSDLSSLSDTEA--YKVYRCLDFLTSLLEHPYAKVL     1188
GhVIRD         RIACQKAEQASKLLRPILSWIHDHTSDLSSLSDTEA--YKVYRCLDFLTSLLEHPYAKVL     1187
DmVIR          QLSDLAPNMTLLIAKTITELLCNEYQTSNC-IPT----TNLERLLRFSTRLCAFAPLKSS     1338
HsVIR          QLCDLASPTALLIMRTVLDLIVEDLQSTSE-DKEKQYTSQTTRLLALLDALASHKACKLA     1241
               ::.      :  : : : . : :     .             * * :   *  .   *

AtVIR          LLE---------EGIVQLLVEVLERCYDATYPSENRVLEY--GIVSASSVIQWCIPAFRS     1231
GhVIRA         LVG---------EGFPQILTRVLESCFDATDSDGRQASDCRDSAKYGVALISLCIPVFKS     1239
GhVIRD         LVG---------EGFPQILTRVLESCFDATDSDGRQASDCRDFAKYGFALISLCIPVFKS     1238
DmVIR          MLSILS------GKFWELFQSLLALN----------------EF---NDVVSNCQEAVHR     1373
HsVIR          ILHLINGTIKGDERYAEIFQDLLALV----------------RSPGDSVIRQQCVEYVTS     1285
               ::              :::  :*                          : . *   .

AtVIR          I-SLLCDSQVPLLCFQK--KELLASLSAKDCALIFPFVLKFCQVLPVGNELLS-CLGAFK     1287
GhVIRA         I-SLLCSSRTFSQYDERHEMHKFDSLSPKDCSIFINQLLKFCQVLPVGKELVS-CLTAFR     1297
GhVIRD         I-SLLCSSLTFPQYDERHEMHKFDSLSPKDCSIFINQLLKFCQVLPVGKELVS-CLTAFR     1296
DmVIR          ILDSFLDSGISLISHKS-------------TA---SPALNLAAALPPKELIPRIIDAVFS     1417
HsVIR          ILQSLCDQDIALILPSS-------------SEGSISELEQLSNSLPNKELMTSICDCLLA     1332
               * . : ..       .                       ::.  **  : :       :

AtVIR          DLSSCGEGQDGLVSLLFHLFSGTEESVSERWCD---TNSLSLDQLDMKKNPPFLSCWIKL     1344
GhVIRA         DMGSCTEGCNALLSALLNSSSSTHDELESERGNEKNVNFHFLNESEWRKSPPLLCCWIKL     1357
GhVIRD         DMGSCTEGRNALLSALLYSSSSTHDELESERGNEKNVNFHFLNESEWRKSPPLLCCWIKL     1356
DmVIR          NLTSVEVTHGISILAVRNLVI----------LTEHDFTFYHL-AQLLKQKITEFQAWMER     1466
HsVIR          TLANSESSYNCLLTCVRTMMF----------LAEHDYGLFHL-KSSLRKNSSALHSLLKR     1381
                : .     .  :  :                         *     ::.   : . ::

AtVIR          LNSINSKDGL----SSLAMKAVNVLSVGSIRLCLDGKSLDSKKV-----------AALKS     1389
GhVIRA         LKSIDSKDHL----PSYTLEAANVLSLGTLGFCMGGNSLNMNSV-----------VALKF     1402
GhVIRD         LKSIDSKDHL----PPYTLEAANVLALGTLGFCMGGNSWNMNSV-----------VALKF     1401
DmVIR          VILHNETVEYNANIESLILLLRSLTQIEPP----------PAMSAMPHRTLKLGATELAQ     1516
HsVIR          VVSTFSKDT-GELASSFLEFMRQILNSDTIGCCGDDNGLMEVEGAHTSRTMSINAAELKQ     1440
               :    ..               .:                               . *

AtVIR          LFGLPSEF-------SGTDT--------FREENIGLIEQMVTLLSSMTSGS---------     1425
GhVIRA         LFGLPDDT-------AGIGG--------FPEDNIKYIQEFSTLLSSRIDND---------     1438
GhVIRD         LFGLPDDT-------TGIGG--------FPEDNIKYIQEFSTLLSSRIHND---------     1437
DmVIR          LVEFQDIELAKPPVLSRILTVMEKHKAVANEAALSDLKQLILLQASKQEILAGT-----S     1571
```

```
HsVIR          LLQSKEES---------------------PENLFLELEKLVLEHSKDDDNLDSLLDSVVG          1479
               *.   .                        *  :  ::::    :.

AtVIR          --D-------------SS-ATAEMKPYLHEASQSLLS---LL----------KDGNIDDI          1456
GhVIRA         --E-------------DYQTSSDIHISMHQVSESVKSLLLLFQNLT------AAVEVDDA          1477
GhVIRD         --E-------------DYQTSSDIHISMHQVSESVKSLLLLFQNLT------AAVEVDDA          1476
DmVIR          TETPPEAEGEANPSASSCSASLTVEPYLPQAEG----IVTQYEARPIFTRFCATAENAQL          1627
HsVIR          LKQMLESSGDPLPL-----SDQDVEPVLSAPES----LQNLFNNRTAYVL---ADVMDDQ          1527
                                  :   :.  :   .                          :

AtVIR          I-SCKGVF-------VSPGNL--------DMDDLVSRNI-EDDLYQ-RGLEDKFWWECPE          1498
GhVIRA         I-LYGGLSFPQNNVQVPSGIQ--------HFGQGLDGKA-DDSLYS-GGFEDKFSWELPE          1526
GhVIRD         I-LYGSLSFPQNNVQVPSGIQ--------HFGQGLDGKA-DDSLYS-GGFEDRFSWELPE          1525
DmVIR          TARYWLDPLPIEL--IEDMNEPIYERIACDLTDLANVCLN-PDLNVAGDSKRV--MNLS-          1681
HsVIR          LKSMWFTPFQAEE--IDTDL----DLVKVDLIELSEKCCSDFDLH--SELERSF-LSEP-          1577
                              :             .: :  .      .*      :     .

AtVIR          TLPERLPQ--SSLP---A-KRKL-PTLESSSRRAKGENSSVDIPTQNSIQRGMGSVSLPP          1551
GhVIRA         TLPGRLLQ--TALP---T-RRKL-QAADSANRSARGDNSVAEITNPTAFQRGLGPSTASS          1579
GhVIRD         TLPGRLLQ--TALP---T-RRKL-QAADSANRSARGDNSVAEITNPTAFQRGLGPSTASS          1578
DmVIR          GSPQSNREMTPTAPCFRTRRVEVEPA---TGR-----PE---KKMFVSS--VRGRGFARP          1728
HsVIR          SSPGR----TKTTKGFKLGKHKHETFITSSGKSEYIEPA--KRAHVVPPPRGRGRGGFGQ          1631
                 *        :       : :       :.:                     *

AtVIR          APTRRDAFRQRKPNTSRPPSMHVDDYVARERSVDTAGNSNAITISRAGSSSGRPPSIHVD          1611
GhVIRA         GTTRRDSFRQRKPNTSRPPSMHVDDYVARERSVDGVSNSNVIAVPRVGSSGGRPPSIHVD          1639
GhVIRD         GTTRRDSFRQRKPNTSRPPSMHVDDYVARERSVDGVSNSNVIAVPRVGSSGGRPPSIHVD          1638
DmVIR          PPSRGDLFRSRPPNTSRPPSLHVDDFLALETCGAQP---------TGPTGYNKIPSML--          1777
HsVIR          GIRPHDIFRQRKQNTSRPPSMHVDDFVAAESKEVVP---------QDGIPPPKRPLKVSQ          1682
                    * **.*  *******:****::* *                      : *

AtVIR          EFMARQRER-GQNPSTIVVGEAVVQVKNPTPARD-T--E-KVAGKPKQFKADPDDDLQGI          1666
GhVIRA         EFMARQRER-QNPAASGT--ETATQSKNAAPINGPD--N-EKVNKSKQLKSDLDDDLQGI          1693
GhVIRD         EFMARQRER-QNPAASGT--ETAAQSKNAAPINGPD--N-EKVNKSKQLKSDLDDDLQGI          1692
DmVIR          ----RGSRVGRNRGSRISAAAAFRQKKMM-----------RI-GSPSSWA----------          1811
HsVIR          KISSRGGFS-GNRGGRG---AFHSQNRFFTPPASKGNYSRREGTRGSSWS----------          1728
                   *      :  .         * :             .    ..

AtVIR          DIVFDGEECEGPDDKLPFLQPDENLMQPAPVMVEQNSPHSIVEETESDANGSSQFSHMGT          1726
GhVIRA         DIVFDGEESE-TDDKLPFPQPDDNLQQLAPVIFEQSSPQSVVEETESDVNGSSQFSHMAT          1752
GhVIRD         DIVFDGEESE-TDDKLPFPQPDDNLQQPAPVIFEQSSPQSVVEETESDVNGSSQFSHMAT          1751
DmVIR          ---------------------------------ESP---GS---YRSASD--SHFSSSD-          1829
HsVIR          ---------------------------------AQNTPRGN---YNESRGGQSNFNRGPL          1752
                                                 .    .     ..  .  *:*.

AtVIR          PVASNVDENAQSEFSSRISVSRPEMSLIREPSISSDRKFVEQADEAKKMAPLKSA-----          1781
GhVIRA         PLASNADENAQSEFSSRMSVSRPEMSLTREPSVSSEKKIFEQSDDSKNAVSIKNSSGFDS          1812
GhVIRD         PLASNADENAQSEFSSRMSVSRPEMSLTREPSVSSDKKFFEQSDDSKNAVSIKNSGGFDS          1811
DmVIR          ---S--------HYSSPHYSGRPRGR----------------------------------          1844
HsVIR          PPLR--------PLSSTGYRPSPRDR----------------------------------          1770
                             **      *.

AtVIR          --GISESGFIPA--YHMPGSSGQNSIDPRVGPQGFYSKSGQQHTGHIHGGFSGRGVYEQK          1837
GhVIRA         ASGTNSSGFSAPIYSNTPATSVQLSLDSRITPQNFYPKSSAQYAGNIPVAAGSRGMYELK          1872
GhVIRD         ASGTNSSGFSAPIYSNTPATSVQLPLDSRITPQNFYPKSSAQYAGNIPVAAGSRGMYEQK          1871
DmVIR          --GLRSR----PSYLR--------------------------------------------          1854
HsVIR          --ASRGRGGLGPSWASANSGSG-------------------GSRGKFVSGGSGRGRHVRS          1809
                 .

AtVIR          VMPNQPPLPLVPPPSV-SPVIPHSSD-----------SLSNQSSPFISHGTQSSGGPTRL          1885
GhVIRA         VLPNQPPLPPMPPPPTILPVQSDYLSSVSGSPSLLQSSIPVSDSKFMRTSMPSPSGTTRP          1932
GhVIRD         VLPNQPPLPPMPPPPTILPVQSDYLSSVSGSPSLLQSSIPVSDSKFMRTSMPSPSGTTRP          1931
DmVIR          ------------------------------------------------------------          1854
HsVIR          FTR---------------------------------------------------------          1812

AtVIR          MPPLPSAIPQYSSNPYASLPPRTSTVQSFGYNHAGVGTTEQQ--QSGPTIDHQSGNLSVT          1943
GhVIRA         PPPLPSTPAPFASSPYNLASLNTSASQPALYNQSGMGKTELPKGSIGPTIDARL-PTSAA          1991
GhVIRD         PPSLPSTPPPFASSPYNLASLNTSASQPALYNQSVMGKTELPQGSIGPTIDARL-PTSAA          1990
```

```
DmVIR       ------------------------------------------------------------       1854
HsVIR       ------------------------------------------------------------       1812

AtVIR       GMTSYPPPNLMPSHNFSRPSSLPVPFYGNP--SHQGGDKPQTMLLVPSIPQSLNTQSIPQ       2001
GhVIRA      GLASYPPPPLMQSLVFNRPPSIPVTPYGTSPALHQGENHPPGILQNPSIPQSSMQT-I--       2048
GhVIRD      GLASYPPPPLMQSLVFNRPPSIPVTPYGTSPALHQGENHPPSILQNPSIPQSSMQT-I--       2047
DmVIR       ------------------------------------------------------------       1854
HsVIR       ------------------------------------------------------------       1812

AtVIR       LPSMQLSQLQRPMQPPQHVRPPIQISQPSEQGVSMQNPFQIPMHQMQLMQQTQ---VQPY       2058
GhVIRA      HSLNQLQKLQRPLLPTQHLRPSMQSSQQLEQVVSSQTPVQMQIQSLPMMHQAHISPVNPY       2108
GhVIRD      HSLNQLQKLQRPLQPTQHLRPSMQSSQQLEQVVSSQTPVQMQIQSLPMMHQAHISPVNPY       2107
DmVIR       ------------------------------------------------------------       1854
HsVIR       ------------------------------------------------------------       1812

AtVIR       YHPPQQQEISQVQQQQQHHA---VQGQQGAGTSQQQESGMSLHDYFKSPEAIQALLSDRD       2115
GhVIRA      YQPQQP-EFSAAQQQMQVELAQQQAPPQTGGTSQQQDSGMSLHEYFQSPEAIQSLLRDRE       2167
GhVIRD      YLPQQP-EFSAAQQQMQVELAQQQAPPQTGGTSQQQDSGMSLHEYFQSPEAIQSLLRDRE       2166
DmVIR       ------------------------------------------------------------       1854
HsVIR       ------------------------------------------------------------       1812

AtVIR       KLCQLLEQHPKLMQMLQEKLGQL    2138
GhVIRA      KLCQLLEQHPKLMQMLQVQFLH-    2189
GhVIRD      KLCQLLEQHPKLMQMLQVQFLH-    2188
DmVIR       -----------------------    1854
HsVIR       -----------------------    1812
```

Figure S6 Amino acid sequence alignment of GhVIRs, AtVIR, DmVIR and HsVIR proteins. Amino acid sequences are aligned by clustal omega server. Identical amino acids are shown in asterisk, and similar amino acids are shown by dot or double dot. The four domains was shaded by four colors. AtVIR: *Arabidopsis* VIRMA, GhVIRA: *Gossypium hirsutum* VIRMA-A, GhVIRD: *Gossypium hirsutum* VIRMA-D, DmVIR: *Drosophila melanogaster* VIRMA, HsVIR: *Homosapiens* VIRMA.
