# Supplementary material for: Cotton (Gossypium hirsutum) VIRMA as an N6-Methyladenosine RNA Methylation Regulator Participates in Controlling Chloroplast-Dependent and Independent Leaf Development
Source: Int J Mol Sci. 2022 Aug 31;23(17):9887. doi: 10.3390/ijms23179887 (PMC9456376; doi:10.3390/ijms23179887)
Supplement: Supplementary file 1 [file ijms-23-09887-s001.zip › Table S1 plant VIRMA amino acid sequences.pdf]

Table S1 plant VIRMA amino acid sequences.

>AtVIR
MVRSEPCVLFAQTFVHPQLDEYVDEVIFAEPVIITACEFLEQNASSSSQAVSLVGATSPPSFA
LEVFVRCEGESKFKRLCNPFLYTPSAPYPLEVEAVVTNHLVVRGSYRSLSLIVYGNIVKDL
GQYNIILEGRSVTDIVSSTEGNLEDLPLVLHSVNRTIEECLSSLDIVSLPLAAVDLPVEVKRL
LQLLLKIFDKLATNDVVNKFVDTVVSGVSSYVTDNVDFFLKNKNCSAVTSSLDSGLFHDI
VDRVKEDILDLNEIQESDVALGLFSFLESETYLATSQQLVVMLSPYIQFERDSLCTVLPKLS
KGKATLLGLSLAFLLCSGREGCLQFVNSGGMDQLVYLFGHDGQNSTTITLLLLGVVEQAT
RHSVGCEGFLGWWPREDGSIPSGKSEGYCLLLKLLMQKPCHEIASLAIYILRRLRIYEVISR
YEFAVLSALEGLSNSHGAATHNLNMLSDAKSQLQKLQNLMKSLGSVEDPSPSAYAERSLV
SDHSEGWLSYKATSKLTSSWTCPFYSSGIDSHILALLKERGFLPLSAALLSMPELHSKVGDI
MDVFTDIAMFIGNIILSFMFSRTGLSFLLHHPELTATIIQSLKGSVDLNKEECVPLHYASILIS
KGFTCSLLEIGINLEMHLRVVSAVDRLLKSIQQTEEFLWILWELRDVSRSDCGREALLTLGV
FPEALAVLIEALHSAKDMEPAVENSGISPLNLAICHSAAEIFEVIVSDSTASCLHAWIEHAPV
LHKALHTLSPGGSNRKDAPSRLLKWIDAGVVYHKHGVGGLLRYAAVLASGGDAQLSSSSI
LALDLTPAENGAGESTNVSEMNVLDNLGKVIFEKSFEGVNLSDSSISQLTTALRILALISDN
STVAAALYDEGAVTVVYAILVNCSFMFERSSNIYDYLVDDDHGCSSISDFLSERNREQSLVD
LLIPSLVLLISVLQRLQGTKEQYRNTKLMKALLRLHREVSPKLAACAADLSSHYPDSALGF
GAVCHLIVSALVCWPVYGWIPGLFHTLLSGVQTSSVPALGPKETCSFLCILSDILPEEGVWF
WKSGMPLLSGLRKLAVGTLMGPQKEKQINWYLEPGPLEKLINHLTPNLDKIAKIIQHHAVS
ALVVIQDMLRVFIVRIACQRVEHASILLRPIFSSIRDGILDQSSTRDTEAYMVYRYLNFLASL
LEHPHAKGLLLEEGIVQLLVEVLERCYDATYPSENRVLEYGIVSASSVIQWCIPAFRSISLLC
DSQVPLLCFQKKELLASLSAKDCALIFPFVLKFCQVLPVGNELLSCLGAFKDLSSCGEGQD
GLVSLLFHLFSGTEESVSERWCDTNSLSLDQLDMKKNPPFLSCWIKLLNSINSKDGLSSLA
MKAVNVLSVGSIRLCLDGKSLDSKKVAALKSLFGLPSEFSGTDTFREENIGLIEQMVTLLSS
MTSGSDSSATAEMKPYLHEASQSLLSLLKDGNIDDIISCKGVFVSPGNLDMDDLVSRNIED
DLYQRGLEDKFWWECPETLPERLPQSSLPAKRKLPTLESSSRRAKGENSSVDIPTQNSIQR
GMGSVSLPPAPTRRDAFRQRKPNTSRPPSMHVDDYVARERSVDTAGNSNAITISRAGSSSG
RPPSIHVDEFMARQRERGQNPSTIVVGEAVVQVKNPTPARDTEKVAGKPKQFKADPDDDL
QGIDIVFDGEECEGPDDKLPFLQPDENLMQPAPVMVEQNSPHSIVEETESDANGSSQFSHM
GTPVASNVDENAQSEFSSRISVSRPEMSLIREPSISSDRKFVEQADEAKKMAPLKSAGISES
GFIPAYHMPGSSGQNSIDPRVGPQGFYSKSGQQHTGHIHGGFSGRGVYEQKVMPNQPPLPL
VPPPSVSPVIPHSSDSLSNQSSPFISHGTQSSGGPTRLMPPLPSAIPQYSSNPYASLPPRTSTV
QSFGYNHAGVGTTEQQQSGPTIDHQSGNLSVTGMTSYPPPNLMPSHNFSRPSSLPVPFYG
NPSHQGGDKPQTMLLVPSIPQSLNTQSIPQLPSMQLSQLQRPMQPPQHVRPPIQISQPSEQG
VSMQNPFQIPMHQMQLMQQTQVQPYYHPPQQQEISQVQQQQQHHAVQGQQGAGTSQQ
QESGMSLHDYFKSPEAIQALLSDRDKLCQLLEQHPKLMQMLQEKLGQL
>Gh_A04G1327GhVIR
MGRPEPCVLFSQTFVHTHLDEYVDEVLFAEPVVITACEFLEQNASSASQAVSLVGATSPPSF
ALEVFVQSEGETRFRRLCQPFLYSHSSSNVLEVEAVVTNHLVVRGSYRSLSLVIYGNTAED
LGQFNIEFDDSSLTNLVGSADGKLEDLPLPLRAFNRTFEESLSSLNVLSLPVVTLDLSVEVK
QLLQQMLKILELPNLGHEVHKVVHTLALAAASFVTFDLESNAINQKHLTSGRNKDFKELN
HGISEARKELLELYETLQRKSTNKSSESLTECIFMESDADLASSKQLVEMLSPCFHFNRSSS
NFGHGQLPESKNVILGLNVALFLCSTKESCFHFVNCGGMDQLAYLFDHQTQNSITITLLLL
GVIEQATRHSVGCEGFLGWWPREDENIPSGTSDGYSYLLKLLLQKPRHDVASLATYILHRL
RFYEVISRYESEILSILGGLSATTKGTNAASNKLRGVGSLLKKLLHLVISHGPIEDPSPVAHA
SRYFILGQTDGLVSYNATSGLIASSNCCFSDWEIDLHLLALLKDRGFLPLSAALLSTTVLHS
EAADVVDTSLEIVSSIGSIILSLLFCRSGLVFLLHQPDLTATLIHALKGADAMNKEECVPLRY
ASVLISKGFTCNPQEVGIIVETHLRVVNAIDRLLSATPQSEEFLWVLWELCGLARSDCGRQ
ALLAMSFFSEVLSVLIEALHSVKESEPVIKNSGASPLNLAILHSAAEIVEVIVTDSTATSLSS
WIGHAMELHKALHSSSPGSNRKDAPTRLLEWIDAGLVYHKNGAVGLLRYAAVLASGGDA
HLTSTNILVSDLTDVVDNIVGESSNASDINVMENLGSIISMKSFEGVNLRDSSIAQLTTAFRI
LAFISENPTVAAALYDEGAITVIYVVLVNCSYMLERSSNSYDYLVDEGTECNSTSDLLLER
NREQCLVDLLIPSLVLLITLLQRLQEAKEQHKNTKLMTALLRLHREVSPKLAACAADLSSP
YPDSALGFEAVCHLSVSALAYWPVYGWSPGLFHTILASVQTTSSLALGPKETCSLLCLLND
LFPEESIWRWKNGMPLLSALRSLAIGTLLGPHKERQVDWYLECGHLEKLFNQLTPHLDRI
AQIIQHYAISALVVIQDMLRVFIIRIACQKAEQASKLLRPILSWIHDHSSDLSSLSDTEAYKV
YRCLDFLTSLLEHPYAKVLLVGEGFPQILTRVLESCFDATDSDGRQASDCRDSAKYGVALIS
LCIPVFKSISLLCSSRTFSQYDERHEMHKFDSLSPKDCSIFINQLLKFCQVLPVGKELVSCLT
AFRDMGSCTEGCNALLSALLNSSSSTHDELESERGNEKNVNFHFLNESEWRKSPPLLCCW
IKLLKSIDSKDHLPSYTLEAANVLSLGTLGFCMGGNSLNMNSVVALKFLFGLPDDTAGIGG
FPEDNIKYIQEFSTLLSSRIDNDEDYQTSSDIHISMHQVSESVKSLLLLFQNLTAAVEVDDAI
LYGGLSFPQNNVQVPSGIQHFGQGLDGKADDSLYSGGFEDKFSWELPETLPGRLLQTALP
TRRKLQAADSANRSARGDNSVAEITNPTAFQRGLGPSTASSGTTRRDSFRQRKPNTSRPPS
MHVDDYVARERSVDGVSNSNVIAVPRVGSSGGRPPSIHVDEFMARQRERQNPAASGTETA
TQSKNAAPINGPDNEKVNKSKQLKSDLDDDLQGIDIVFDGEESETDDKLPFPQPDDNLQQ
LAPVIFEQSSPQSVVEETESDVNGSSQFSHMATPLASNADENAQSEFSSRMSVSRPEMSLT
REPSVSSEKKIFEQSDDSKNAVSIKNSSGFDSASGTNSSGFSAPIYSNTPATSVQLSLDSRITP

QNFYPKSSAQYAGNIPVAAGSRGMYELKVLPNQPPLPPMPPPPTILPVQSDYLSSVSGSPSL
LQSSIPVSDSKFMRTSMPSPSGTTRPPPPLPSTPAPFASSPYNLASLNTSASQPALYNQSGMG
KTELPKGSIGPTIDARLPTSAAGLASYPPPPLMQSLVFNRPPSIPVTPYGTSPALHQGENHPP
GILQNPSIPQSSMQTIHSLNQLQKLQRPLLPTQHLRPSMQSSQQLEQVVSSQTPVQMQIQSL
PMMHQAHISPVNPYYQPQQPEFSAAQQQMQVELAQQQAPPQTGGTSQQQDSGMSLHEY
FQSPEAIQSLLRDREKLCQLLEQHPKLMQMLQVQFLH
>Gh_D04G0886GhVIR
MGRPEPCVLFSQTFVHTHLDEYVDEVLFAEPVVITACEFLEQNASSASQAVSLVGATSPPSF
ALEVFVQSEGETRFRRLCQPFLYSHSSSNVLEVEAVVTNHLVVRGSYRSLSLVIYGNTAED
LGQFNIEFDDSSLTNLVSSADGKLEDLPLPLCAFNRTFEESLSLNVLSLPVVTLDLSVEVKQ
LLQQMLKILELPNLGHEVHEVVHTLALAAASFVTFDLDSNAINQKHLTSGRNKDFKELNH
GISEAKKELLELYETLQRKSMNESSESLTECIFMESDADLASSKQLVEMLSPCFHFNRSSSS
FGHGQLPESNNVILGLNVALFLCSAKESCFHFVNCGGMDQLAYLFDHQMQNSITITLLLLG
VIEQATRHSVGCEGFLGWWPREDENIPSGTSDGYSYLLKLLLQKPRHDVASLATYILHRLR
FYEVISRYESEILSILGGLSATTKGTNVASNKLRGVGSLLKKLLHLVISHGPIEDPSPVAHAS
RYFILGQTDGLVSHKATSGLIASSNCCFSDWEIDLHLLALLKDRGFLPLSAALLSTTILHSE
AADVVDTSLVIVSSIGSIILSLLFCRSGLVFLLHQPDLTATLIHALKGADAMNKEECVPLRYA
SVLISKGFTCSPQEVGIIVETHLRVVNAIDCLLSATPQSEEFLWVLWELCGLARSDCGRQAL
LAMSFFSEVLSVLIEALHSVKESEPVIKNSGASPLNLAILHSAAEIVEVIVTDSTATSLSSWIG
HAMELHKALHSSSPGSNRKDAPTRLLEWIDAGLVYHKNGAVGLLRYAAVLASGGDAHLT
STNILVSDLTDVVDNIVGESSNASDINVMENLGSIISMKSFEGVNLRDSSIAQLTTAFRILAFI
SENPTVAAALYDEGAITVIYVVLVNCSYMLERSSNSYDYLVDEGTECNSTSDLLLERNREQ
CLVDLLIPSLVLLITLLQRLQEAKEQHKNTKLMNALLRLHREVSPKLAACAADLSSPYPDS
ALGFEAVCHLSVSALAYWPVYGWSPGLFHTILASVQTTSSLALGPKETCSLLCLLNDLFPE
ESIWHWKNGMPLLSALRSLAIGTLLGPHKERQVDWYLECGHLEKLFNQLTPHLDRIAQII
QHYAISALVVIQDMLRVFIIRIACQKAEQASKLLRPILSWIHDHTSDLSSLSDTEAYKVYRC
LDFLTSLLEHPYAKVLLVGEGFPQILTRVLESCFDATDSDGRQASDCRDFAKYGFALISLCIP
VFKSISLLCSSLTFPQYDERHEMHKFDSLSPKDCSIFINQLLKFCQVLPVGKELVSCLTAFRD
MGSCTEGRNALLSALLYSSSSTHDELESERGNEKNVNFHFLNESEWRKSPPLLCCWIKLL
KSIDSKDHLPPYTLEAANVLALGTLGFCMGGNSWNMNSVVALKFLFGLPDDTTGIGGFPE
DNIKYIQEFSTLLSSRIHNDEDYQTSSDIHISMHQVSESVKSLLLLFQNLTAAVEVDDAILYG
SLSFPQNNVQVPSGIQHFGQGLDGKADDSLYSGGFEDRFSWELPETLPGRLLQTALPTRRK
LQAADSANRSARGDNSVAEITNPTAFQRGLGPSTASSGTTRRDSFRQRKPNTSRPPSMHVD
DYVARERSVDGVSNSNVIAVPRVGSSGGRPPSIHVDEFMARQRERQNPAASGTETAAQSK
NAAPINGPDNEKVNKSKQLKSDLDDDLQGIDIVFDGEESETDDKLPFPQPDDNLQQPAPVI
FEQSSPQSVVEETESDVNGSSQFSHMATPLASNADENAQSEFSSRMSVSRPEMSLTREPSV
SSDKKFFEQSDDSKNAVSIKNSGGFDSASGTNSSGFSAPIYSNTPATSVQLPLDSRITPQNFY
PKSSAQYAGNIPVAAGSRGMYEQKVLPNQPPLPPMPPPPTILPVQSDYLSSVSGSPSLLQSSI
PVSDSKFMRTSMPSPSGTTRPPPSLPSTPPPFASSPYNLASLNTSASQPALYNQSVMGKTEL
PQGSIGPTIDARLPTSAAGLASYPPPPLMQSLVFNRPPSIPVTPYGTSPALHQGENHPPSILQ
NPSIPQSSMQTIHSLNQLQKLQRPLQPTQHLRPSMQSSQQLEQVVSSQTPVQMQIQSLPM
MHQAHISPVNPYYLPQQPEFSAAQQQMQVELAQQQAPPQTGGTSQQQDSGMSLHEYFQS
PEAIQSLLRDREKLCQLLEQHPKLMQMLQVQFLH
>Brara.C03117.1
MVRSEPCVLFAQTFVHPQLDEYVDEVIFAEPVIITACEFLEQNASSSSQAVSLLGATSPPSFA
LEVFVRCEGESKFKRLCNPFLYTPSAPYPLEVEAVVTNHLVVRGSYRSLSLIVYGNIVKDL
GQYNIILEGRSVTDIVNSTEGNLEDLPLVLHSVNRTIEECLSSLDIVSLPLAAVDVPVEVKRL
LQLLVKVFDQLATDDVLNKFVDTVVSGVSSYVTDNVDFFLKNKNCTAVASSVDSGIFHDI
TDKVKKDILDLNEIQESDVPLGSSELLSFLESETNLATSQQLVDMLSPYIQFESDSLCTAFPQ
LSKGKATLLGLSLAFLLCSGREGCLHFVNSGGMDQLVFLFGHDVQNSTTITLLLLGVVEQ
ATRHAVGCEGFLGWWPREDGSIPSGKSEGYCLLLKLLMQKPCHEVASLAIYILHRLRIYEI
VSRYEFAVLSALESLSNSHGAATHNLNMLSDAKSQLQKLQKLMNSLGSVEDPSPSAYAER
SLVFDHSEGWLSYKATSKLTASWACPFSNSGTDSHMLSLLKERGFLPLSAALLSIPGLHSEL
GDILDVFTDIAMFIGNIILSLMFSRTGLSFLLHHSQLTATIIQSLKGSVDLNKEECVPLRYASV
LISKGFTCSLLEIGINLEVHLRVVSAVDRLLKSSPQTEEFLGILWELRDVSRSDCGREALLTL
GVFPEALAVLIEALNSVKDTDPAVENSGISPLNLAICHSAAEIFEVIVSDSTVSCLHAWIEHA
PVLHKALHTLSPGGSNRKDAPSRLLKWIDAGVVYHKHGVVGLLRYAAVLASGGDAQLSS
SSILALDLTSAENGVGESSNVSEMNGLDNLEKVILEKSFEGVNLSDSSISQLTTALRILALIS
DNTTVAAALYDEGAVTVVYAILVNCSFMFERSSNIYDYLVDDDHGCSSISDFLSERNREQS
LVDLLIPSLVLLISVLQRLQDSKEQYRNTKLMKALLRLHREVSPKLAACAADLSSHYPDSA
LGFGAVCHLIVSALVCWPVYGWMPGLFHSLLTGFQTSSVPALGPKETCSFLCILSDILPEEG
VWFWKSGMPLLSGLRKLAVGTLMSPQKEKQINWYLEPAPLEKLLNYLTPNLDKIAKIIQH
HAVSALVVIQDMLRVFIVRIACQRVEHASILLRPIFASIREGILDESSTRETEAYKVYRYLNF
LASLLEHPQSKGLLLEEGIVQLLVEVLQRCYDSTYPSEDRVQEFGIVSESSVIRWCIPVFRSI
SLLCHSQVPLSCFPKKELLASLSAKDCASIFPFVLKFCQVLPIGNELLSCLCAFKDLVSCSE
GQDCLVSLLVHLFSGLENPAYDTNNLSLDQVEMKKNPPFLSCWIKLLNSVNSKDGLSVLAI
KAVNVLSVSSIRLCIDGKSLDSKKVAAIKSLFGLPSDFSDTDTFRVENIGLIEQMVTLLSSMT
SGSDTSAIAEMKPCLHEVSQSLLSLLKDGNIDDITSIKIALVSTENFDMNDVDSENIEDDFL
QRGLEDKFWWECPETLPERLPQSSLSAKRKLPTVESSSRRAKGENSSVDIPTQSSIQRVGS

ASLPPAPTRRDTFRQRKTNTSRPPSMHVDDYVARERSIDTAGNSNAITISRAGSSSGRPPSV
HVDEYMARERRGQNPSTIVVGEATAQVKTPTPARETEKAAGKPKQFKADPDDDLQGIDIV
FDGEECEGADDKLPFLQPDENLMQPAPVMVEQNSPHSIVEETESDANGSSQFSHMGTPLA
SNVDENAQSEYSSRISVSRPEMSLIREPSISSDRKFVEQADETNKMVPLKSEPGFVPGYNNI
PGSSGQNLMDPRVGPQGFYSKSSQQQHSGHIQGGFSGRGVYDQKMLPNQPPLPLVPPPSS
SHVMQHSSDSLSNQSSPFSRGTPSSGGGPIRHMPPHPSAIPQYSSNPYASLPPRTSTVQTFG
YNQGGAGTTEQQQQSGPGIDPQPGTGMTSYPPPNLMQSGYSRPFYGNPMHQGGDKQQQ
NMLPVPQSLNPHSIPQQLPSMQLQRPMQPPQHVRPPMQISQPSEQGISLQNQYQIPLHPMQ
MMQQPQVQPYYHPPQQQEISHVQQPQPQQQAVQGQQGAGTSQRQESGMSLHDYLQSPE
TIQALLSNREKLCELLEQNPKLMQMLQEKLGQQ
>GhCSX8308.D04G111100.1
MGRPEPCVLFSQTFVHTHLDEYVDEVLFAEPVVITACEFLEQNASSASQAVSLVGATSPPSF
ALEVFVQSEGETRFRRLCQPFLYSHSSSNVLEVEAVVTNHLVVRGSYRSLSLVIYGNTAED
LGQFNIEFDDSSLTNLVSSADGKLEDLPLPLCAFNRTFEESLSLNVLSLPVVTLDLSVEVKQ
LLQQMLKILELPNLGHEVHEVVHTLALAAASFVTFDLDSNAINQKHLTSGRNKDFKELNH
GISEAKKELLELYETLQRKSMNESSESLTECIFMESDADLASSKQLVEMLSPCFHFNRSSSS
FGHGQLPESNNVILGLNVALFLCSAKESCFHFVNCGGMDQLAYLFDHQMQNSITITLLLLG
VIEQATRHSVGCEGFLGWWPREDENIPSGTSDGYSYLLKLLLQKPRHDVASLATYILHRLR
FYEVISRYESEILSILGGLSATTKGTNVASNKLRGVGSLLKKLLHLVISHGPIEDPSPVAHAS
RYFILGQTDGLVSHKATSGLIASSNCCFSDWEIDLHLLALLKDRGFLPLSAALLSTTILHSE
AADVVDTSLVIVSSIGSIILSLLFCRSGLVFLLHQPDLTATLIHALKGADAMNKEECVPLRYA
SVLISKGFTCSPQEVGIIVETHLRVVNAIDCLLSATPQSEEFLWVLWELCGLARSDCGRQAL
LAMSFFSEVLSVLIEALHSVKESEPVIKNSGASPLNLAILHSAAEIVEVIVTDSTATSLSSWIG
HAMELHKALHSSSPGSNRKDAPTRLLEWIDAGLVYHKNGAVGLLRYAAVLASGGDAHLT
STNILVSDLTDVVDNIVGESSNASDINVMENLGSIISMKSFEGVNLRDSSIAQLTTAFRILAFI
SENPTVAAALYDEGAITVIYVVLVNCSYMLERSSNSYDYLVDEGTECNSTSDLLLERNREQ
CLVDLLIPSLVLLITLLQRLQEAKEQHKNTKLMNALLRLHREVSPKLAACAADLSSPYPDS
ALGFEAVCHLSVSALAYWPVYGWSPGLFHTILASVQTTSSLALGPKETCSLLCLLNDLFPE
ESIWHWKNGMPLLSALRSLAIGTLLGPHKERQVDWYLECGHLEKLFNQLTPHLDRIAQII
QHYAISALVVIQDMLRVFIIRIACQKAEQASKLLRPILSWIHDHTSDLSSLSDTEAYKVYRC
LDFLTSLLEHPYAKVLLVGEGFPQILTRVLESCFDATDSDGRQASDCRDFAKYGFALISLCIP
VFKSISLLCSSLTFPQYDERHEMHKFDSLSPKDCSIFINQLLKFCQVLPVGKELVSCLTAFRD
MGSCTEGRNALLSALLYSSSSTHDELESERGNEKNVNFHFLNESEWRKSPPLLCCWIKLL
KSIDSKDHLPPYTLEAANVLALGTLGFCMGGNSWNMNSVVALKFLFGLPDDTTGIGGFPE
DNIKYIQEFSTLLSSRIHNDEDYQTSSDIHISMHQVSESVKSLLLLFQNLTAAVEVDDAILYG
SLSFPQNNVQVPSGIQHFGQGLDGKADDSLYSGGFEDRFSWELPETLPGRLLQTALPTRRK
LQAADSANRSARGDNSVAEITNPTAFQRGLGPSTASSGTTRRDSFRQRKPNTSRPPSMHVD
DYVARERSVDGVSNSNVIAVPRVGSSGGRPPSIHVDEFMARQRERQNPAASGTETAAQSK
NAAPINGPDNEKVNKSKQLKSDLDDDLQGIDIVFDGEESETDDKLPFPQPDDNLQQPAPVI
FEQSSPQSVVEETESDVNGSSQFSHMATPLASNADENAQSEFSSRMSVSRPEMSLTREPSV
SSDKKFFEQSDDSKNAVSIKNSGGFDSASGTNSSGFSAPIYSNTPATSVQLPLDSRITPQNFY
PKSSAQYAGNIPVAAGSRGMYEQKVLPNQPPLPPMPPPPTILPVQSDYLSSVSGSPSLLQSSI
PVSDSKFMRTSMPSPSGTTRPPPSLPSTPPPFASSPYNLASLNTSASQPALYNQSVMGKTEL
PQGSIGPTIDARLPTSAAGLASYPPPPLMQSLVFNRPPSIPVTPYGTSPALHQGENHPPSILQ
NPSIPQSSMQTIHSLNQLQKLQRPLQPTQHLRPSMQSSQQLEQVVSSQTPVQMQIQSLPM
MHQAHISPVNPYYLPQQPEFSAAQQQMQVELAQQQAPPQTGGTSQQQDSGMSLHEYFQS
PEAIQSLLRDREKLCQLLEQHPKLMQMLQEKLGQL
>GhUGA230.D04G108000.1
MGRPEPCVLFSQTFVHTHLDEYVDEVLFAEPVVITACEFLEQNASSASQAVSLVGATSPPSF
ALEVFVQSEGETRFRRLCQPFLYSHSSSNVLEVEAVVTNHLVVRGSYRSLSLVIYGNTAED
LGQFNIEFDDSSLTNLVSSADGKLEDLPLPLCAFNRTFEESLSLNVLSLPVVTLDLSVEVKQ
LLQQMLKILELPNLGHEVHEVVHTLALAAASFVTFDLDSNAINQKHLTSGRNKDFKELNH
GISEAKKELLELYETLQRKSMNESSESLTECIFMESDADLASSKQLVEMLSPCFHFNRSSSS
FGHGQLPESNNVILGLNVALFLCSAKESCFHFVNCGGMDQLAYLFDHQMQNSITITLLLLG
VIEQATRHSVGCEGFLGWWPREDENIPSGTSDGYSYLLKLLLQKPRHDVASLATYILHRLR
FYEVISRYESEILSILGGLSATTKGTNVASNKLRGVGSLLKKLLHLVISHGPIEDPSPVAHAS
RYFILGQTDGLVSHKATSGLIASSNCCFSDWEIDLHLLALLKDRGFLPLSAALLSTTILHSE
AADVVDTSLVIVSSIGSIILSLLFCRSGLVFLLHQPDLTATLIHALKGADAMNKEECVPLRYA
SVLISKGFTCSPQEVGIIVETHLRVVNAIDCLLSATPQSEEFLWVLWELCGLARSDCGRQAL
LAMSFFSEVLSVLIEALHSVKESEPVIKNSGASPLNLAILHSAAEIVEVIVTDSTATSLSSWIG
HAMELHKALHSSSPGSNRKDAPTRLLEWIDAGLVYHKNGAVGLLRYAAVLASGGDAHLT
STNILVSDLTDVVDNIVGESSNASDINVMENLGSIISMKSFEGVNLRDSSIAQLTTAFRILAFI
SENPTVAAALYDEGAITVIYVVLVNCSYMLERSSNSYDYLVDEGTECNSTSDLLLERNREQ
CLVDLLIPSLVLLITLLQRLQEAKEQHKNTKLMNALLRLHREVSPKLAACAADLSSPYPDS
ALGFEAVCHLSVSALAYWPVYGWSPGLFHTILASVQTTSSLALGPKETCSLLCLLNDLFPE
ESIWHWKNGMPLLSALRSLAIGTLLGPHKERQVDWYLECGHLEKLFNQLTPHLDRIAQII
QHYAISALVVIQDMLRVFIIRIACQKAEQASKLLRPILSWIHDHTSDLSSLSDTEAYKVYRC
LDFLTSLLEHPYAKVLLVGEGFPQILTRVLESCFDATDSDGRQASDCRDFAKYGFALISLCIP
VFKSISLLCSSLTFPQYDERHEMHKFDSLSPKDCSIFINQLLKFCQVLPVGKELVSCLTAFRD

MGSCTEGRNALLSALLYSSSSTHDELESERGNEKNVNFHFLNESEWRKSPPLLCCWIKLL
KSIDSKDHLPPYTLEAANVLALGTLGFCMGGNSWNMNSVVALKFLFGLPDDTTGIGGFPE
DNIKYIQEFSTLLSSRIHNDEDYQTSSDIHISMHQVSESVKSLLLLFQNLTAAVEVDDAILYG
SLSFPQNNVQVPSGIQHFGQGLDGKADDSLYSGGFEDRFSWELPETLPGRLLQTALPTRRK
LQAADSANRSARGDNSVAEITNPTAFQRGLGPSTASSGTTRRDSFRQRKPNTSRPPSMHVD
DYVARERSVDGVSNSNVIAVPRVGSSGGRPPSIHVDEFMARQRERQNPAASGTETAAQSK
NAAPINGPDNEKVNKSKQLKSDLDDDLQGIDIVFDGEESETDDKLPFPQPDDNLQQPAPVI
FEQSSPQSVVEETESDVNGSSQFSHMATPLASNADENAQSEFSSRMSVSRPEMSLTREPSV
SSDKKFFEQSDDSKNAVSIKNSGGFDSASGTNSSGFSAPIYSNTPATSVQLPLDSRITPQNFY
PKSSAQYAGNIPVAAGSRGMYEQKVLPNQPPLPPMPPPPTILPVQSDYLSSVSGSPSLLQSSI
PVSDSKFMRTSMPSPSGTTRPPPSLPSTPPPFASSPYNLASLNTSASQPALYNQSVMGKTEL
PQGSIGPTIDARLPTSAAGLASYPPPPLMQSLVFNRPPSIPVTPYGTSPALHQGENHPPSILQ
NPSIPQSSMQTIHSLNQLQKLQRPLQPTQHLRPSMQSSQQLEQVVSSQTPVQMQIQSLPM
MHQAHISPVNPYYLPQQPEFSAAQQQMQVELAQQQAPPQTGGTSQQQDSGMSLHEYFQS
PEAIQSLLRDREKLCQLLEQHPKLMQMLQEKLGQL
>Gotom.D04G118900.1
MGRPEPCVLFSQTFVHTHLDEYVDEVLFAEPVVITACEFLEQNASSASQAVSLVGATSPPSF
ALEVFVQSEGETRFRRLCQPFLYSHSSSNVLEVEAVVTNHLVVRGSYRSLSLVIYGNTAED
LGQFNIEFDDSSLTNLVSSADGKLEDLPLPLCAFNRTFEESLSLNVLSLPVVTLDLSVEVKQ
LLQQMLKILELPNLGHEVHEVVHTLALAAASFVTFDLDSNAINQKHLTSGRNKDFKELNH
GISEARKELLELYETLQRKSMNESFESLTECIFMESDADLASSKQLVEMLSPCFHFNRSSSS
FGHGQLPESNNVILGLNVALFLCSAKESCFHFVNCGGMDQLAYLFDHQMQNSITITLLLLG
VIEQATRHSVGCEGFLGWWPREDENIPSGTSDGYSYLLKLLLQKPGHDVASLATYILHRLR
FYEVISRYESEILSILGGLSATTKGTNVASNKLRGVGSLLKKLLHLVISHGPIEDPSPVAHAS
RYFILGQTDGLVSYKATSGLIASSNCCFSDWEIDLHLLALLKDRGFLPLSAALLSTTILHSE
AADVVDTSLEIVSSIGSIILSLLFCRSGLVFLLHQPDLTATLIHALKGADAMNKEECVPLRYA
SVLISKGFTCSPQEVGIIVETHLRVVNAIDCLLSATPQSEEFLWVLWELCGLARSDCGRQAL
LAMSFFSEVLSVLIEALHSVKESEPVIKNSGASPLNLAILHSAAEIVEVIVTDSTATSLSSWIG
HAMELHKALHSSSPGSNRKDAPTRLLEWIDAGLVYHKNGAVGLLRYAAVLASGGDAHLT
STNILVSDLTDVVDNIVGESSNASDINVMENLGSIISMKSFEGVNLRDSSIAQLTTAFRILAFI
SENPTVAAALYDEGAITVIYVVLVNCSYMLERSSNSYDYLVDEGTECNSTSDLLLERNREQ
CLVDLLIPSLVLLITLLQRLQEAKEQHKNTKLMNALLRLHREVSPKLAACAADLSSPYPDS
ALGFEAVCHLSVSALAYWPVYGWSPGLFHTILASVQTTSSLALGPKETCSLLCLLNDLFPE
ESIWHWKNGMPLLSALRSLAIGTLLGPHKERQVDWYLECGHLEKLFNQLTPHLDRIAQII
QHYAISALVVIQDMLRVFIIRIACQKAEQASKLLRPILSWIHDHTSDLSSLSDTEAYKVYRC
LDFLTSLLEHPYAKVLLVGEGFPQILTRVLESCFDATDSDGRQASDCRDFAKYGFALISLCIP
VFKSISLLCSSLTFPQYDERHEMHKFDSLSPKDCSIFINQLLKFCQVLPVGKELVSCLTAFRD
MGSCTEGRNALLSALLYSSSSTHDELESERGNEKNVNFHFLNESEWRKSTPLLCCWIKLL
KSIDSKDHLPPYTLEAANVLALGTLGFCMGGNSWNMNSVVALKFLFGLPDDTTGIGGFPE
DNIKYIQEFSTLLSSRIHNDEDYQTSSDIHISMHQVSESVKSLLLLFQNLTAAVEVDDAILYG
SLSFPKNNVQVPSGIQHFGQGLDRKADDSLYSGGFEDRFSWELPETLPGRLLQTALPTRRK
LQAADSANRSARGDNSVAEITNPTAFQRGLGPSTASSGTTRRDSFRQRKPNTSRPPSMHVD
DYVARERSVDGVSNSNVIAVPRVGSSGGRPPSIHVDEFMARQRERQNPAASGTETAAQSK
NAAPINGPDNEKVNKSKQLKSDLDDDLQGIDIVFDGEESETDDKLPFPQPDDNLQQPAPVI
FEQSSPQSVVEETESDVNGSSQFSHMATPLASNADENAQSEFSSRMSVSRPEMSLTREPSV
SSDKKFFEQSDDSKNAVSIKNSGGFDSASGTNSSGFSAPIYSNTPATSVQLPLDSRITPQNFY
PKSSAQYAGNIPVAAGSRGMYEQKVLPNQPPLPPMPPPPTILPVQSDYLSSVSGSPSLLQSSI
PVSDSKFMRTSMPSPSGTTRPPPPLPSTPPPFASSPYNLASLNTSASQPALYNQSVMGKTEL
PQGSIGPTIDARLPTSAAGLASYPPPPLMQSLVFNRPPSIPVTPYGTSPALHQGENHPPSILQ
NPSIPQSSMQTIHSLNQLQKLQRPLQPTQHLRPSMQSSQQLEQVVSSQTPVQMQIQSLPM
MHQAHISPVNPYYLPQQPEFSAAQQQMQVELAQQQAPPQTGGTSQQQDSGMSLHEYFQS
PEAIQSLLRDREKLCQLLEQHPKLMQMLQEKLGQL
>Godar.D04G116500.1
MGRPEPCVLFSQTFVHTHLDEYVDEVLFAEPVVITACEFLEQNASSASQAVSLVGATSPPSF
ALEVFVQSEGETRFRRLCQPFLYSHSSSNVLEVEAVVTNHLVVRGSYRSLSLVIYGNTAED
LGQFNIEFDDSSLTNLVSSADGKLEDLPLPLCAFNRTFEESLSSLNVLSLPVVTLDLSVEVK
QLLQQMLKILELPNLGHEVHEVVHTLALAAASFVTFDLDSNAINQNHLTSGRNKDFKELN
HGISEARKELLELYETLQRKSMNESSESLTECIFMESDADLASSKQLVEMLSPCFHFNRSSS
SFGHGQLPESNNVILGLNVALFLCSAKESCFHFVNCGGMDQLAYLFDHQMQNSITITLLLL
GVIEQATRHSVGCEGFLGWWPREDENIPSGTSDGYSYLLKLLLQKPRHDVASLATYILHRL
RFYEVISRYESEILSILGGLSATTKGTNVASNKLRGVGSLLKKLLHLVISHGPIEDPSPVAHA
SRYFILGQTDGLVSYKATSGLIASSNCCFSDWEIDLHLLALLKDRGFLPLSAALLSTTILHSE
AADVVDTSLEIVSSIGSIILSLLFCRSGLVFLLHQPDLTATLIHALKGADAMNKEECVPLRYA
SVLISKGFTCSPQEVGIIVETHLRVVNAIDCLLSATPQSEEFLWVLWELCGLARSDCGRQAL
LAMSFFSEVLSVLIEALHSVKESEPVIKNSGASPLNLAILHSAAEIVEVIVTDSTATSLSSWIG
HAMELHKALHSSSPGSNRKDAPTRLLEWIDAGLVYHKNGAVGLLRYAAVLASGGDAHLT
STNILVSDLTDVVDNIVGESSNASDINVMENLGSIISMKSFEGVNLRDSSIAQLTTAFRILAFI
SENPTVAAALYDEGAITVIYVVLVNCSYMLERSSNSYDYLVDEGTECNSTSDLLLERNREQ
CLVDLLIPSLVLLITLLQRLQEAKEQHKNTKLMNALLRLHREVSPKLAACAADLSSPYPDS

ALGFEAVCHLSVSALAYWPVYGWSPGLFHTILASVQTTSSLALGPKETCSLLCLLNDLFPE
ESIWHWKNGMPLLSALRSLAIGTLLGPHKERQVDWYLECGHLEKLFNQLTPHLDRIAQII
QHYAISALVVIQDMLRVFIIRIACQKAEQASKLLRPILSWIHDHTSDLSSLSDTEAYKVYRC
LDFLTSLLEHPYAKVLLVGEGFPQILTRVLESCFDATDSDGRQASDCRDFAKYGFALISLCIP
VFKSISLLCSSLTFPQYDERHEMHKFDSLSPKDCSIFINQLLKFCQVLPVGKELVSCLTAFRD
MGSCTEGRNALLSALLYSSSSTHDELESERGNEKNVNFHFLNESEWRKSPPLLCCWIKLL
KSIDSKDHLPPYTLEAANVLALGTLGFCMGGNSWNMNSVVALKFLFGLPDDTTGIGGFPE
DNIKYIQQFSTLLSSRIHNDEDYQTSSDIHISMHQVSESVKSLLLLFQNLTAAVEVDDAILYG
SLSFPQNNVQVPSGIQHFGQGLDGKADDSLYSGGFEDRFSWELPETLPGRLLQTALPTRRK
LQAADSANRSARGDNSVAEITNPTAFQRGLGPSTASSGTTRRDSFRQRKPNTSRPPSMHVD
DYVARERSVDGVSNSNVIAVPRVGSSGGRPPSIHVDEFMARQRERQNPAASGTETAAQSK
NAAPINGPDNEKVNKSKQLKSDLDDDLQGIDIVFDGEESETDDKLPFPQPDDNLQQPAPVI
FEQSSPQSVVEETESDVNGSSQFSHMATPLASNADENAQSEFSSRMSVSRPEMSLTREPSV
SSDKKFFEQSDDSKNAVSIKNSGGFDSASGTNSSGFSAPIYSNTPATSVQLPLDSRITPQNFY
PKSSAQYAGNIPVAAGSRGMYEQKVLPNQPPLPPMPPPPTILPVQSDYLSSVSGSPSLLQSSI
PVSDSKFMRTSMPSPSGTTRPPPPLPSTPPPFASSPYNLASLNTSASQPALYNQSVMGKTEL
PQGSIGPTIDARLPTSAAGLASYPPPPLMQSIVFNRPPSIPVTPYGTSPALHQGENHPPSILQN
PSIPQSSMQTIHSLNQLQKLQRPLQPTQHLRPSMQSSQQLEQVVSSQTPVQMQIQSLPMM
HQAHISPVNPYYLPQQPEFSAAQQQMQVELAQQQAPPQTGGTSQQQDSGMSLHEYFQSP
EAIQSLLRDREKLCQLLEQHPKLMQMLQEKLGQL
>Thecc.05G105900.1
MGRPESCVLFSQTFVHTHLDEYVDEVLFAEPVVITACEFLEQNASSASQAVSLVGATSPPSF
ALEVFVQCEGETRFRRLCQPFLYTHSSSNVLEVEAVVTNHLVVRGSYRSLSLVIYGNTAQD
LGQFNIEFDDSSLPDLVSSADGKLEDLPLALRTINRTFEESLCSLNVISLPVVKLDLSVEVNQ
LLQLMLKILELANVGYAVHKVLSTVASAASSLISFDLDSNAIHQKYLMSERNKDFKELDH
GISEARKNLLELYEALQYKSMNGSSESLTECSFMESEADLASSKQLVEMLLPYFNFNRSSS
SFGHHQLSESKNVILGLNVALFLCSSKESCFHFVNCGGMDQLAYLLDHDMQKSTAITLLLL
GVIEQATRHSVGCEGFLGWWPREDENIPSGTSDGYSHLLKLLLQKPRHDIASLATYVLHR
LRFYEVVSRYEYEVLSILGGLSAAAKGTSVASNKLVGVGSLLKKLLHLVKSHGRIEDPSPV
AHASSFLILGQTDILVSYKATSGLIASSNCCFSNWEIDSHLLALLKDRGFLPLSAALLSTTIL
HSEAEDVVNISMEIVSSIGSIIVSFLFCRSGLVFLLHQPELTATLIHALKGADAMSKEECVPL
RYASVLISKGFTCSPQEVGIIVETHLRVVNAIDRLLSSTPQSEEFLWVLWELCGLARSDCGR
QALLALSFFPEVLSILIEALHSVKETEPAIKNSGAAPLNLAILHSAAEIVEVIVTDSTATSLSS
WIGHAMELHKALHSSPGSNRKDAPTRLLEWIDAGLVYHKNGAIGLLRYAAVLASGGDAH
LTSTNILVSDLTDVVDNVIGESSNASDINVMENLGGIISLKSFDGVSLRDSSIAQLTTAFRILA
FISENPTVAAALYDEGAIAVIYVVLVNCSFMLERSSNNYDYLVDEGTECNSTSDLLLERNR
EQSLVDLLVPSLVLLITLLQKLQEANEQHRNTKLMNALLRLHREVSPKLAACAADLSSPYP
DSALGFEAVCHLVVSALAYWPVYGWTPGLFHSLLASVQATSSLALGPKETCSLMCLLND
MFPEEGVWLWKNGMPLLSALRSLAIGTLLGPLKERQVDWYLERGHLEKLLNQLMPQLD
KIAQIIQHYAISALVVIQDMLRVFIIRIACQKAEHASKLLRPILSWIHDHISDLSSPSDTDAYK
VYRFLDFLASLLEHPYSKAVLLGEGFSQILKRVLESCFVATDSDGKQISDCGNSASCGFTLI
NWCIPVFQSISLLCSSRTFSQNNGRHDMHKFDGLSPKECLLFINQLLKFCQVLPVGKELVS
CLQAFKDLGSCAEGRSAFMSALLHGGNSSGGALESESGHEKNGNFHFQNESELRKSPPLL
CCWKKLLRSVDSKDSSLAYAIEAVNALSLGSLCFCMDGKSLNMNAVVALKFLFGFPDDM
AGIGGLPEENINYIQEFSTLLSSRIINDDYQSPSDMHISMCQVSESVKSLLLLFQISTGTVKV
DDTILNEILSLPQNDVQVPLRIHQMAQGNGGKADDDLYLGGFEDKFSWELPETLPDRLPQ
TALPTRRKLQPADSSTRRARGDNSVTEITNPNAFSRGLGPSTVPPGTTRRDTFRQRKPNTS
RPPSMHVDDYVARERSVDGVTNSNAIAVQRVGSSGGRPPSIHVDEFMARQRERQNPAASV
AETAAQSKNAAPINGADNEKVNKSKQLKTDLDDDLHGIDIVFDGEESETDDKLPFPQPDD
NLQQPASVIVEQSSPHSVVEETESDVNGSSQFSHMGTPLASNVDENAHSEFSSRMSVSRPE
MPLTREPSVSSDKKFFEKSEDSKNAISIKNSSRFDSAAGANSSGFSAPVYSNTPPTSVQLPA
DSRITPQNFYPKSSPQYASNIPGAVGSRGMYEQKVLPNQPPLPPMPPPSAIPPGQSDYLSAV
SGSPSLLQSSLSVSDSKFMRTSMSSPSGNTRPPPPLPSTPPPFASSPYNLASVNASTSQPSVY
NHSGMGKTELPQSSIGPTIDARLPASAAGLTSYPPPLMQSLVFNRPASIPITPYGSTPAQQQG
ENPPSMLQNPSIPQSSIQSMHSLAQLQPLQQLQRPLQPAQHLRPSMQSSQQLDQGVSLQTP
VQMQMQSLQMLQQSHVSPVNPYHQSQQQEFSPAQQQLQVELSQPQVLQQGGGASQQQQ
DSGMSLHEYFQSPEAIQSLLRDREKLCQLLEQHPKLMQMLQEKLGQL
>Gomus.D04G110200.1
MGRPEPCVLFSQTFVHTHLDEYVDEVLFAEPVVITACEFLEQNASSASQAVSLVGATSPPSF
ALEVFVQSEGETRFRRLCQPFLYSHSSSNVLEVEAVVTNHLVVRGSYRSLSLVIYGNTAED
LGQFNIEFDDSSLTNLVSSADGKLEDLPLPLCAFNRTFEESLSSLNVLSLPVVTLDLSVEVK
QLLQQMLKILELPNLGHEVHEVVHTLALAAASFVTFDLDSNAINQKHLTSGRNKDFKELN
HGISEARKELLELYETLQRKSMNESSESLTECIFMESDADLASSKQLVEMLSPCFHFNRSSS
SFGHGQLPESNNVILGLNVALFLCSAKESCFHFVNCGGMDQLAYLFDHQMQNSITITLLLL
GVIEQATRHSVGCEGFLGWWPREDENIPSGTSDGYSYLLKLLLQKPRHDVASLATYILHRL
RFYEVISRYESEILSILGGLSATTKGTNVASNKLRGVGSLLKKLLHLVISHGPIEDPSPVAHA
SRYFILGQTDGLVSYKATSGLIASSNCCFSDWEIDLHLLALLKDRGFLPLSAALLSTTILHSE
AADVVDTSLEIVSSIGSIILSLLFCRSGLVFLLHQPDLTATLIHALKGADAMNKEECVPLRYA
SVLISKGFTCSPQEVGIIVETHLRVVNAIDCLLSATPQSEEFLWVLWELCGLARSDCGRQAL

LAMSFFSEVLSVLIEALHSVKESEPVIKNSGASPLNLAILHSAAEIVEVIVTDSTATSLSSWIG
HAMELHKALHSSSPGSNRKDAPTRLLEWIDAGLVYHKNGAVGLLRYAAVLASGGDAHLT
STNILVSDLTDVVDNIVGESSNASDINVMENLGSIISMKSFEGVNLRDSSIAQLTTAFRILAFI
SENPTVAAALYDEGAITVIYVVLVNCSYMLERSSNSYDYLVDEGTECNSTSDLLLERNREQ
CLVDLLIPSLVLLITLLQRLQEAKEQHKNTKLMNALLRLHREVSPKLAACAADLSSPYPDS
ALGFEAVCHLSVSALAYWPVYGWSPGLFHTILASVQTTSSLALGPKETCSLLCLLNDLFPE
ESIWHWKNGMPLLSALRSLAIGTLLGPHKERQVDWYLECGHLEKLFNQLTPHLDRIAQII
QHYAISALVVIQDMLRVFIIRIACQKAEQASKLLRPILSWIHDHTSDLSSLSDTEAYKVYRC
LDFLTSLLEHPYAKVLLVGEDFPQILTRVLESCFDATDSDGRQASDCRDFAKYGFALISLCIP
VFKSISLLCSSLTFPQYDERHEMHKFDSLSPKDCSIFTNQLLKFCQVLPVGKELVSCLTAFR
DMGSCTEGRNALLSALLYSSSSTHDELESERGNEKNVNFHFLNESEWRKSPPLLCCWIKL
LKSIDSKDHLPPYTLEAANVLALGTLGFCMGGNSWNMNSVVALKFLFGLPDDTTGIGGFP
EDNIKYIQEFSTLLSSRIHNDEDYQTSSDIHISMHQVSESVKSLLLLFQNLTAAVEVDDAILY
GSLSFPQNNVQVPSGIQHFGQGLDGKADDSLYSGGFEDRFSWELPETLPGRLLQTALPTRR
KLQAADSANRSARGDNSVAEITNPTAFQRGLGPSTASSGTTRRDSFRQRKPNTSRPPSMHV
DDYVARERSVDGVSNSNVIAVPRVGSSGGRPPSIHVDEFMARQRERQNPAASGTETAAQS
KNAAPINGPDNEKVNKSKQLKSDLDDDLQGIDIVFDGEESETDDKLPFPQPDDNLQQPAP
VIFEQSSPQSVVEETESDVNGSSQFSHMATPLASNADENAQSEFSSRMSVSRPEMSLTREPS
VSSDKKFFEQSDDSKNAVSIKNSGGFDSASGTNSSGFSAPIYSNTPATSVQLPLDSRITPQNF
YPKSSAQYAGNIPVAAGSRGMYEQKVLPNQPPLPPMPPPPTILPVQSDYLSSVSGSPSLLQS
SIPVSDSKFMRTSMPSPSGTTRPPPPLPSTPPPFASSPYNLASLNTSASQPALYNQSVMGKTE
LPQGSIGPTIDARLPTSAAGLASYPPPPLMQSLVFNRPPSIPVTPYGTSPALHQGENHPPSILQ
NPSIPQSSMQTIHSLNQLQKLQRPLQPTQHLRPSMQSSQQLEQVVSSQTPVQMQIQSLPM
MHQAHISPVNPYYLPQQPEFSAAQQQMQVELAQQQAPPQTGGTSQQQDSGMSLHEYFQS
PEAIQSLLRDREKLCQLLEQHPKLMQMLQEKLGQL
>Gotom.A04G077000.1
MGRPEPCVLFSQTFVHTHLDEYVDEVLFAEPVVITACEFLEQNASSASQAVSLVGATSPPSF
ALEVFVQSEGETRFRRLCQPFLYSHSSSNVLEVEAVVTNHLVVRGSYRSLSLVIYGNTAED
LGQFNIEFDDSSLTNLVSSADGKLEDLPLPLRAFNRTFEESLSSLNVLSLPVVTLDLSVEVK
QLLQQMLKILELPNLGHEVHKVVHTLALAAASFVTFDLESNAINQKHLTSGRNKDFKELN
HGISEARKELLELYETLQRKSTNKSSESLTECIFMESDADLASSKQLVEMLSPCFHFNRSSS
NFGHGQLPESKNVILGLNVALFLCSTKESCFHFVNCGGMDQLAYLFDHQTQNSITITLLLL
GVIEQATRHSVGCEGFLGWWPREDENIPSGTSDGYSYLLKLLLQKPRHDVASLATYILHRL
RFYEVISRYESEILSILGGLSATTKGTNAASNKLRGVGSLLKKLLHLVISHGPIEDPSPVAHA
SRYFILGQTDGLVSYNATSGLIASSNCCFSDWEIDLHLLALLKQDRGFLPLSAALLSTTVLH
SEAADVVDTSLEIVSSIGSIILSLLFCRSGLVFLLHQPDLTATLIHALKGADAMNKEECVPLR
YASVLISKGFTCNPQEVGIIVETHLRVVNAIDRLLSATPQSEEFLWVLWELSGLARSDCGRQ
ALLAMSFFSEVLSVLIEALHSVKESEPVIKNSGASPLNLAILHSAAEIVEVIVTDSTATSLSS
WIGHAMELHKALHSSSPGSNRKDAPTRLLEWIDAGLVYHKNGAVGLLRYAAVLASGGDA
HLTSTNILVSDLTDVVDNIVGESSNASDINVMENLGSIISMKSFEGVNLRDSSIAQLTTAFRI
LAFISENPTVAAALYDEGAITVIYVVLVNCSYMLERSSNSYDYLVDEGTECNSTSDLLLER
NREQCLVDLLIPSLVLLITLLQRLQEAKEQHKNTKLMTALLRLHREVSPKLAACAADLSSP
YPDSALGFEAVCHLSVSALAYWPVYGWSPGLFHTILASVQTTSSLALGPKETCSLLCLLND
LFPEESIWRWKNGMPLLSALRSLAIGTLLGPHKERQVDWYLECGHLEKLFNQLTPHLDRI
AQIIQHYAISALVVIQDMLRVFIIRIACQKAEQASKLLRPILSWIHDHSSDLSSLSDTEAYKV
YRCLDFLTSLLEHPYAKVLLVGEGFPQILTRVLESCFDATDSDGRQASDCRDSAKYGVALIS
LCIPVFKSISLLCSSRTFSQYDERHEMHKFDSLSPKDCSIFINQLLKFCQVLPVGKELVSCLT
AFRDMGSCTEGCNALLSALLNSSSSTHDELESERGNEKNVNFHFLNESEWRKSPPLLCCW
IKLLKSIDSKDHLPPYTLEAANVLSLGTLGFCMGGNSLNMNSVVALKFLFGLPDDTAGIGG
FPEDNIKYIQEFSTLLSSRIDNDEDYQTSSDIHISMHQVSESVKSLLLLFQNLTAAVEVDDAI
LYGGLSFPQNNVQVPSGIQHFGQGLDGKADDSLYSGGFEDKFSWELPETLPGRLLQTALP
TRRKLQAADSANRSARGDNSVAEITNPTAFQRGLGPSTASSGTTRRDSFRQRKPNTSRPPS
MHVDDYVARERSVDGVSNSNVIAVPRVGSSGGRPPSIHVDEFMARQRERQNPAASGTETA
TQSKNAAPINGPDNEKVNKSKQLKSDLDDDLQGIDIVFDGEESETDDKLPFPQPDDNLQQ
LAPVIFEQSSPQSVVEETESDVNGSSQFSHMATPLASNADENAQSEFSSRMSVSRPEMSLT
REPSVSSEKKFFEQSDDSKNAVSIKNSSGFDSASGTNSSGFSAPIYSNTPATSVQLSLDSRITP
QNFYPKSSAQYAGNIPVAAGSRGMYELKVLPNQPPLPPMPPPPTILPVQSDYLSSVSGSPSL
LQSSIPVSDSKFMRTSMPSPSGTTRPPPPLPSTPPPFASSPYNLASLNTSASQPALYNQSGMG
KTELPKGSIGPTIDARLPTSAAGLASYPPPPLMQSLVFNRPPSIPVTPYGTSPALHQGENHPP
GILQNPSIPQSSMQTIHSLNQLQKLQRPLLPTQHLRPSMQSSQQLEQVVSSQTPVQMQIQSL
PMMHQAHISPVNPYYQPQQPEFSAAQQQMQVELAQQQAPPQTGGTSQQQDSGMSLHEY
FQSPEAIQSLLRDREKLCQLLEQHPKLMQMLQEKLGQL
>Gorai.009G393700.1
MGRPEPCVLFSQTFVHTHLDEYVDEVLFAEPVVITACEFLEQNASSASQAVSLVGATSPPSF
ALEVFVQSEGEARFRRLCQPFLYSHSSSNVLEVEAVVTNHLVVRGSYRSLSLVIYGNTAED
LGQFNIEFDDSSLTNLVSSADGKLEDLPLPLCAFNRTFEESLSSLNVLSLPVVTLDLSVEVK
QLLQQMLKILELPNLGHEVHEVVHTLALAAASFVTFDLDSNAINQKHLTSGRNKDFKELN
HGISEARRELLELYETLQRKSMNESSESLTECIFMESDADLASSKQLVEMLSPCFHFNRSSS
SFGHGQLPESNNVILGLNVALFLCSAKESCFHFVNCGGMDQLAYLFDHQMQNSITITLLLL

GVIEQATRHSVGCEGFLGWWPREDENIPSGTSDGYSYLLKLLLQKPRHDVASLATYILHRL
RFYEVISRYESEILSILGGLSATTKGTNVASNKLRGVGSLLKKLLHLVISHGPIEDPSPVAHA
SRYFILGQTDGLVSYKATSGLIASSNCCFSDWEIDLHLLALLKDRGFLPLSAALLSTTILHSE
AADVVDTSLEIVSSIGSIILSLLFCRSGLVFLLHQPDLTATLIHALKGADAMNKEECVPLRYA
SVLISKGFTCSPQEVGIIVETHLRVVNAIDCLLSATPQSEEFLWVLWELCGLARSDCGRQAL
LAMSFFSEVLSVLIEALHSVKESEPVIKNSGASPLNLAILHSAAEIVEVIVTDSTATSLSSWIG
HAMELHKALHSSSPGSNRKDAPTRLLEWIDAGLVYHKNGAVGLLRYAAVLASGGDAHLT
STNILVSDLTDVVDNIVGESSNASDINVMENLGSIISMKSFEGVNLRDSSIAQLTTAFRILAFI
SENPTVAAALYDEGAITVIYVVLVNCSYMLERSSNSYDYLVDEGTECNSTSDLLLERNREQ
CLVDLLIPSLVLLITLLQRLQEAKEQHKNTKLMNALLRLHREVSPKLAACAADLSSPYPDS
ALGFEAVCHLSVSALAYWPVYGWSPGLFHTILASVQTTSSLALGPKETCSLLCLLNDLFPE
ESIWCWKNGMPLLSALRSLAIGTLLGPHKERQVDWYLECGHLEKLFNQLTPHLDRIAQIIQ
HYAISALVVIQDMLRVFIIRIACQKAEQASKLLRPILSWIHDHTSDLSSLSDTEAYKVYRCL
DFLASLLEHPYAKVLLVGEGFPQILTRVLESCFDATDSDGRQASDCRDFAKYGFALISLCIP
VFKSISLLCSSLMFPQYDERHEMHKFDSLSPKDCSIFINQLLKFCQVLPVGKELVSCLTAFR
DMGSCTEGRNALLSALLYSSSSTHDELESERGNEKNVNFHFLNESEWRKSPPLLCCWIKL
LKSIDSKDHLPPYTLEAANVLALGTLGFCMGGNSWNMNSVVALKFLFGLPDDTTGIGGFP
EDNIKYIQEFSTLLSSRIDNDEDYQTSSDIHISMHQVSESVKSLLLLFQNLTAAIEVDDAILY
GSLSFPQNNVQVPSGIQHFGQGLDGKADDSLYSGGFEDRFSWELPETLPGRLLQTALPTRR
KLQAADSANRSARGDNSVAEITNPTAFQRGLGPSTASSGTTRRDSFRQRKPNTSRPPSMHV
DDYVARERSVDGVSNSNVIAVPRVGSSGGRPPSIHVDEFMARQRERQNPAASGTETAAQS
KNAAPINGPDNEKVNKSKQLKSDLDDDLQGIDIVFDGEESETDDKLPFPQPDDNLQQPAP
VIFEQSSPQSVVEETESDVNGSSQFSHMATPLASNADENAQSEFSSRMSVSRPEMSLTREPS
VSSDKKFFEQSDDSKNAVSIKNSSGFDSASGTNSSGFSAPIYSNTPATSVQLPLDSRITPQNF
YPKSSAQYAGNIPVAAGSRGMYEQKVLPNQPPLPPMPPPPTILPVQSDYLSSVSGSPSLLQS
SIPVSDSKFMRTSMPSPSGTTRPPPPLPSTPPPFASSPYNLASLNTSASQPALYNQSVMGKTE
LPQGSIGPTIDARLPTSAAGLASYPPPPLMQSLVFNRPPSIPVTPYGTSPALHQGENHPPSILQ
NPSIPQSSMQTIHSLNQLQKLQRPLQPTQHLRPSMQSSQQLEQVVSSQTPVQMQIQSLPM
MHQAHISPVNPYYLPQQPEFSAAQQQMQVELAQQQAPPQTGGTSQQQDSGMSLHEYFQS
PEAIQSLLRDREKLCQLLEQHPKLMQMLQEKLGQL
>GhCSX8308.A04G071600.1
MGRPEPCVLFSQTFVHTHLDEYVDEVLFAEPVVITACEFLEQNASSASQAVSLVGATSPPSF
ALEVFVQSEGETRFRRLCQPFLYSHSSSNVLEVEAVVTNHLVVRGSYRSLSLVIYGNTAED
LGQFNIEFDDSSLTNLVGSADGKLEDLPLPLRAFNRTFEESLSSLNVLSLPVVTLDLSVEVK
QLLQQMLKILELPNLGHEVHKVVHTLALAAASFVTFDLESNAINQKHLTSGRNKDFKELN
HGISEARKELLELYETLQRKSTNKSSESLTECIFMESDADLASSKQLVEMLSPCFHFNRSSS
NFGHGQLPESKNVILGLNVALFLCSTKESCFHFVNCGGMDQLAYLFDHQTQNSITITLLLL
GVIEQATRHSVGCEGFLGWWPREDENIPSGTSDGYSYLLKLLLQKPRHDVASLATYILHRL
RFYEVISRYESEILSILGGLSATTKGTNAASNKLRGVGSLLKKLLHLVISHGPIEDPSPVAHA
SRYFILGQTDGLVSYNATSGLIASSNCCFSDWEIDLHLLALLKDRGFLPLSAALLSTTVLHS
EAADVVDTSLEIVSSIGSIILSLLFCRSGLVFLLHQPDLTATLIHALKGADAMNKEECVPLRY
ASVLISKGFTCNPQEVGIIVETHLRVVNAIDRLLSATPQSEEFLWVLWELCGLARSDCGRQ
ALLAMSFFSEVLSVLIEALHSVKESEPVIKNSGASPLNLAILHSAAEIVEVIVTDSTATSLSS
WIGHAMELHKALHSSSPGSNRKDAPTRLLEWIDAGLVYHKNGAVGLLRYAAVLASGGDA
HLTSTNILVSDLTDVVDNIVGESSNASDINVMENLGSIISMKSFEGVNLRDSSIAQLTTAFRI
LAFISENPTVAAALYDEGAITVIYVVLVNCSYMLERSSNSYDYLVDEGTECNSTSDLLLER
NREQCLVDLLIPSLVLLITLLQRLQEAKEQHKNTKLMTALLRLHREVSPKLAACAADLSSP
YPDSALGFEAVCHLSVSALAYWPVYGWSPGLFHTILASVQTTSSLALGPKETCSLLCLLND
LFPEESIWRWKNGMPLLSALRSLAIGTLLGPHKERQVDWYLECGHLEKLFNQLTPHLDRI
AQIIQHYAISALVVIQDMLRVFIIRIACQKAEQASKLLRPILSWIHDHSSDLSSLSDTEAYKV
YRCLDFLTSLLEHPYAKVLLVGEGFPQILTRVLESCFDATDSDGRQASDCRDSAKYGVALIS
LCIPVFKSISLLCSSRTFSQYDERHEMHKFDSLSPKDCSIFINQLLKFCQVLPVGKELVSCLT
AFRDMGSCTEGCNALLSALLNSSSSTHDELESERGNEKNVNFHFLNESEWRKSPPLLCCW
IKLLKSIDSKDHLPSYTLEAANVLSLGTLGFCMGGNSLNMNSVVALKFLFGLPDDTAGIGG
FPEDNIKYIQEFSTLLSSRIDNDEDYQTSSDIHISMHQVSESVKSLLLLFQNLTAAVEVDDAI
LYGGLSFPQNNVQVPSGIQHFGQGLDGKADDSLYSGGFEDKFSWELPETLPGRLLQTALP
TRRKLQAADSANRSARGDNSVAEITNPTAFQRGLGPSTASSGTTRRDSFRQRKPNTSRPPS
MHVDDYVARERSVDGVSNSNVIAVPRVGSSGGRPPSIHVDEFMARQRERQNPAASGTETA
TQSKNAAPINGPDNEKVNKSKQLKSDLDDDLQGIDIVFDGEESETDDKLPFPQPDDNLQQ
LAPVIFEQSSPQSVVEETESDVNGSSQFSHMATPLASNADENAQSEFSSRMSVSRPEMSLT
REPSVSSEKKIFEQSDDSKNAVSIKNSSGFDSASGTNSSGFSAPIYSNTPATSVQLSLDSRITP
QNFYPKSSAQYAGNIPVAAGSRGMYELKVLPNQPPLPPMPPPPTILPVQSDYLSSVSGSPSL
LQSSIPVSDSKFMRTSMPSPSGTTRPPPPLPSTPAPFASSPYNLASLNTSASQPALYNQSGMG
KTELPKGSIGPTIDARLPTSAAGLASYPPPPLMQSLVFNRPPSIPVTPYGTSPALHQGENHPP
GILQNPSIPQSSMQTIHSLNQLQKLQRPLLPTQHLRPSMQSSQQLEQVVSSQTPVQMQIQSL
PMMHQAHISPVNPYYQPQQPEFSAAQQQMQVELAQQQAPPQTGGTSQQQDSGMSLHEY
FQSPEAIQSLLRDREKLCQLLEQHPKLMQMLQEKLGQL
>Godar.A04G073500.1
MGRPEPCVLFSQTFVHTHLDEYVDEVLFAEPVVITACEFLEQNASSASQAVSLVGATSPPSF

ALEVFVQSEGETRFRRLCQPFLYSHSSSNVLEVEAVVTNHLVVRGSYRSLSLVIYGNTAED
LGQFNIEFDDSSLTNLVSSADGKLEDLPLPLRAFNRTFEESLSSLNVLSLPVVTLDLSVEVK
QLLQQMLKILELPNLGHEVHKVVHTLALAAASFVTFDLESNAINQKHLTSGRNKDFKELN
HGISEARKELLELYETLQRKSTNKSSESLTECIFMESDADLASSKQLVEMLSPCFHFNRSSS
NFGHGQLPESKNVILGLNVALFLCSTKESCFHFVNCGGMDQLAYLFDHQTQNSITITLLLL
GVIEQATRHSVGCEGFLGWWLREDENIPSGTSDGYSYLLKLLLQKPRHDVASLATYILHRL
RFYEVISRYESEILSILGGLSATTKGTNAASNKLRGVGSLLKKLLHLVISHGPIEDPSPVAHA
SRYFILGQTDGLVSYNATSGLIASSNCCFSDWEIDLHLLALLKDRGFLPLSAALLSTTVLHS
EAADVVDTSLEIVSSIGSIILSLLFCRSGLVFLLHQPDLTATLIHALKGADAMNKEECVPLRY
ASVLISKGFTCNPQEVGIIVETHLRVVNAIDRLLSATPQSEEFLWVLWELCGLARSDCGRQ
ALLAMSFFSEVLSVLIEALHSVKESEPVIKNSGASPLNLAILHSAAEIVEVIVTDSTATSLSS
WIGHAMELHKALHSSSPGSNRKDAPTRLLEWIDAGLVYHKNGAVGLLRYAAVLASGGDA
HLTSTNILVSDLTDVVDNIVGESSNASDINVMENLGSIISMKSFEGVNLRDSSIAQLTTAFRI
LAFISENPTVAAALYDEGAITVIYVVLVNCSYMLERSSNSYDYLVDEGTECNSTSDLLLER
NREQCLVDLLIPSLVLLITLLQRLQEAKEQHKNTKLMTALLRLHREVSPKLAACAADLSSP
YPDSALGFEAVCHLSVSALAYWPVYGWSPGLFHSILASVQTTSSLALGPKETCSLLCLLND
LFPEESIWRWKNGMPLLSALRSLAIGTLLGPHKERQVDWYLECGHLEKLFNQLTPHLDRI
AQIIQHYAISALVVIQDMLRVFIIRIACQKAEQASKLLRPILSWIHDHSSDLSSLSDTEAYKV
YRCLDFLTSLLEHPYAKVLLVGEGFPQILTRVLESCFDATDSDGRQASDCRDSAKYGVALIS
LCIPVFKSISLLCSSRTFSQYDERHEMHKFDSLSPKDCSIFINQLLKFCQVLPVGKELVSCLT
AFRDMGSCTEGCNALLSALLNSSSSTHDELESERGNEKNVNFHFLNESEWRKSPPLLCCW
IKLLKSIDSKDHLPPYTLEAANVLSLGTLGFCMGGNSLNMNSVVALKFLFGLPDDTAGIGG
FPEDNIKYIQEFSTLLSSRIDNDEDYQTSSDIHISMHQVSESVKSLLLLFQNLTAAVEVDDAI
LYGGLSFPQNNVQVPSGIQHFGQGLDGKADDSLYSGGFEDKFSWELPETLPGRLLQTALP
TRRKLQAADSANRSARGDNSVAEITNPTAFQRGLGPSTASSGTTRRDSFRQRKPNTSRPPS
MHVDDYVARERSVDGVSNSNVIAVPRVGSSGGRPPSIHVDEFMARQRERQNPAASGTETA
TQSKNAAPINGPDNEKVNKSKQLKSDLDDDLQGIDIVFDGEESETDDKLPFPQPDDNLQQ
LAPVIFEQSSPQSVVEETESDVNGSSQFSHMATPLASNADENAQSEFSSRMSVSRPEMSLT
REPSVSSEKKFFEQSDDSKNAVSIKNSSGFDSASGTNSSGFSAPIYSNTPATSVQLSLDSRITP
QNFYPKSSAQYAGNIPVAAGSRGMYELKVLPNQPPLPPMPPPPTILPVQSDYLSSVSGSPSL
LQSSIPVSDSKFMRTSMPSPSGTTRPPPPLPSTPPPFASSPYNLASLNTSASQPALYNQSGMG
KTELPKGSIGPTIDARLPTSAAGLASYPPPPLMQSLVFNRPPSIPVTPYGTSPALHQGENHPP
GILQNPSIPQSSMQTIHSLNQLQKLQRPLLPTQHLRPSMQSSQQLEQVVSSQTPVQMQIQSL
PMMHQAHISPVNPYYQPQQPEFSAAQQQMQVELAQQQAPPQTGGTSQQQDSGMSLHEY
FQSPEAIQSLLRDREKLCQLLEQHPKLMQMLQEKLGQL
>GhUGA230.A04G071200.1
MGRPEPCVLFSQTFVHTHLDEYVDEVLFAEPVVITACEFLEQNASSASQAVSLVGATSPPSF
ALEVFVQSEGETRFRRLCQPFLYSHSSSNVLEVEAVVTNHLVVRGSYRSLSLVIYGNTAED
LGQFNIEFDDSSLTNLVGSADGKLEDLPLPLRAFNRTFEESLSSLNVLSLPVVTLDLSVEVK
QLLQQMLKILELPNLGHEVHKVVHTLALAAASFVTFDLESNAINQKHLTSGRNKDFKELN
HGISEARKELLELYETLQRKSTNKSSESLTECIFMESDADLASSKQLVEMLSPCFHFNRSSS
NFGHGQLPESKNVILGLNVALFLCSTKESCFHFVNCGGMDQLAYLFDHQTQNSITITLLLL
GVIEQATRHSVGCEGFLGWWPREDENIPSGTSDGYSYLLKLLLQKPRHDVASLATYILHRL
RFYEVISRYESEILSILGGLSATTKGTNAASNKLRGVGSLLKKLLHLVISHGPIEDPSPVAHA
SRYFILGQTDGLVSYNATSGLIASSNCCFSDWEIDLHLLALLKQDRGFLPLSAALLSTTVLH
SEAADVVDTSLEIVSSIGSIILSLLFCRSGLVFLLHQPDLTATLIHALKGADAMNKEECVPLR
YASVLISKGFTCNPQEVGIIVETHLRVVNAIDRLLSATPQSEEFLWVLWELCGLARSDCGRQ
ALLAMSFFSEVLSVLIEALHSVKESEPVIKNSGASPLNLAILHSAAEIVEVIVTDSTATSLSS
WIGHAMELHKALHSSSPGSNRKDAPTRLLEWIDAGLVYHKNGAVGLLRYAAVLASGGDA
HLTSTNILVSDLTDVVDNIVGESSNASDINVMENLGSIISMKSFEGVNLRDSSIAQLTTAFRI
LAFISENPTVAAALYDEGAITVIYVVLVNCSYMLERSSNSYDYLVDEGTECNSTSDLLLER
NREQCLVDLLIPSLVLLITLLQRLQEAKEQHKNTKLMTALLRLHREVSPKLAACAADLSSP
YPDSALGFEAVCHLSVSALAYWPVYGWSPGLFHTILASVQTTSSLALGPKETCSLLCLLND
LFPEESIWRWKNGMPLLSALRSLAIGTLLGPHKERQVDWYLECGHLEKLFNQLTPHLDRI
AQIIQHYAISALVVIQDMLRVFIIRIACQKAEQASKLLRPILSWIHDHSSDLSSLSDTEAYKV
YRCLDFLTSLLEHPYAKVLLVGEGFPQILTRVLESCFDATDSDGRQASDCRDSAKYGVALIS
LCIPVFKSISLLCSSRTFSQYDERHEMHKFDSLSPKDCSIFINQLLKFCQVLPVGKELVSCLT
AFRDMGSCTEGCNALLSALLNSSSSTHDELESERGNEKNVNFHFLNESEWRKSPPLLCCW
IKLLKSIDSKDHLPSYTLEAANVLSLGTLGFCMGGNSLNMNSVVALKFLFGLPDDTAGIGG
FPEDNIKYIQEFSTLLSSRIDNDEDYQTSSDIHISMHQVSESVKSLLLLFQNLTAAVEVDDAI
LYGGLSFPQNNVQVPSGIQHFGQGLDGKADDSLYSGGFEDKFSWELPETLPGRLLQTALP
TRRKLQAADSANRSARGDNSVAEITNPTAFQRGLGPSTASSGTTRRDSFRQRKPNTSRPPS
MHVDDYVARERSVDGVSNSNVIAVPRVGSSGGRPPSIHVDEFMARQRERQNPAASGTETA
TQSKNAAPINGPDNEKVNKSKQLKSDLDDDLQGIDIVFDGEESETDDKLPFPQPDDNLQQ
LAPVIFEQSSPQSVVEETESDVNGSSQFSHMATPLASNADENAQSEFSSRMSVSRPEMSLT
REPSVSSEKKIFEQSDDSKNAVSIKNSSGFDSASGTNSSGFSAPIYSNTPATSVQLSLDSRITP
QNFYPKSSAQYAGNIPVAAGSRGMYELKVLPNQPPLPPMPPPPTILPVQSDYLSSVSGSPSL
LQSSIPVSDSKFMRTSMPSPSGTTRPPPPLPSTPAPFASSPYNLASLNTSASQPALYNQSGMG
KTELPKGSIGPTIDARLPTSAAGLASYPPPPLMQSLVFNRPPSIPVTPYGTSPALHQGENHPP

GILQNPSIPQSSMQTIHSLNQLQKLQRPLLPTQHLRPSMQSSQQLEQVVSSQTPVQMQIQSL
PMMHQAHISPVNPYYQPQQPEFSAAQQQMQVELAQQQAPPQTGGTSQQQDSGMSLHEY
FQSPEAIQSLLRDREKLCQLLEQHPKLMQMLQEKLGQL
>Gobar.A04G065100.1
MGRPEPCVLFSQTFVHTHLDEYVDEVLFAEPVVITACEFLEQNASSASQAVSLVGATSPPSF
ALEVFVQSEGETRFRRLCQPFLYSHSSSNVLEVEAVVTNHLVVRGSYRSLSLVIYGNTAED
LGQFNIEFDDSSLTNLVSSADGKLEDLPLPLRAFNRTFEESLSSLNVLSLPVVTLDLSVEVK
QLLQQMLKILELPNLGHEVHKVVHTLALAAASFVTFDLESNAINQKHLTSGRNKDFKELN
HGISEARKELLELYETLQRKSTNKSSESLTECIFMESDADLASSKQLVEMLSPCFHFNRSSS
NFGHGQLPESKNVILGLNVALFLCSTKESCFHFVNCGGMDQLAYLFDHQTQNSITITLLLL
GVIEQATRHSVGCEGFLGWWLREDENIPSGTSDGYSYLLKLLLQKLRHDVASLATYILHRL
RFYEVISRYESEILSILGGLSATTKGTNAASNKLRGVGSLLKKLLHLVISHGPIEDPSPVAHA
SRYFILGQTDGLVSYNATSGLIASSNCCFSDWEIDLHLLALLKQDRGFLPLSAALLSTTVLH
SEAADVVDTSLEIVSSIGSIILSLLFCRSGLVFLLHQPDLTATLIHALKGADAMNKEECVPLR
YASVLISKGFTCNPQEVGIIVETHLRVVNAIDRLLSATPQSEEFLWVLWELCGLARSDCGRQ
ALLAMSFFSEVLSVLIEALHSVKESEPVIKNSGASPLNLAILHSAAEIVEVIVTDSTATSLSS
WIGHAMELHKALHSSSPGSNRKDAPTRLLEWIDAGLVYHKNGAVGLLRYAAVLASGGDA
HLTSTNILVSDLTDVVDNIVGESSNASDINVMENLGSIISMKSFEGVNLRDSSIAQLTTAFRI
LAFISENPTVAAALYDEGAITVIYVVLVNCSYMLERSSNSYDYLVDEGTECNSTSDLLLER
NREQCLVDLLIPSLVLLITLLQRLQEAKEQHKNTKLMTALLRLHREVSPKLAACAADLSSP
YPDSALGFEAVCHLSVSALAYWPVYGWSPGLFHSILASVQTTSSLALGPKETCSLLCLLND
LFPEESIWRWKNGMPLLSALRSLAIGTLLGPHKERQVDWYLECGHLEKLFNQLTPHLDRI
AQIIQHYAISALVVIQDMLRVFIIRIACQKAEQASKLLRPILSWIHDHSSDLSSLSDTEAYKV
YRCLDFLTSLLEHPYAKVLLVGEGFPQILTRVLESCFDATDSDGRQASDCRDSAKYGVALIS
LCIPVFKSISLLCSSRTFSQYDERHEMHKFDSLSPKDCSIFINQLLKFCQVLPVGKELVSCLT
AFRDMGSCTEGCNALLSALLNSSSSTHDELESERGNEKNVNFHFLNESEWRKSPPLLCCW
IKLLKSIDSKDHLPPYTLEAANVLSLGTLGFCMGGNSLNMNSVVALKFLFGLPDDTAGIGG
FPEDNIKYIQEFSTLLSSRIDNDEDYQTSSDIHISMHQVSESVKSLLLLFQNLTAAVEVDDAI
LYGGLSFPQNNVQVPSGIQHFGQELDGKADDSLYSGGFEDKFSWELPETLPGRLLQTALPT
RRKLQAADSANRSARGDNSVAEITNPTAFQRGLGPSTASSGTTRRDSFRQRKPNTSRPPSM
HVDDYVARERSVDGVSNSNVIAVPRVGSSGGRPPSIHVDEFMARQRERQNPAASGTETAT
QSKNAAPINGPDNEKVNKSKQLKSDLDDDLQGIDIVFDGEESETDDKLPFPQPDDNLQQL
APVIFEQSSPQSVVEETESDVNGSSQFSHMATPLASNADENAQSEFSSRMSVSRPEMSLTR
EPSVSSEKKFFEQSDDSKNAVSIKNSSGFDSASGTNSSGFSAPIYSNTPATSVQLSLDSRITPQ
NFYPKSSAQYAGNIPVAAGSRGMYELKVLPNQPPLPPMPPPPTILPVQSDYLSSVSGSPSLL
QSSIPVSDSKFLRTSMPSPSGTTRPPPPLPSTPPPFASSPYNLASLNTSASQPALYNQSGMGK
TELPKGSIGPTIDARLPTSAAGLASYPPPPLMQSLVFNRPPSIPVTPYGTSPALHQGENHPPG
ILQNPSIPQSSMQTIHSLNQLQKLQRPLLPTQHLRPSMQSSQQLEQVVSSQTPVQMQIQSLP
MMHQAHISPVNPYYQPQQPEFSAAQQQMQVELAQQQAPPQTGGTSQQQDSGMSLHEYF
QSPEAIQSLLRDREKLCQLLEQHPKLMQMLQEKLGQL
>Gobar.D04G109100.1
MGRPEPCVLFSQTFVHTHLDEYVDEVLFAEPVVITACEFLEQNASSASQAVSLVGATSPPSF
ALEVFVQSEGETRFRRLCQPFLYSHSSSNVLEVEAVVTNHLVVRGSYRSLSLVIYGNTAED
LGQFNIEFDDSSLTNLVSSADGKLEDLPLPLCAFNRTFEESLSSLNVLSLPVVTLDLSVEVK
QLLQQMLKILELPNLGHEVHEVVHTLALAAASFVTFDLDSNAINQNHLTSGRNKDFKELN
HGISEARKELLELYETLQRKSMNESSESLTECIFMESDADLASSKQLVEMLSPCFHFNRSSS
SFGHGQLPESNNVILGLNVALFLCSAKESCFHFVNCGGMDQLAYLFDHQMQNSITITLLLL
GVIEQATRHSVGCEGFLGWWPREDENIPSGTSDGYSYLLKLLLQKPRHDVASLATYILHRL
RFYEVISRYESEILSKLGGLSATTKGTNVASNKLRGVGSLLKKLLHLVISHGPIEDPSPVAHA
SRYFILGQTDGLVSYKATSGLIASSNCCFSDWEIDLHLLALLKDRGFLPLSAALLSTTILHSE
AADVVDTSLEIVSSIGSIILSLLFCRSGLVFLLHQPDLTATLIHALKGADAMNKEECVPLRYA
SVLISKGFTCSPQEVGIIVETHLRVVNAIDCLLSATPQSEEFLWVLWELCGLARSDCGRQAL
LAMSFFFGGIDKCSVLSVLIEALHSVKESEPVIKNSGASPLNLAILHSAAEIVEVIVTDSTAT
SLSSWIGHAMELHKALHSSSPGSNRKDAPTRLLEWIDAGLVYHKNGAVGLLRYAAVLASG
GDAHLTSTNILVSDLTDVVDNIVGESSNASDINVMENLGSIISMKSFEGVNLRDSSIAQLTT
AFRILAFISENPTVAAALYDEGAITVIYVVLVNCSYMLERSSNSYDYLVDEGTECNSTSDLL
LERNREQCLVDLLIPSLVLLITLLQRLQEAKEQHKNTKLMNALLRLHREVSPKLAACAAD
LSSPYPDSALGFEAVCHLSVSALAYWPVYGWSPGLFHTILASVQTTSSLALGPKETCSLLC
LLNDLFPEESIWHWKNGMPLLSALRSLAIGTLLGPHKERQVDWYLECGHLEKLFNQLTPH
LDRIAQIIQHYAISALVVIQDMLRVFIIRIACQKAEQASKLLRPILSWIHDHTSDLSSLSDTEA
YKVYRCLDFLTSLLEHPYAKVLLVGEGFPQILTRVLESCFDATDSDGRQASDCRDFAKYGF
ALISLCIPVFKSISLLCSSLTFPQYDERHEMHKFDSLSPKDCSIFINQLLKFCQVLPVGKELVS
CLTAFRDMGSCTEGRNALLSALLYSSSSTHDELESERGNEKNVNFHFLNESEWRKSPPLLC
CWIKLLKSIDSKDHLPPYTLEAANVLALGTLGFCMGGNSWNMNSVVALKFLFGLPDDTT
GIGGFPEDNIKYIQQFSTLLSSRIHNDEDYQTSSDIHISMHQVSESVKSLLLLFQNLTAAVEV
DDAILYGSLSFPQNNVQVPSGIQHFGQGLDGKADDSLYSGGFEDRFSWELPETLPGRLLQT
ALPTRRKLQAADSANRSARGDNSVAEITNPTAFQRGLGPSTASSGTTRRDSFRQRKPNTSR
PPSMHVDDYVARERSVDGVSNSNVIAVPRVGSSGGRPPSIHVDEFMARQRERQNPAASGT
ETAAQSKNAAPINGPDNEKVNKSKQLKSDLDDDLQGIDIVFDGEESETDDKLPFPQPDDN

LQQPAPTESDVNGSSQFSHMATPLASNADENAQSEFSSRMSVSHKKFFEQSDDSKNAVSIK
NSGGFDSASGTNSSGFSAPIYSNTPATSVQLPLDSRITPQNFYPKSSAQYAGNIPVAAGSRG
MYEQKVLPNQPPLPPMPPPPTILPVQSDYLSSFMRTSMPSPSGTTRPPPPLPSTPPPFASSPY
NLASLNTSASQPALYNQSVMGKNELPQGSIGPTIDALFNRPPSIPVTPSGTSPALHQGENHP
PSILQNPSIPQSSMQTIHSLNQLQKLQRPLQPTQHLRPSIAIITAVGASCVFANPVQMQIQSLP
MMHQAHISPRNNRCKLSLLQQQAPPQTGGTSQQQDSGMSLHEYFQSPEAIQLIGEQHPKL
MQMLQEKLGQL
>Gobar.D04G109200.1
MLKILELPNLGHEVHEVVHTLALAAASFVTFDLDSNAINQNHLTSGRNKDFKELNHGISE
ARKELLELYETLQRKSMNESSESLTECIFMESDADLASSKQLVEMLSPCFHFNRSSSSFGHG
QLPESNNVILGLNVALFLCSAKESCFHFVNCGGMDQLAYLFDHQMQNSITITLLLLGVIEQ
ATRHSVGCEGFLGWWPREDENIPSGTSDGYSYLLKLLLQKPRHDVASLATYILHRLRFYE
VISRYESEILSKLGGLSATTKGTNVASNKLRGVGSLLKKLLHLVISHGPIEDPSPVAHASRYF
ILGQTDGLVSYKATSGLIASSNCCFSDWEIDLHLLALLKDRGFLPLSAALLSTTILHSEAAD
VVDTSLEIVSSIGSIILSLLFCRSGLVFLLHQPDLTATLIHALKGADAMNKEECVPLRYASVLI
SKGFTCSPQEVGIIVETHLRVVNAIDCLLSATPQSEEFLWVLWELCGLARSDCGRQALLAM
SFFSEVLSVLIEALHSVKESEPVIKNSGASPLNLAILHSAAEIVEVIVTDSTATSLSSWIGHA
MELHKALHSSSPGSNRKDAPTRLLEWIDAGLVYHKNGAVGLLRYAAVLASGGDAHLTST
NILVSDLTDVVDNIVGESSNASDINVMENLGSIISMKSFEGVNLRDSSIAQLTTAFRILAFISE
NPTVAAALYDEGAITVIYVVLVNCSYMLERSSNSYDYLVDEGTECNSTSDLLLERNREQCL
VDLLIPSLVLLITLLQRLQEAKEQHKNTKLMNALLRLHREVSPKLAACAADLSSPYPDSAL
GFEAVCHLSVSALAYWPVYGWSPGLFHTILASVQTTSSLALGPKETCSLLCLLNDLFPEESI
WHWKNGMPLLSALRSLAIGTLLGPHKERQVDWYLECGHLEKLFNQLTPHLDRIAQIIQHY
AISALVVIQDMLRVFIIRIACQKAEQASKLLRPILSWIHDHTSDLSSLSDTEAYKVYRCLDFL
TSLLEHPYAKVLLVGEGFPQILTRVLESCFDATDSDGRQASDCRDFAKYGFALISLCIPVFKS
ISLLCSSLTFPQYDERHEMHKFDSLSPKDCSIFINQLLKFCQVLPVGKELVSCLTAFRDMGS
CTEGRNALLSALLYSSSSTHDELESERGNEKNVNFHFLNESEWRKSPPLLCCWIKLLKSID
SKDHLPPYTLEAANVLALGTLGFCMGGNSWNMNSVVALKFLFGLPDDTTGIGGFPEDNIK
YIQQFSTLLSSRIHNDEDYQTSSDIHISMHQVSESVKSLLLLFQNLTAAVEVDDAILYGSLSF
PQNNVQVPSGIQHFGQGLDGKADDSLYSGGFEDRFSWELPETLPGRLLQTALPTRRKLQA
ADSANRSARGDNSVAEITNPTAFQRGLGPSTASSGTTRRDSFRQRKPNTSRPPSMHVDDYV
ARERSVDGVSNSNVIAVPRVGSSGGRPPSIHVDEFMARQRERQNPAASGTETAAQSKNAA
PINGPDNEKVNKSKQLKSDLDDDLQGIDIVFDGEESETDDKLPFPQPDDNLQQPAPVIFEQ
SSPQSVVEETESDVNGSSQFSHMATPLASNADENAQSEFSSRMSVSRPEMSLTREPSVSSD
KKFFEQSDDSKNAVSIKNSGGFDSASGTNSSGFSAPIYSNTPATSVQLPLDSRITPQNFYPKS
SAQYAGNIPVAAGSRGMYEQKVLPNQPPLPPMPPPPTILPVQSDYLSSVSGSPSLLQSSIPVS
DSKFMRTSMPSPSGTTRPPPPLPSTPPPFASSPYNLASLNTSASQPALYNQSVMGKTELPQG
SIGPTIDARLPTSAAGLASYPPPPLMQSIVFNRPPSIPVTPYGTSPALHQGENHPPSILQNPSIP
QSSMQTIHSLNQLQKLQRPLQPTQHLRPSMQSSQQLEQVVSSQTPVQMQIQSLPMMHQA
HISPVNPYYLPQQPEFSAAQQQMQVELAQQQAPPQTGGTSQQQDSGMSLHEYFQSPEAIQ
SLLRDREKLCQLLEQHPKLMQMLQEKLGQL
>Gohir.D04G091700.1
MLKILELPNLGHEVHEVVHTLALAAASFVTFDLDSNAINQKHLTSGRNKDFKELNHGISE
AKKELLELYETLQRKSMNESSESLTECIFMESDADLASSKQLVEMLSPCFHFNRSSSSFGH
GQLPESNNVILGLNVALFLCSAKESCFHFVNCGGMDQLAYLFDHQMQNSITITLLLLGVIE
QATRHSVGCEGFLGWWPREDENIPSGTSDGYSYLLKLLLQKPRHDVASLATYILHRLRFY
EVISRYESEILSILGGLSATTKGTNVASNKLRGVGSLLKKLLHLVISHGPIEDPSPVAHASRY
FILGQTDGLVSHKATSGLIASSNCCFSDWEIDLHLLALLKDRGFLPLSAALLSTTILHSEAA
DVVDTSLVIVSSIGSIILSLLFCRSGLVFLLHQPDLTATLIHALKGADAMNKEECVPLRYASV
LISKGFTCSPQEVGIIVETHLRVVNAIDCLLSATPQSEEFLWVLWELCGLARSDCGRQALLA
MSFFSEVLSVLIEALHSVKESEPVIKNSGASPLNLAILHSAAEIVEVIVTDSTATSLSSWIGH
AMELHKALHSSSPGSNRKDAPTRLLEWIDAGLVYHKNGAVGLLRYAAVLASGGDAHLTS
TNILVSDLTDVVDNIVGESSNASDINVMENLGSIISMKSFEGVNLRDSSIAQLTTAFRILAFIS
ENPTVAAALYDEGAITVIYVVLVNCSYMLERSSNSYDYLVDEGTECNSTSDLLLERNREQC
LVDLLIPSLVLLITLLQRLQEAKEQHKNTKLMNALLRLHREVSPKLAACAADLSSPYPDSA
LGFEAVCHLSVSALAYWPVYGWSPGLFHTILASVQTTSSLALGPKETCSLLCLLNDLFPEE
SIWHWKNGMPLLSALRSLAIGTLLGPHKERQVDWYLECGHLEKLFNQLTPHLDRIAQIIQ
HYAISALVVIQDMLRVFIIRIACQKAEQASKLLRPILSWIHDHTSDLSSLSDTEAYKVYRCL
DFLTSLLEHPYAKVLLVGEGFPQILTRVLESCFDATDSDGRQASDCRDFAKYGFALISLCIPV
FKSISLLCSSLTFPQYDERHEMHKFDSLSPKDCSIFINQLLKFCQVLPVGKELVSCLTAFRD
MGSCTEGRNALLSALLYSSSSTHDELESERGNEKNVNFHFLNESEWRKSPPLLCCWIKLL
KSIDSKDHLPPYTLEAANVLALGTLGFCMGGNSWNMNSVVALKFLFGLPDDTTGIGGFPE
DNIKYIQEFSTLLSSRIHNDEDYQTSSDIHISMHQVSESVKSLLLLFQNLTAAVEVDDAILYG
SLSFPQNNVQVPSGIQHFGQGLDGKADDSLYSGGFEDRFSWELPETLPGRLLQTALPTRRK
LQAADSANRSARGDNSVAEITNPTAFQRGLGPSTASSGTTRRDSFRQRKPNTSRPPSMHVD
DYVARERSVDGVSNSNVIAVPRVGSSGGRPPSIHVDEFMARQRERQNPAASGTETAAQSK
NAAPINGPDNEKVNKSKQLKSDLDDDLQGIDIVFDGEESETDDKLPFPQPDDNLQQPAPVI
FEQSSPQSVVEETESDVNGSSQFSHMATPLASNADENAQSEFSSRMSVSRPEMSLTREPSV
SSDKKFFEQSDDSKNAVSIKNSGGFDSASGTNSSGFSAPIYSNTPATSVQLPLDSRITPQNFY

PKSSAQYAGNIPVAAGSRGMYEQKVLPNQPPLPPMPPPPTILPVQSDYLSSVSGSPSLLQSSI
PVSDSKFMRTSMPSPSGTTRPPPSLPSTPPPFASSPYNLASLNTSASQPALYNQSVMGKTEL
PQGSIGPTIDARLPTSAAGLASYPPPPLMQSLVFNRPPSIPVTPYGTSPALHQGENHPPSILQ
NPSIPQSSMQTIHSLNQLQKLQRPLQPTQHLRPSMQSSQQLEQVVSSQTPVQMQIQSLPM
MHQAHISPVNPYYLPQQPEFSAAQQQMQVELAQQQAPPQTGGTSQQQDSGMSLHEYFQS
PEAIQSLLRDREKLCQLLEQHPKLMQMLQEKLGQL
>Gohir.A04G053650.1
MLKILELPNLGHEVHKVVHTLALAAASFVTFDLESNAINQKHLTSGRNKDFKELNHGISE
ARKELLELYETLQRKSTNKSSESLTECIFMESDADLASSKQLVEMLSPCFHFNRSSSNFGHG
QLPESKNVILGLNVALFLCSTKESCFHFVNCGGMDQLAYLFDHQTQNSITITLLLLGVIEQA
TRHSVGCEGFLGWWPREDENIPSGTSDGYSYLLKLLLQKPRHDVASLATYILHRLRFYEVI
SRYESEILSILGGLSATTKGTNAASNKLRGVGSLLKKLLHLVISHGPIEDPSPVAHASRYFIL
GQTDGLVSYNATSGLIASSNCCFSDWEIDLHLLALLKDRGFLPLSAALLSTTVLHSEAADV
VDTSLEIVSSIGSIILSLLFCRSGLVFLLHQPDLTATLIHALKGADAMNKEECVPLRYASVLIS
KGFTCNPQEVGIIVETHLRVVNAIDRLLSATPQSEEFLWVLWELCGLARSDCGRQALLAMS
FFSEVLSVLIEALHSVKESEPVIKNSGASPLNLAILHSAAEIVEVIVTDSTATSLSSWIGHAM
ELHKALHSSSPGSNRKDAPTRLLEWIDAGLVYHKNGAVGLLRYAAVLASGGDAHLTSTNI
LVSDLTDVVDNIVGESSNASDINVMENLGSIISMKSFEGVNLRDSSIAQLTTAFRILAFISEN
PTVAAALYDEGAITVIYVVLVNCSYMLERSSNSYDYLVDEGTECNSTSDLLLERNREQCLV
DLLIPSLVLLITLLQRLQEAKEQHKNTKLMTALLRLHREVSPKLAACAADLSSPYPDSALG
FEAVCHLSVSALAYWPVYGWSPGLFHTILASVQTTSSLALGPKETCSLLCLLNDLFPEESI
WRWKNGMPLLSALRSLAIGTLLGPHKERQVDWYLECGHLEKLFNQLTPHLDRIAQIIQHY
AISALVVIQDMLRVFIIRIACQKAEQASKLLRPILSWIHDHSSDLSSLSDTEAYKVYRCLDFL
TSLLEHPYAKVLLVGEGFPQILTRVLESCFDATDSDGRQASDCRDSAKYGVALISLCIPVFK
SISLLCSSRTFSQYDERHEMHKFDSLSPKDCSIFINQLLKFCQVLPVGKELVSCLTAFRDMG
SCTEGCNALLSALLNSSSSTHDELESERGNEKNVNFHFLNESEWRKSPPLLCCWIKLLKSI
DSKDHLPSYTLEAANVLSLGTLGFCMGGNSLNMNSVVALKFLFGLPDDTAGIGGFPEDNI
KYIQEFSTLLSSRIDNDEDYQTSSDIHISMHQVSESVKSLLLLFQNLTAAVEVDDAILYGGLS
FPQNNVQVPSGIQHFGQGLDGKADDSLYSGGFEDKFSWELPETLPGRLLQTALPTRRKLQ
AADSANRSARGDNSVAEITNPTAFQRGLGPSTASSGTTRRDSFRQRKPNTSRPPSMHVDD
YVARERSVDGVSNSNVIAVPRVGSSGGRPPSIHVDEFMARQRERQNPAASGTETATQSKNA
APINGPDNEKVNKSKQLKSDLDDDLQGIDIVFDGEESETDDKLPFPQPDDNLQQLAPVIFE
QSSPQSVVEETESDVNGSSQFSHMATPLASNADENAQSEFSSRMSVSRPEMSLTREPSVSS
EKKIFEQSDDSKNAVSIKNSSGFDSASGTNSSGFSAPIYSNTPATSVQLSLDSRITPQNFYPK
SSAQYAGNIPVAAGSRGMYELKVLPNQPPLPPMPPPPTILPVQSDYLSSVSGSPSLLQSSIPV
SDSKFMRTSMPSPSGTTRPPPPLPSTPAPFASSPYNLASLNTSASQPALYNQSGMGKTELPK
GSIGPTIDARLPTSAAGLASYPPPPLMQSLVFNRPPSIPVTPYGTSPALHQGENHPPGILQNP
SIPQSSMQTIHSLNQLQKLQRPLLPTQHLRPSMQSSQQLEQVVSSQTPVQMQIQSLPMMH
QAHISPVNPYYQPQQPEFSAAQQQMQVELAQQQAPPQTGGTSQQQDSGMSLHEYFQSPE
AIQSLLRDREKLCQLLEQHPKLMQMLQEKLGQL
>Potri.016G069100.1
MGRPEPSVLFSQTFVHPQLDEYVDEVLFAEPIVITACEFLEQNASSASQAVSVLGATSPPSF
ALEVFVKCEGETRFRRLCQPFLYSHSSSHVLEVEAVVTNHLVVRGSYRSLSLVIYGNTAED
LGQFSIEFDDSSLTNLVSSAEGKLEDLPLALHSTNRTVEDSLSSLNVLSLPVAASHISAEVKQ
FLQLILKLLELPNLSDSVHRVLTTVVKAVCSFVTRDLCCETVNQKHIKMCGSKNIEEFHHVI
NEARNELLQVLGQVLGDESAELLADCTFLESEADLATSKQLVDMLSQYFSFERNSTNVGA
CQLSQNKSVILGLSLALLLCSGRESCFHFVSSGGMEQLAHIFSNEVQNSSAIILLSLGVVEQ
ATRHPIGCEGFLGWWPREDENIPSGTSKGYSQLLKLVLQRPQHDVASLATYVLHRLRFYE
VVSRYEFSVLSALGGLSALGRVTSVTSAMLNSAKSQLKMLLKLINLRGPIEDPSIAASASRS
LIIGQTEGLLSYKATSNLVGSSHCCFSNWDIDSHLLALLKERGFLPLSAALLSSPILRSEAVD
AMDTFVDIASTIGAILLSLLMCRSGLIFLLNYPELCTTLIDALRGVGGMNREECVPLRYASV
LLSKGFVCSPHEVGVIVETHLRVVNAIDRLLISTPHPEEFLWVLWELCGLSRSDCGRQALL
VLGYFPEAISILIEALHSVKESEPVASGASPINLAIFHSAAEIFEVIVTDSTASSLDSWIGHAM
ELHKALHSSSPGSNRKDTPTRLLEWFDAGVVYHKNGAIGLLRYSAVLASGGDAHLTSTSIL
VADLTDVEQVVGDALGGSDINVMDNLGKLISDKSFEDNPLRDSSITQMTTAIRILAFVSEN
STVAAALYDEGALIVIYAILIKCSLMLERSSNSYDYLVDEGTERNSTSDLLLERNREQSLVD
LLVPTLVLLINLLQKLQEAKEQHRNTKLMNALLRLHREVSPKLAASAADLSSPYPDSALG
FGAVCHLVVSALTCWPLYGWTPGLFHSLLANVQATSLLALGPKETCSLLCLLNDLFPEEG
VWLWKNGMPMLSALRKLAVGTLLGPQKEKQVDWYLETSHREKLLNQLTPHLDKIAQIIE
HYAISALVVIQDMLRVFIIRIACQKIEYASLLLQPILCCIRNHLSDLTSPSEIDAYKVYRYLDF
LASILEHPCAKELLLEEGIAEMLTQVLERCLVAIGSDGKQISDSKISAKSGFTLISWCCPVFK
SFSLLCVPRTPLPYPVRHDLHSSASLSAKDCSLILPYLLKSCQVLPVGKELLSCLAFFKDLG
SCNEGQSACVTTLHHINTSIEEHESGKGQERNGNYNLDDIEWRKHPPLLSCWIRLLESVDS
KDDASICALEAVTTLSIGALCFCLDSKCNLNLNGVAAIKKLFGIHDDMDGTDSSPENIGFIL
EMITLLSSKLNDDDYLATDMRESLYQASDSAKSLLLLLQKPTGSVTIDDIMSSEGIQSLPSN
ELLVHSRINQMADGTAEKFDGYLYLGGLGDKFLWECPETLPDRLSQNPSMKRKLASLDGS
GKRVKGETSVAEATVQNAFSRGMGSSTAPSGPTRRDTFRQRKPNTSRPPSMHVDDYVARE
RSVDGVSNSNVIAVQRVGSTGGRPPSIHVDEFMARQRERQNPMVAVVGEPSAKVKNATPA
NDVDKEKDNKSKQLKTVLDDDLQGIDIVFDGEESESDDKLPFPQPDDNLEQLAPVIGDQS

SPHSIVEETESDVNGNNQFSHSHTPLASHVDENTQSEFSSRMSVSRPEMPLTREPSVSSDKK
FFEQPDDAKNTIKTSAGFDSISAASTSGFPHQIPVDSRMPPQNFYMKNSLQHSSGSRGLYD
SKIPLNQPPLPPMPPPAMSSMIPQNHDPGPTQSSPYVNSGTEVQPPLPAAFQVQSDYLSAFG
SNPSIQMPDSKYSRASISSPSGSAGPHPPLPPTPPPFSSSPYNLPSLNPSTSQSSVYTVGTNEL
PQTSTSPPIDPRLGNLSVSGAGLTSYMPPPLMPPMVFSRPATIPVTPYGSIPTQQQGESPNVL
QNLSIPQPSVQSIHQLQPLQPPLRRPPQPPQHLWSLAQSSQQLEQGGSLQSSIQMQGHQLQ
MLQQQQLPSVHAHYQAQQQELSQSRQQLVEHAQPHVIHQQGDVSSQQQQDLGMSLQEY
FKDPKAITSLLSNKEELCRLLEQNPKLMQMLQERLGQQ
>V.v201s0026g00760.1
MGRPEPCVLFAQTFVHPQLDEYVDEVIFAEPVVITSCEFLEQNASSVSPVITLLGATSPPSFA
LEVFVQSEGETRFRRLCQPFLYSHSSSNVLEVEAVVTNHLVVRGSYRSLSLVIYGNTAEDL
GQYNIEFDLDSSLTNVVCSSEGKLDDLPPALHSKNLTIEESISSLKALSLPVAASDISIEIKQF
LQLMFKILELTNLGDAVHKVLDTVVSAASSYSAHDLHYAAVNQKKFTQSTNNSNEESHFV
LDAAKKELLDLYKTLQDESGNSSVELLEECSFLESEIDLASSKELMDMLIQHFLFKRNFLS
VGHYHLSQKKKVILVLSVALFLCSAKESCFQFVNGGGMEQLACVFSDDLQNSTAITLMLL
GVVEQATRYSIGCEGFLGWWPREDDNVPSGISEGYSRLLKLLLEKQRHDIASLATYALHRL
RFYEVVSRYECAVLSVLGGLSTVGRVTGATLDMLISAKVQLKKLLKLINSRGPIEDPSPVA
CASRSLILGQTEGLLSYKATSNLIGLSNCCFSSRDIDIHLLSLVKERGFLPLSAALLSSSILRS
EVGHAMDIFVDITSSIEAIILSLLFCRSGLIFLLLHPELSATVILALRGVDDFHKEDCAPLRYA
SILISKGFFCRPREVGLVVEMHLRVVNAVDRLLSSTPQSEEFLWVLWELCGLSRSDSGRQA
LLALGHFPEAVLVLMEALHSVKELEPVTTTGTSPLNLAIFHSASEIFEVLVTDSTASSLASWI
GHAMELHKALHSSSPGSNRKDAPTRLLEWIDAGVVFHKNGVTGLLRYAAVLASGGDAHL
TSTSILGSDSMDVENAVGDSSSGSDTNVIENLGKLISEKSFDGVTLRDSSVAQLTTAFRILAF
ISENSAVAAALYDEGAIIIIYAVLVDCRFMLERSSNNYDYLVDEGTECNSTSDLLLERSREKS
LVDLLIPLLVLLITLLKKLQEAQEQHRNTKLMNALLRLHREVSPKLAACAADLSSSYPDAA
LGFGAVCNLLVSALACWPIYGWTPGLFHSLLASVQATSSLALGPKETCSLLCILNDLFPEE
GVWLWKNGMPLLSAVRTLAVGTLLGPQKEREVNWYLHPGHPEVLLNQLTPQLDKISQVI
LHYAMTSLVVIQDMLRVFIIRIACQKADNASLLLQPIMSWIRMRLSESSCQTDVDAYKIYR
LLDFLACLLEHPCAKPLLLKEGAIQMLIKALERCVDATESDGKQLSDGRNSAKCSLTAFSW
CLPLCKSLSLICGSHMSRHYIGNYAKNDFEHLSSEDCSLILPYLLKLCQILPVGRELLACLT
VFKELGSCNEGQNALMAVFLRARSSDEELELEKGHERGGNYNVLNEYEWMKLPPLLCC
WTKLLRSVDPSDGFPAYAIEAVGALSLGALRFCMDGKSLNLDRVFAMKFLFGLPHDLSGM
DDFPEENIRYIQELTTLLGSKVTDEDYSAKSDMKTTLCRASDYAKSLLLMLQNPAGSLDLG
DIISSEDVPLSPNDVILSSRIHQMIDNSAEKVEDYCCLGGLEDKFLWECPETLPDRLLQTTLP
AKRKMSSLEGPSRRARGDNSPAETVAQGAFSRALGPPSASSGPSRRDTFRLRKPNTSRPPS
MHVDDYVARERNVDGVSNSNVIAVQRIGTTGGRPPSIHVDEFMARQRERQNPVVSAVGE
VAAQAKNAAPENDADMEKFNKSRQIKADLDDDLQGIDIVFDGEESEPDEKLPFPQPDDNL
QQPASVIVEQSSPRSIVEETESDVNENSQFSRLGTPLALNVNENPESEFSSRMSVSRPERPLT
REPSVSSEKKYFEQSDDMKNVIPAMTPSRYDSAGAAISSGFPASTYGKASVSSVPLMVDSR
MVQPNFYLKNSSQQAGNMALATGSQGLYDQKFMLNQPPLPPMPPPPTISPIISQAPDPALS
QSSSFVNTATDVQPPLPTAFQVQSEYLSAFTNSSTSLASSLSMPDSKYSRASLSSPSGSARPP
PPLPPTPPPFSAAPFTLASLKVSVSSSSVYNQTSGATTDLPQISGASLTDARLGNLSASGTRL
SSYPPPLVPPLVFSRPASIPVSIYGSTTTQQQGENPSNTIQNPPIPQLSIQSIQSFAQLQPLQPPQ
LPRPPQPPQHLRPPVQPSQQPEQGVSLLQSPIQLPVQPLQMLQQPQVSPLHVYYQQQQQE
NFPHVQQQQQVEHGQHQVLRQQGDSSSQLEQDSGMSLQQYFSSPEAIQSLLCDRDKLCQ
LLEQHPKLMQMLQERLGQL
>Phvul.007G267500.1
MGRPEPCVLFSQTFVHPHLDEYVDEVIFSEPIVITACEFLEQSASSVAQAVSLVGATSPPSFAI
EVFVHCEGETRFRRLCQPFLYSQSSSNVLEVEAVVTSHLVVRGSYRSLSLVIYGNTAEDLG
QFNIDIDDNALTDLVDSTEGKLEDLPPALHSTNFTIRDSRSSLSVLSIPVPATNIALEVNLFLQ
LMLKFLEFSDPGDAGHKIVNSVVSAISSYISSDICESISGRYQMWKRSENLEELHGAINEAR
KELLEVYKVLHRKSRSDSSECSSEANYLEMDVEMLDSKTLVDMFNQYFNFQIHSSCTGD
HCLSQREHALLGLSMAYLLCSGRESGFQFVSSGGMEQLAVFFSKDGQNSTTIMLLLLGVIE
RATRYSVGCEAFLGWWPREDESIPSGISEGYSYLVKLILSKPRHDVASLATYLLHRLRFYEI
ASRYESAVLSVLENISTVGRVTDVTLNMLSSAEILLRKLLNLINSRGPIEDPSPIARASRSLIT
GQTDGLLSYKTTSSLISSSSCCFSDCDIDSHLLGLLKERGFLSLSTALLSSSILRTGTGHVME
LFMDVTSSVEAVILSFLFSRSGLIFLLQDPELSSTLILALRGGHRGNKENCIPLQYASILISKG
FFCSPLEIGMIIEMHLKMANATDSLLSSNPQSEEFLWVVWELSTLSRSDCGRRALLALGNF
PEAVSILIEALSSIKESESVGKNSGSSAVNLTIFHSAAEIIEAIVTDSASSSLGSWIGHAMELH
RALHFSSPGSNRKDAPSRLLEWIDAGVVYHKHGGIGLMRYAAVLASGGDAQLTSTSILVS
DLTDVENVVGESSSGSDINVMENLGKFISEKSFDGVTLRDSSLAQLTTALRILSFISENPTVA
ATLYNEGAVIVIYAILVNCRFMLERSSNNYDYLVDEGTECNTTSDLLLERNRELNIVDLLVP
SLVLLITLLQKLQEAKEQHRNTKLMNALLRLHREISPKLAACAADLSSRYPDYAIGYGAVC
HLIASALAFWPVHGWSPGLFNTLLASVQSSSLLTLGPKETCSLLYLLSDLFPEEDIWLWTS
GMPLLTTRRMLGIGTILGPQKERHVNWYLESGHLEKLLGQLVPHLDKIAEIIQNYAISALG
VVQDLLRVFVIRISCQNPKYASILIKPVLSSIVHLASESSFPSDTDAYKILRLLDFLVSLLEHP
LGKVLLLREGTLQILTKLLDRCFVITDDGKQTPDRSSATCSFNIYSWCLPIFKFIMLLFHSET
SHHYPRRHDFKNFEKLSDEDSALILQYILKSCQVLPVGKELLACLTAFKDLASCDEGQMA
FGATHLGINSHAYELDPRKGDRNVNYSVSSVAEWRKCPPLLSCWMKLLKSIDDTKEGLST

CAIEAVYALSVGSIQFCMNGDSLNSDRVVALKYLFGISDDMTRSVGFPEENINYILEFSALL
SSKAAMDDCLVTSFSQIPLYQVSESVKSLSLILERPAGSMKLEDAVLPQYDVLGFSNRHQL
LENSVEKIDDHLYVGGLGDKFLWECPEILPDRLTQTNLAAKRKLPSMDGPVRRARGESFQ
GDISSQNAFSRGPAQSAVSSGTTRRDAFRHRKPNTSRPPSMHVDDYVARERIVEGVTNVIS
VPRAGSTGGRPPSIHVDEFMARQRERQNPSATVVGEAVGHLKNASPVKPADMEKLNKSK
QLKTDLDDDLQGIDIVFDGEESDPDDKLLFPQLDDNIQQPAPVIVEQSSPHSIVEETGSDVV
DSGQFSQMGTPLRSNVDENAQSEFSSKISGSRPDMSLTRESSVSSDRKYVEQADDLKNVQ
VKPSGRYDSAASNTSFPMSLYNNPSSSMQLPADSRMVSQNYLLKNSPQHGGIATGSQGLY
DQRFLPNQPPLPPMPPPPTVSPIISHATDSVPSQSTSFVNPQAGTQRPVAFQVQLDYPSPFNN
GTTATALASSIPMQDSKYSRTSVSSPGGPNRVAPPLPPTPPPFVSSQYNLSSVKSSGSQPSIY
NQTSMGTTELSHSSIASSGARLSSYPNPPMGFSRPASMPLSMFGNAPNQQQTENQPNILQN
ISVPPASFQSMHSVTQLQPLQPPQLTRPPQPPQLRPPVQALQQLEQGMAVQSNAQVHQINM
LQQSQVPSMQTYYQTQQQQFSHEQLQPHVEYTQQPADGQSQQQPDAGLSLHEYFKSPEA
IQSLLRDRDKLCQLLEQHPKLMQMLQERLGQL
>Glyma.02G195600.1
MGRPEPCVLFAQNFVHPHLDEYVDEVMFSEPIVITACEFLEQSASSVAQAVTLVGATSPPSF
AIEVFVHCEGETRFRRLCQPFLYSHSSSNVLEVEAVVTSHLVVRGSYRSLSLVIYGNTAEDL
GQFNIDIDDNALTDLVDSTEGKLEDLPPALRSTSFTIDDSRSYLNVLSIPVPATDISVEVNLFL
GLMLKFLEFSDLGDAGHKIVNTVVSAISSYISSDICESIGGRYQMRKRSENLEELHIVVDEA
RKELLEVYKVLHKKFRSESSECSSDAYYLEMDAEMLDSKTLVDMFNQYFHFQRNSSCIGD
HCLSQSEHALLGLSMAYLLCSGRKSGFQFVSSGGMEQLALFFSKDGQNSTTIMLLLLGVV
ERATRYSVGCEAFLGWWPREDDSIPSSISEGYSHLLKLILSKPRHDVASLATYLLHRLRFYE
IASRYESAVLSVLGNISTVGRVTDVTLNMLSSSEILLRKLLKLINSRGPIEDPSPIACASRSLI
TGQTDGLLSYKTTSSLISSSSCCFSDCDIDSHLLGLLKERGFLSLSTALLSSSKLRMESGHA
MEIFMDVTSSIEAVILSFLFCRSGLIFLLQDPELSSTLIHALRSGHRGNKEDCIPLRYASILISK
GFFCSPLEIGMIIEMHLKMVNAIDSLLSSNPQSEEFLWVVWELSTLSRSDCGRQALLALGN
FPEAVSILIEALSSFKESESVGKNSGSSAVNLTIFHSAAEIIEAIVTDSTASSLGSWIGHALELH
RALHFSSPGSNRKDAPSRLLEWIDAGVVYHKQGGIGLLRYAAVLASGGDAQLTTVLVSDL
TDVENVVGESSSGSDINVMENLGKFISEKSFDGVTLRDSSLAQLTTALRILSFISENPTVAAT
LYDEGAVIVIYAILVNCRFMLERSSNNYDYLVDEGTECNATSDLLLERNRELNIVDLLVPSL
VLLITLLQKLQEAKEQHRNTKLMNALLRLHSEISPKLAACADDLSSPYPDYAIGYGAVCHL
VASALAFWPVHGWSPGLFHTLLASVQSTSLLTLGPKETCSLLYLLIDLFPEEDIWLWTSGM
PLLTARRMLAVGNILGPQKERHVNWYLESGHQEKLVGQLAPHLDKIAEIILHYAVSALVVI
QDLLRVFVIRIACQNAKYASMLIKPALSSVIHHVSESSCPSDTDAYKVLRLLDFLVSLLEHP
LGKGLLLREGTLQILTKVLDRCFVIVDVDGKQIHDRSSAKCSFNFFSWCLPIFNFMMLLFR
SEISRHYPRRDDFKNFEKLSDEDCALILRYLLKSCQVLPVGKELLACLTAFKELASCGEGQ
MAFGATHFGIHSHALELEPRKDDRNVNYNVSSVAEWIKCPPLLSCWMKLFRSIDTKEGLS
AYAIEAAYALSVGSLQFCMDGDSLNSDRVVALKYLFGISNDMTRSDGFPEENINYILEFSAL
LSSKASMDDCLVNSQSQIPLYQVSESVKSLSLVLQRPVDSMKLEDVVLHQNEVLVFSKTH
QLLENSVEKIDDHLNVGGLGDKFLWECPETLPDRLTQTTLAAKRKLPSMDGPVRRARGES
FQADMSSQNAFSRGVAQSAVSSGPTRRDAFRQRKPNTSRPPSMHVDDYVAREKNVEGVT
NVISVPRAGSTGGRPPSIHVDEFMARQRERHNPSATVVGEAVGHPKDASPVKPTDTEKLN
KSKQLKTDLYDDLQGIDIVFDGEESDPDDKLPFPQLDDDLQQPAPVIIEQSSPHSIVEETESD
VVDSSQFSQMGTPLGSNIDENGQTEFSSKMSGSRPDMSLTRESSVSSDRKYVEQADDTKN
VQARPSGRYDSVSSNTSFPMSLYNNPSTSMQSPADSRMVSQNYLLKNSPQHAGIASGSQG
LYDQRFLTNQPPLPPMPPPPTVSPVISHATDSVPGHSSPFVNSLAGTQRPVAFQVRSDYSSPF
INGSTAASSVPVPDSKYSRTSVSSPGGPSRVAPPLPPTPPPFASNQYNLPSVKTSASQPSMYN
QTSIGATELSQASISSSGARLSSYPNPPMMSAGFSRSASMPLTMFGNSPNQQQTENQPSILQ
SISVPPASFQSMHPVTQLQPLQPPQLPRPPQPPQLLRPPVHALQQLEQGMAVQSNVQVHHQ
LQMLQQPQVPSMQTYYQTQQQQFSHEQQQVEYTQQPGNSLSQQQQDAAMSLHEYFKSP
EAIQSLLSDRDKLCQLLEQHPKLMQMLQEKLGQL
>Glyma.10G082100.1
MGRPEPCVLFAQNFVHTHLDEYVDEVMFSEPIVITACEFLEQTASSAAQAVTLVGATSPPSF
AIEVFVHCEGETRFRRLCQPFLYSHSSSNVLEVEAVVTSHLVVRGSYRSLSLVIYGNTAEDL
GQFNIDIDDNALTDLVDSTEGKLEDLPPALRSTNFTIDDSRSSLRVLSIPVPATDISVEVNLFL
QLMLKILEFSELGDAGHKIVDPVVSAITSYISSDICESIGGRYQMQKRSENLEELHSVVNEG
RKELLEVYKVLHKKFRSGSSECSPDANYLEMDAEMLDSKTLVDMFNQYFHFQRHSSCIG
DHCLSQSEHALLILSMAYLLCSGRESGFQFVSSGGMEQLAVFFSKDWQNSTTIMLLLLGV
VERATRYSVGCEAFLGWWPREDENIPSSISEGYSHLLKLILSKPRHDVASLATYLLHRLRFY
EIASRYESAVLSVLGNIGTVGRVTDVTLNMLSSAEILLRKLLKLINSRGPIEDPSPIACASRS
LITGQTDGLLSYKTTSSLISSSSCCFSDCDIDSHLLGLLKERGFLSLSTALLSSSILRVESGHV
MEIFMDVTSSIEAVILSFLFCRSGLILLLQDPELSSTLIRALRGGHRGNKEDCIPLRYASIFISK
GFFCSPPEIGMIIEIHLKMVNAVDSLLSLNPQSEEFLWVVWELSMLSRSDCGRQALLALGN
FPEAVSFLIEALSSIKESESVGKSSGSSAVNLTIFHSAAEIIEAIVTDSTASSLGSWIGHALELH
RALNFSSPGSNRKDAPSRLLEWIDAGVVFHKQGGIGLLRYAAVLASGGDAQLTSVLVSDLT
DVETVVGESSSCSDINVMENLGKFISEKSFDGVTLRDSSLAQLTTALRILSFISENPTVAATL
YDEGAVIVIYAVLVNCRFMLERSSNNYDYLVDEGTECNATSDLLLERNRELNIVDLLVPSL
VLLITLLKKLQEAKEQHRNTKLMNALLRLHREISPKLAACADDFSSPYPDYAIGYGAVCHL
VASALAFWPEHGWSPGLFHTLLASVQSTSLLTLGPKETCSLLYLLIDLLPEEDIWLWTSGM

PLLTARRMLAVGNILGPQKEKHINWYLESGHQEKLVGQLAPHLDKIAEIIQHYAVSALVVI
QDLLCVFVIRIACHNAKYASMLIEPVLSSVVHHVSESSCPSDTDAYKVLRLLDFLASLLEH
PLGKGLLLREGTLQMLTKVLDRCFVIVDVDGKQIHDRSSAKCSFNFFSWCLPIFKFIMLLF
HSETSRHYPRRHDFKNFEKLSDEDCALILRYLLKSCQVLPVGKELLACLTAFKELASCGEG
QMAFGATHFGIHSHALELEPRKDDRNVNYVSSVAEWIKCPPLLSCWMKLLRSIDTKEGLS
TYAIEAAYALSVGSLQFCMNGDSLNSDRVVALKYLFGISDDMTRSVVFPEENINYIQEFSAL
LSSKASMDDCLVTSHSQIPLYQVSESVKSLSLVLERPVDSMKLEDVVLHQNEVLVFSKTHQ
LLENSVEKIDDHLYVGGLGDKFLWECPETLPDRLTQTNLAAKRKLPSMDGPVRRARGESF
QADMSSQNVFSRGVAQSAVSSGPTRRDAFRQRKPNTSRPPSMHVDDYVARERNVEGVTN
VISVPRAGSTGGRPPSIHVDEFMARQRERQNPSATVVGEAVGHLKNASPVKPTDTEKLNK
SKQLKTDLDDDLQGIDIVFDGEGSDPDDKLPFPQLDDNLQQPAPAIVEQSSPHSIVEETESD
VVDSSQFSQMGTPLGSNIDENAQSEFSSKMSGSRPDMSLTRESSVSSDRKSAEHLDDSKN
VQARPSGRYDSVASNTSFPMSLYNNPSASMQSPADSRMVSQNYLLKTSPQHGGIASGSQG
LYDQRFMPNQPPLPPMPPPPTVLPVISHASDSVPGHSSPYVNSPAGTQRPVAFQVQLDYSSP
FNNGSTAASSVPVPDSKYSRTSVSSPGGPNRIAPPLPPTPPPFASSQYNLPIVKASASQPSMY
NQTSIGATELSQASIASSGARLSSYPNPSMMSVGFSRPASMPLTMFGNSLNQQQTENQPSM
LQSVSVPPSSFQSMHSVSQLQPPQLPRPPQPPQLLRPTVQALQQLEQGMGLQSNVQVHQL
QMLQQSQVPSMQTNYQTQQQQVEYTQQPGNCQSQQQQDAAMSLHEYFKSPEAIQSLLS
DRDKLCQLLEQHPKLMQMLQERLGQL
>Aqcoe1G078100.1
MGRPEPCVLFAQTFVHPQLDEYVDEVLFGESIVITACEFLEQNASSASSVVTLVGATSPPSF
ALEVFVQCEGEPRFRRLCQPFLYSHSSSNMLEVEAVVTNHLVVRGSYRSLTLVVYGNTAE
DLGQFNIEFDLDSSLANLVCSPSEGKLEDLPPALHSTKFSLEESLSIPKLLLLPVAEPDLSFEK
EHFLQLIIKCFGASGNDDAMHKVASMVISAVSSYVTSDLGCTAITWNQCKQADLISCRKEL
QSILNDAKNELNELHKILQHEVSSLPVDLLEEGVAVKSEAELATALAEHLPDVFSWYHLFK
RICPSNGRTLSQNKSMILGLSMVLFLCSGRETCFHFVNSGGMEQLVRVFHHETQKSAAVTL
TLLGVIERATRYAIGCEGYLGWWPREDEIVPVGVSEGYSQILKLLLQKQQHGIASVAAYLL
HRLRFYEVAARYESAVLSVLAGLSDVGEVTEGTLNMLDSAKSQLKNLSKMLNSRGPIEDP
SPVANAHRSLVLGQTDGLLSYRSTKKLIASSNCCFSDFDIDSHLLSLLKERGFLPLSAALLS
SSNLRSEKGHTLDIFLDIASLVEAILLSLLFCRSGLVFLLLQPEVAAALVHSLKGVEDMNKE
ECVPLRYASVLINKGFFCRSQDVGMITELHLRVVNAVDRLLASTPQSEELLWVLWELCGLS
RSDSGWQALLVLGHFPEAVSVLMEALQSAKELEPTSLNSGSSPLNLAIFHSAAEIFEVIVSD
STASSLGSWIEHSVELHKALHSSSPGSNRKDAPTRLLEWIDAGVVYQRNGAIGLLRYAAVL
ASGGDAHLTSTSILVSDSMDVENVVGDSASGSDIQVIDNLLGKLVSDKYFEGVTLRDSSIA
QLTTTFRILSFISENSSVAAALYDEGAVTLIYVILVNCKYMLERSSNTYDYLVDEGAECNSM
TDLLLERGREQSLVDLMIPSLVLLITLLHKLQEAKEQHRNAKLLNALLRLHREVSPKLAAC
AADLSSPYPGSALGLGAVCHLLVSALACWPVFGWTPDLFHCLLDSVQATSSLALGPKEAC
SLLCLLGDLFPEEGIWIWKNGMPSLSALKKLSIATLLGTQKEGHIDWYLQPEHVATLLSCL
TPLLDKIAQIILHFASTALVVIQDMLRVFIIRIAYQKPASAVILLRPIISWIHDHVSEPYSLSDT
DVFKVYRLLDFLASLLEHPYAKPLLVKEGAVGILVKALGKCSSSFGLEGKVIAESRMRGSG
FTLINWILPVLKSVILFCGSQSSLQQSDVLDSCGNLSVQDCSLILHHLLSLCQVLPVGKELH
ASLIAFKELAYCSEGRSAFASISTHLQSPNLDEFQLEGGHEEDGYDGVTNNSDWRRCPPLL
YCWRNLLRSISGRECLSTYAIEAVGALSLGALCLCLEGKSLNLERIAVLKVLFGVPIDLDDD
EQCPEESLKDFSELIGMLDTRVIDARHMSTSDMRATLSQVKELAKLLLLLLQKPTTSIKVD
DIICNGSFSLLSSDILDFPFTSVTTMSILDEDAGSLNSRIRKPDGSSERDEDFFSFSGLADKFL
WDCPDSLRDSMSALPSKRKMASMELPNRRSRVDNSGTESTGQNAFARGVGPPTVSTGPT
RRDLFRQRKPNTSRPPSMHVDDYVARERNIDGASSGSNVVNSVQRGVTGGRPPSIHVDEF
MARQRERQGSVAMAGGEAAVPTRNVPPENENDPDKVDRSRQLKADLDDDLQEINIVFDD
EESESDDRLPFPQPDDNLQPAPVVVDGSPPHSIVEETESDANGSTHVSDMGTPLTSNVDEN
TQSEFSSRRSISRPEVRLSREASISSERKYFSSNTDRPFFRSKSDDAKHSGSVRASNGFDSAT
ANFSGFPPPFYKGSPSSGQLVGDSRMSPSNFYQRDSPQHAPNIPSSSQGLYNQKFVPNQPPL
PPMPPPPNVSCVLSHSTENAQSHSSSYGHNIRDRQPPLPPGFPSQAFEGGGSITAPVYNVRE
DRSTSHNYAAGSNPPSSSSSYVESLNDPSALQLQTDYYTSSAALLASQQGMFDPKYSWTS
VSSASRSHDEINSSSSGLVRPPPLPPTPPPFSASLTAQSSVKNSASQSPGYNQTNAGGNLTSY
SPPQLVPPLLNSRPASIPVSLFSSPTIHQGQNLPGLSHSISTTQPSILSVQPRPQLQPLQPPQPP
HPHPPHLRPPNQGSQPEVALLQSPIQVQSHPFQMHQQSHLSPIQVYYPPQQLENLAHTQQL
QQVERSQPQALQQQGGNTPQQQQDAEMSLQQYFASPEAIQSLLSDREKLCQLLEQNPKL
MQMLQERLGQL
>Spov3.S03.00008
MRIKKIKINKKLFFKKIKRKTLLKSTDFPVINSHGSLSFQERFPPLLLTAFAANTPARWLCGD
ACYHTARRLSEYLNMGRPEPCVLFAQTFVHPQLDEYVDEVMFAEPIVISACEFLEQNAPSA
SSAVTLLGATSPPSFALEVFVQCEGEARFRRLCQPFLYSHSSSNVLEVEAVVTNHLVVRGSY
RSLSLVVYGNTAEDLGQFNVEFDLDNSVAKLVSSSEAKLEDLPPLLHSRNPKFEDAISSLK
ALSSMLPAADISVQMRQFLQLIFKILDIPNFGEFSQKVMALITSAVASFFTDDLASAGITLQE
LNLGALANVKGSFHVFTEAQRNLFELCKRYVGAGHIYGDFLVGCSFLESEAELATSKELV
DMLFQHFPFNRDTVFVGHPHLSKSNNVLLWLSLALILCSGKESCFHYVNGGGMEQLAHF
FSQEMLNSATVTLMLLGAMEQATRYSVGCEGLLGWWPREGEKVPSGVSEGYSLLLKLL
MQKQRHDIAALTTYILHRLRFYEVASRYEYAVLSILGGLSGDGNATTTTLSLLLKAKLQLR
KLLKMINSSGWVEDPSPMACAIRSLMVASSEGSLSFKATSKLIASSSCRFLHQKIDAKLLSL

LKERGFLPLSVALISSTELRAERGHALEAFLDLTSSIEATILSLMFCRSGLVFLLQHPDLSSTII
RAFKGDDNVTKFIPLRYAYVLVSKGFFCHLQEVGTNLEMHLRVVHAVDRLLTSTPCSEELL
WVLWELCGLSRSDGGRQALLALTHFPEAFTILIEALHSAKELEPISLSCGTPLNLAIFHAAA
EICEIIVTDPTSSSVSVWIDHATDLHRALHSSSPGSNRKDAPSRLLEWIDAGVVYHKNGAV
GLLRYAAVLAFGGDAHITSTNILVSDSMDVEDVVGDTSNESDVNVVESLLGKLVSDKSFD
GVALRDSSVAQLITAIRILALISENADVAASLFDEGAMTVVYIILVNTRLMLERSSNNYDYL
VDDGIECSSTSDLLLERNREKYLVDLLIPSLLLLIELLQRLKEAKEQHRNTKLMNVLLRLH
RELSPKLAACSVDLSSSYPDSALGLETVCHLIVSALACWPAHAWAPGLFHTLLDSVQAAS
LLALGPKETCSMLYLLIDLFPDEGVWHWKNGMPLLTASRSLAVGTLLGPLEERKVNWYL
ERPCVEILLGQLSPHLDKIAQVIHHYAVGTLVVVQDILRILILRIARQRTEFATVLLQPMISWI
ENHLIGTSPLDEIDAYKGYKLLDFLSLLLEHPLAKTLLLKEGTLQILTKVLGRIDAHVKSVS
NGRSSLNSRFNIYNCCLPVLRSLSLICETRSAQKLHLLLDGNETLSTEDSAVIFSYIFKLCQH
LPAGNELVACLGTFKDLASTAIGQSTLLDTYLFIRSSSNNENQPGRSQESYQFYNRLIDAEW
KGCPLLTCWINLYGSVETEGLSTCSVEAIGLLSIGVLRFCMSGRRYPKKDLVKLVPYDLSF
NEDRIDALKYFFGLSSDVDGANGVLEENIKYVQDMCTLLSSKITDDEYAVSSHLKATLHQ
AVESVKFLSLLLLKPIDSFKDDDAFTDEVLLFSSDAKLSSKIHLLTDSSSERVENELNFGELG
ESFQWECPENLRNRLSQAGLLGKRKISAVDGVNRHGKGENAPVDVRGSGSSMAPPIPTRR
DTFRQRKPNTSRPPSMHVDDYVARERNESSNPNVIAVPRLGSSGGRPPSIHVDEFMARQRE
RHNSVPIAGVEASSQGKKITLEIDNDTEKSNRSKQLKVDLDDDLQEIMFDAEENEADDKL
PFPQADDDVQQTASASAETSPQHSIVEETESEAKDLEATVASNAVENAQSELSSRMSVSRP
EMQLRREPSVSSENKYFEQPDETNNKNPTMVSGGFDSVAVAKTSGFPASVYGKHSPSGAQ
VPADARIPPSSFIPDNNLQRVGNVLLGRSQGRHEQKHLPNQPPLPPTPPPSTISPVRSQVSEN
QTSRSPFVNPHNDVQPPLYPTYAQTDYQSSFGNVPTSFASSNFMSDSRYARTTNSSPGSSNR
PLPPLPPTPPSFSATPLNVPSRSPTSQSSMYVQHNAGATDASQYSSPPLIPPGFSRLPSMPFN
MHGNIPVQQSDNITGIAQNSRGPQPSMQSMQPLPHLQPLQPPQLPCPQQLRLPIQNSLHTE
QSGSMSQSHVQMQVQPPQILQQSQTSPYHLYYQTQSQENIPHPQQPQAAHHQVDAATQQ
QMDSSMSLQQYFSSPEAIQSLLSDRDKLCQLLEQHPKLMQMLQDQLGHL
>Solyc03g020020.3.1
MGRPEPHVIYSHTFNHPQLDEYVDEVLFAEPVVVSSCEIVEQNAPSACSSLKIVGATSPPSF
ALEVFVHCEGETRFRRLCQPFLYSHSSSNVLEVEAIVTNHLVVRGSYRSLTLVVYGNTTED
LGQFNIDVDLDGSLANTVSVVEGDLEDLPPALRPNNLSTEQTLSSLKSLSLKSIPLAVPLEL
RQLLQLTLRMLESPEFGVMKNKVLTSLLSVASIYATPCFPSTTTMHEQLGLDKLVFNQEAQ
FAIAEAKKELLEMYNSFIFQPGDRSVEFSTDAMLVESEIEDAAPKQLLDSLSHYFKFASSPD
AASHREVSKRENMVLCLSLALLVSSARESCYHFVNSGGMEQLDYAFSSSLLNSSALKLLH
LGVIEQATRHSVGCEGFLGWWPREGENIPSGTSERYNQLLKLLLLHNQRHDVASLATYILH
RLRFYEVSSRYECSILSVLGGLSGSGQATSATLVDILTSAKNLLKNLLKLINSSGPIEDPSPVA
CASKSLVLGDSGQLLYNSTSNLITQSSCCFSNNDMDQHLLSLLKERGFLPLSAALLSSSAL
WSHAACTIDLFVDILSYFEAIVLSLLSTRSGLIFLGRDPEVATIIIHALRGADTWKKEESISLR
HASVLISKGYFCHPRDVALIIEMHLKAITAIDRLVTSSPDSEDLLWTVWQLCSLSRSDCGRK
ALLALVHFPEALSALIAILHSVKELDPVSPNSGAPPLNLAIFHSTAEILEVIVSDSSASSLGS
WIGHAKELHRVLHSSSPGSSKKDAPARLLDWIDASVVYHRSGAIGLLRYTAILASGGDAH
MASTSVLASDGMDVDNVIGDSSCTDGNIIENMLGKRITERDFPGVVLRDSSIVQLTTAFRIL
AFISDNSAVTAALYDEGAVMVIHAVLINCRLMLERSSNIYDYLVDEGTECNSTSDLLLERN
REQTLLDLLIPSLVLLINLLQKLKEAKEQHRNTKLLNALLQLHREVSPKLAACAADISYPY
PSFALGFQAACDLLVSALACWPVYGWTPGLFNFLLDSLHATSVLALGPKEICSLLCILNDL
FAEEGVWLWENGTPTLSVLRTLAVRTLLGPKKEKEINWFLQTGLREKLLGQLKPHLGKIA
QIILCCSTSTLVVIQDMLRVFIIRIACIGGDNASVLLRPMVLWIGDRLSEKLPPSDLDAYKIQ
RLLSFLSLLLEHAHGKRLFLKEGGLRMLIKALEMCLAAASSDAKQLAQKGFSLISWCVPV
FKSITLLSECKTRQTPGIVERHVPEDMTAEENCLLLSLLLKFCKVLPVGKELLSCLLVLRLF
WSSAKGKDALLSLYLHAKSSSIEEQESEKQFENGLNRDFSLDWKEHPPLLCCWESLLRTPA
SKDDLPTYAVQGIGILSSGALSFCMDGESVNTERVTAVKYFFGLENDNVAMDGLYEESIES
VEEFVNLLKASDSSFLPVLDKISLDQIKESARSLMLLLHKPTGTVKADDIMSNIHFPSPTYS
SKIHTIEDSGTERIEDYDLNEFGDKFSWECPENLRDSLTQTSLTNKRKISSMEGPNRRIRGD
SASTENAIPGAFSRGSVPTIVPSGPTRRDTFRQRKPNTSRPPSMHVDDYVARERSADGSNN
PNVIAVPRIGSTSGRPPSIHVDEFMARQRERQNPPGILVSDSAAAQEKAAIPEKQTDAEKSS
KSHPMKSDPDDDLQGIDIVFDAEESEPDDKLPFPQPDDNLHQPAPVVVEQNSPRSIVEETE
GEVNETSQFSQRGTPVASNADENAQSEFSSRMSVSRPDLPLAREPSITSDRKFNDQYEDMK
NFHPKTSTMFASPAAAVSSGVGASAFTKASSSIQVAVDSRMPPNFYSRPTGQQSGVTPPNIG
SQGYFDPKMQPPLPPTPPPVTMASLSQNADRILSQSSPFVSSMIDVQPHLPPGFHVQAEYL
SAGASTPMTSSPLPDSKFGRTSLSSPGGPVRPLPPLPPTPPPYTISLSNLSSLTNLTSQTPVYN
QSVGTNELQQTSNAHSSDVRSGNVSTSGPILTTYPPPPLAPPLLFNRHGSVPVSFYGSSSAP
YHNEKLPSISQHLPAIHSIPSVTQLQPLQPPQLPRPPQHVRPIVPASPQSEQSVPLLQSPMHM
QMQSPQILHQPQVSPAHVYYQTQQQENSLQQQQIEHSLSQVPQQQGDIVTQQQDSGMSL
QDFFRSPQAIQSLLSDRDKLCQLLEQHPKLMQLLQERLGHL
>AH018923.RA
MGRPEPCVLFAQTFVHPQLDEYVDEVIFAEPIVITACEFLEQNASSALSTVALLGATSPPSFA
LEVFVQCEGEARFRRLCQPFLYSHSSSNVLEVEAVVTNHLVVRGSYRSLSLVVYGNTAED
LGQFNVEFDLDNSVVNLVSSSEAKLEDLPPLLHSRSLKLEDSISSLKALSSVLPATDISLEMR
RLLQLAFKMLDIPNFGVFREKVLTIITSATASFFTNDLNSSGITLQELNWGAVPDSKEPFLVF

TKAKNDLLELCKSYVESGHICGDFLAECSFLESKTDLATAKELVEMFFQHFKFNKDIPYVA
HPHLSKSNNLLFWLSLSLILCSGKESCFHYVNGGGMEQLTHFLSHKVQNSTSVILMILGVV
ERATRYSVGCEGFLGWWPREGEDVPSNVSEGYSQLLKLLMQKQRHDIAALATYILHRLRF
YEVVSKYEYAVLSIVGGLSGDVHATTTTSSMLVNAKLQIRKLLKLIKSSGWVEDPSPVAGA
IQSLMVTSSEAVLSFKATSKLIASSSCRFLHQKIDAKLLALLKERGFLPLSVALMSSSELWSE
RGHALQAVLDVTSSIEATLLSLMFCRTGLVFLLQHPDLSSTIIHAFKGDDDVNDFIPLRYAS
VLISKGFFCRLRDIGINLEMHLRVQHHVPKSSSGYCGSFVVFLGTPLNLAIFHAAAEICEIIV
TDSASSSMSVWINHVTDLHKAVHSSSPGSSRKDAPSRLLEWIDAGVVYHKNGAVGLLRYA
TVLASGGDAHITSTNILVSDTMDVENVVGDSSSESDVNVVESLLGKLVTDKSFDGVTLRD
SSVAQLITAMRILAQISENADVAASLFDEGAMTVIYVILVNSRLMLERFSNNYDYLVDDGV
ECSSTSDLLLERNREKYLIDLLIPSLLLLLELLQRLKEAKEQHRNTKLMNVLLRLHRELSPK
LAACAVDLSSSYPDSALGLETVCHLIVSALACWPAYAWTPGLFHTLLDSVQAASLLTLGPK
ETCSMLCLLIDLFPDEGVWHWKNGMPLLTPARALAVGTLLGPLEERKVNWYLEHPYVEV
LLVQLSPHLDKIAQVVLHYAVSTMVVVQDMLRVLILRIARQRSEYAIVLLHPIISWIQNHLI
ENSPDEIDSYKGSKLLEFVSLLLEHPTAKPLLMKAGTLHILTKVLEGINSHVKPGSSIYNCC
VPALRSLSMICDSVSAHKPHRLLDGNWTLTTEDSAIILNNVFKLFQNLPVGNELLACLATF
KGLVTHAEGQRALLDAFQFIKSRNIWENQPGRMQDSHGFYGRLIDAEWKRCPLLCCWIK
LYQFIENEGLSTFSVEAVGELSSGALRFCIDGKSFNQDRIEALKYFFGLLCGIDEMSGIPEEN
MKYVQDMYTLLSSKIPDEECSTLTHLKTTLYQVVESARCLSLLLLQPIDSLKVDEIIPKEVV
LFSSDVKLSSKIRLLTDSSSKRVENEWNIGELGDKFLWECPENLRNRLSEAGLLGKRKMST
ADGSNRHGKGENSLVDTRSGSSVVPPIPTRRDTFRLRKPNTSRPPSMHVDDYVARERNAD
GVTNPNVIAVPRLGSSSGRAPSIHVDEFMARQRDRQSAVAMAGVEAVSQDKKVAPENNET
EKSKRSKQLKVDLDDDLQGIDIVFDAEENEPDDKLPFPQADDDVQQIASASVERSPQHSIV
EETESDAKDLEATVASNVDDNTQSELSSRMSVSRPAVPLRREPSVSSEKKIFDQTDEVRNK
NTVIMSAGSDSATVVKTSAVSTPTYVRGSSSVIHVQADSRTPHPGFMPDNNRPRAGNVPL
GRPQSHHEQKHFANQPPLPPTPPPSTISPVRSQVSEPNPASTSSFVNPHTDIQPPLYPNPAQT
DYQSFGNAPISTPNFIMDPRYVRTSHSSPGGSTRFFPPLPPTPPPFSAALLNVSSRSPTSQSSA
YAQNNTGVTEVSQYGPPPLIPAGFSRPASMPFNMHGNVPVQQNENMTGMAQNSHAPQPP
MQPMQPPHLQPLQAPQLPCPQLPQQLRPVMQSSQHTDHNVSISHNNVQPPQIMQQSQMS
PYHLYHQNQLQENSPHSQRQLQAAVPQTDAATQQQADSSMSLQQFFSSPEAIQSLLSDRD
KLCKLLEQHPKLMQMLQERLGHL
>Gomus.A04G072100.1
MNKEESVPLRYASVLISKGFTCNPQEVGIIVETHLRVVNAIDRLLSATPQSEEFLWVLWELC
GLARSDCGRQALLAMSFFSEVLSVLIEALHSVKESEPVIKNSGASPLNLAILHSAAEIVEVI
VTDSTATSLSSWIGHAMELHKALHSSSPGSNRKDAPTRLLEWIDAGLVYHKNGAVGLLRY
AAVLASGGDAHLTSTNILVSDLTDVVDNIVGESSNASDINVMENLGSIISMKSFEGVNLRDS
SIAQLTTAFRILAFISENPTVAAALYDEGAITVIYVVLVNCSYMLERSSNSYDYLVDEGTEC
NSTSDLLLERNREQCLVDLLIPSLVLLITLLQRLQEAKEQHKNTKLMTALLRLHREVSPKL
AACAADLSSPYPDSALGFEAVCHLSVSALAYWPVYGWSPGLFHTILASVQTTSSLALGPK
ETCSLLCLLNDLFPEESIWRWKNGMPLLSALRSLAIGTLLGPHKERQVDWYLECGHLEKL
FNQLTPHLDRIAQIIQHYAISALVVIQDMLRVFIIRIACQKAEQASKLLRPILSWIHDHSSDLS
SLSDTEAYKVYRCLDFLTSLLEHPYAKVLLVGEGFPQILTRVLESCFDATDSDGRQASDCR
DSAKYGVALISLCIPVFKSISLLCSSRTFSQYDERHEMHKFDSLSPKDCSIFINQLLKFCQVL
PVGKELVSCLTAFRDMGSCTEGCNALLSALLNSSSSTHDELESERGNEKNVNFHFLNESE
WRKSPPLLCCWIKLLKSIDSKDHLPPYTLEAANVLSLGTLGFCMGGNSLNMNSVVALKFL
FGLPDGTAGIGGFPEDNIKYIQEFSTLLSSRIDNDEDYQTSSDIHISMHQVSESVKSLLLLFQ
NLTAAVEVDDAILYGGLSFPQNNVQVPSGIQHFGQGLDGKADGSLYSGGFEDKFSWELPE
TLPGRLLQTALPTRRKLQAADSANRSARGDNSVAEITNPTAFQRGLGPSTASSGTTRRDSF
RQRKPNTSRPPSMHVDDYVARERSVDGVSNSNVIAVPRVGSSGGRPPSIHVDEFMARQRE
RQNPAASGTETATQSKNAAPINGPDNEKVNKSKQLKSDLDDDLQGIDIVFDGEESETDDK
LPFPQPDDNLQQLAPVIFEQSSPQSVVEETESDVNGSSQFSHMATPLASNADENAQSEFSS
RMSVSRPEMSLTREPSVSSEKKFFEQSDDSKNAVSIKNSSGFDSASGTNSSGFSAPIYSNTPA
TSVQLSLDSRITPQNFYPKSSAQYAGNIPVAAGSRGMYELKVLPNQPPLPPMPPPPTILPVQ
SDYLSSVSGSPSLLQSSIPVSDSKFMRTSMPSPSGTTRPPPPLPSTPPPFASSPYNLASLNTSA
SQPALYNQSGMGKTELPQGSIGPTIDARLPTSAAGLASYPPPPLMQSLVFNRPPSIPVTPYG
TSPALHQGENHPPGILQNPSIPQSSMQTIHSLNQLQKLQRPLLPTQHLRPSMQSSQQLEQVV
SSQTPVQMQIQSLPMMHQAHISPVNPYYQPQQSEFSAAQQQMQVELAQQQAPPQTGGTS
QQQDSGMSLHEYFQSPEAIQSLLRDREKLCQLLEQHPKLMQMLQEKLGQL
>Bradi1g41947.1
MGRPEPVVLFAQTILHSQLDEYVDEVLFSEPVVITACEFLEQNASPSTPNISLVGATSPPSFA
LEVFVHCDGESRFRRLCVPFLYSHSSSNVLEVEAIVTNHLVLRGTYRSLTLVIYGNTAEDLG
QFNIELDLDHSLANVVSSPSEGKFEDLPPALHSSKFKFEESLSSLKPLSFQSTDLDLSLEAKK
ILHLALKMCQIPIVEKLIPDLGSAVISAVSKYVTTTNRMPHSCNQEMAHGSSKINLDLQETN
NIHTEASDMLLQILKNVHSAAADHTHVDDNGFDFELEELPTTKILFELFNKIFPYYRDSVIL
DLQCPSQNSWLMMSLSLVLLICSSKESCFSFVNAGGMEQINNLLCSKTPKTAATTLLLLSIV
ENATRHVVGCEAFLGWWPRCDHSSIPTGSSSGYCSLLKLLMEKERHDIASLATYILQRLRF
YEILSRYESAVVKVVSDLPSDELSTDGVSLLIYASSELAEMLKLINMCGPIEDPLPVTAARKI
SKSAHMEDSLSFKATIELITSSKYSFLQFHTDSYLLSLIQERGFFPLSAALLSSPIMHLASAA
EISMEMASSIELIVLSLLFCRSGLSFLLSQPEATELIVLSLQDDKDMNKTECITLRHAFVLLS

KGFFCRPQEVGMITELHLKVGSAANRLLAVPPNSDELLWVLWELCAISRSDSGRQALLAL
CYFPEAISVLLSSLSSYTDLDSTVTKTGGSPLGLAIFHSAAEILEVLVADSTGSSLKSWIGFA
VDLHKALHSSSPGSNRKDAPTRLLEWIDAGVVYQRNGAVGLLRYSAILASGEDAHLSSGN
VLVSESMDVENVVADSNNTSDGQVIDNLLGKLVANKYFDGVALCSTSVVQLTTAFRILAFI
SEDTAAASSLFEEGAIAVIYIVLMNCKSMLERLSNSYDYLVDEGAELSSTTELLLDRTHEQ
ALVDLMTPSLVLLINLLHIIHGTKEQYRNKKLLTALLRLHREVSPRLAACASDLSFMFPSFA
VSFGVVCQLITSALACWPLYNWTPGLFHCLLENVEPTNASVPLGPKDACSLLCLLGDLFP
DEGVWLWTVEVPSLSAIRLLSIGTVLGPQVEKQVNWYLHPEHVALLLVRLMPQLDRLSRV
IDNFATSALMVIQDMLRIFIVRVASEKIECAVVLLRPIFTWLNSKVDETSLSEREVFKVHQLL
KFIAKLAEHPNGKELLWKMGVVSVLRKLLQNCSNASYLEDKMISDRGAYRNDLLVLKW
RIPLFRCLASIFSSQASNERQTAVEESPNGNTTAEECSSIMHQLLVLCQVLPVGREMLACSM
AFKEVASSSICRSAVPLIFSQIKTANQDDKEINESDTYHGSSNTDDWRCFSPLLKCLKRLLK
CIGANDPMDYYVETVYSLMLGAIALSQYGDSLEGIIVLRCLFGYRFDGGTLESSGDNLNEI
TVLLKTFEEKIHQGHENFLSSVGKSLLNQVQSYITLLCSILKNSVLSEDSVQMVLEGTYMP
FGVVRSVVMTSCLMPSLASESVNHESILFFSNAWKVIADSEEPTECLEDEFSKRLVWELPD
SSLERRMIPGQSASRKLSLGDNTTRRIKENQAPEPTGQFTRGLSTTNASTGHTRRDTFRHR
KPNTSRPPSMHVDDYVARERNIDGASSASNIVNSIPRGTLSGRPPSIHVDEFMARQKERQN
PVPAPSGDAPQLKSQTSLDDNVHAKSEKPRQPKAELDDDQEIDIIFDEESESDDKLPFPQPD
DSLQSPPVIVGENSPGPVVEETENQQNEESPFSHRGTPVSKDNGSLGAGMSSRTVMLPEAI
VPSERKLPLSSPEKTVFNDQPDEPAYVSSGSKRSAEAIVLQSHPNISQKRSAQKLSESSLSSG
SHGHDHRLSKNQPPLPPMPLPSSSMPVQNADSSQRRSSSYGVRDGPPSFPSSYPGQAFEAN
MPSDFVGLQAQTEHVLASNGGSSSNAPNADFNFLWNTFPVNRLPMEHFSSGSSARLMPPL
PPPYSVPATQIAAMNSGSTASPYNQRSSVVQPSPSSSLMSDATLGMNSASGGAILSNSLPSF
ASQFLIGRPSTCTSFFGTSPRQVQFSSGLSQNLSNPQPAVSSTHARPPPPPPLPQQPHPSQTL
QQLGSFQWQYQEQPLSYPQSSIQAQMPLQFPNQLSVPQMQYYQSQHESAQQTLRQGGEQ
SQLANQSIQADSLSQQQRDSEINLNQFFSSPEAIQSLLSDRDKLCQLLEQNPKLMQMLQDR
IGQL
>Sobic.001G191700.1
MGRPEPVVLFAQTILHSQLDEYVDEVLFSEPVVITACEFLEQNASPSTPNISLIGATSPPSFAL
EVFVHCDGESRFRRLCHPFLYSHSSSNVLEVEAIVTNHLVLRGTYRSLTLVIYGNTAEDLG
QFNIELGLDHSVANVVSSPSEGKLEDLPPALLSSKLSFEESLSSLKPLSFHATDVDLSIEAKK
VLHLALKMYQMSDVENLIPNLRSAVLSAISKYVTASTNHILHTSSQDSANSFTKSDFDSQEI
NNILAEAGNELSEIWKNVHAVTDSNLFNDNGFTIGGDEDLPTTKILIELFNQCFPYYKNFSL
LDLQCPSQNKWLVLSLSLVLLLCSSKESCFYFVDTGGMEQIINLLCWKTPKSAATTLLLLGI
VEHATRNGFGCEAFLGWWPQTEHSSIRVASSNGYCSLLKLLLEKERHDIASLATYVLQRLR
FYEILSKYESVVVKVISNLQADKVSTDGVPFLISASVELAEMLKLIICCGPIEDPSPVATARR
LFKSEHLEGLLSYKATIDLISSSKYSFLQYDTDPYLLSLIQERSFFPLSAALLSSPILHSASGP
AAEILMGIASSIESLILSLLFCRSGLSFLLSQPEATELIVLSLQDAENMNKAECITLRQAFVLL
SKGFFCRPKEVGMITELHLKVGSAANRILSVPPNSDELLWVLWELCAISRSDSGRQALLAL
GYFPEAISVLLRSLSSYKDLDSVMAKNGGSPLGLAIFHSAAEILEVLVADSTASSLRSWIGF
AVDLHKALHSSSPGSNRKDAPTRLLEWIDAGVVYQRNGARGLLRYSAILASGGDAHLSSG
NVLVSDSMDVENVVADSNSSSDGLVIDNLLGKLVADKYFDGVALCSTSVVQLTTAFRILAF
ISDDKAVASSLFEEGAITVIYIVLMNCKSMLERISNSYDYLVDEGAELSSTTELLLDRTHEQ
AIVDLMIPSLVLLINLLHILRETKEQYRNKKLLSSLLQLHREVSPRLAACAADLSFMFPTFAI
GFGVVCHLITSALACWPLYNWAPGLFHCLLENIEATNASVPLGPKAAISLLCLLGDLFPDE
GIWLWKVELPSLSAIRSLSTGTVLGPQVEKDVNWYLHPEHVAILLVRLMPQLDRLARIIDN
FATSALMVIQDMLRVFIVRVASEKIECAVVLLRPIFIWLDDKVDKTSLSEREIFKVHQLLQFT
VKLSEHPTGKVLLWRMEFTRILRKLLQNCSRSSFSDDNQTFGRAPSKNDLMLKWRIPLFK
SIACVFSIDTSNNEKAVIEESLNEKSVHECSSVMQHLVMFCQVLPVGREMLACSLAFKELA
ASYTCRSAVTLILSQIHTSNKDVLEKDESDPNHNLPTLDGWNCFSSLFNCWKKLAKYIGSN
QPTDYLVETIYSLTLGAITLSQYGENLEGLLILRYLFGLPSDPSGSLESSGESPSEIELFMKTS
EEKICQSFENSTTVDGKTLLHKLLNSITLLRSILENSGQSADSVQMVIQEGTDSLSEIAHSV
VMTADLMPSLANVSVKDESPFLFSNVWKVIVDSEEPLDCQEGEFAKRLVWELPDSSLDRQ
LTPGQSARRKLALGESASRRVRDNQLPEPTGQFSRGLNTTNASSGHTRRDTFRQRKPNTS
RPPSMHVDDYVARERNIDGASSASNIVNSTPRGTLSGRPPSIHVDEFMARQRERQNPVPAP
TGDAPQPKSQTASLDGSLRTKPENLRQPKTDLDDDQEIEIVFDEESGSDDKLPFPQPDDSL
QSPPVIIGENSPGPVIETENQENERIPFSQRATSLPKDDESPGVDISSQTAMLSEPNNSLELKY
SVSSPGKNSFRDHAEKSNYPSIGVSGRSSVQADHQHLSRRHEKRSPRKYSETSLSSGSHGH
EHRHSNNHPPLPPMPPPISSVPMQNTDSANRQSSSFSARDRPTPSLSGYPTQSFDSSMPSAF
TGLQGQTQYMLAGAGGSSANDLPNAEAKLLWNTFPVNRIPLETFSSGLSARPMPPLTPYS
AVATQHAPMSSSSPATLYNQGSVVQPSPTASIISDSNLAMNSNLLPSFASQFLMGRPSMPTPF
FGTPLQQVQFSSGLPQNISNSQPSVSSVQPRPPPPPPPPQQPHPSQTLQQLGAIQLPHQDQQ
LPYPQSAILPQVPLQFPNQLPIPQLQLYHQSQQESGQTLRQVGEQSQLQNQGMQADSFSQ
QQQDSGINLNQFFSSPEAIQSLLSDREKLCQLLEQNPKLMQMLQDRIGQL
>ZmPHB47.05G080000.1
MGRPEPVVLFAQTILHSQLDEYVDEVLFSEPVVITACEFLEQNASPSTPNISLVGATSPPSFA
LEMFVHCDGESRFRRLCHPFLYSHSSSNVLEVEAIVTNHLVLRGTYRSLTLVIYGNTAEDL
GQFNIELGLDHSLANVVSSPSEGKLEDLPPALHSSKLSFEESLSSLKPSSFHATDVDLSIEAK
KVLHLALKMYQISDVENLIPNLRSAVLSAISKYVTASTNHILHTLSQDSANAFINSDFDSQE

INKILAEAGKELSEIWKNVHAVTESNLFNDNGFTIGVDEDLPTTKILIELFNHCFPYYKKLS
LLDLQCPSQSKWLVLSLSLVLLLCSSKESCFYFVDTGGMEQIINLLCWRTPKSAATTLLLLG
IVEHATRNGFGCEAFLGWWPRAEHTSIPVGSSDGYCSLLKLLLEKERHDIASLATYVLQRL
RFYEILSKYESAVVKVISNIQADKLSTDGVPFLISASVELGEMLKLIIFCGPIEDPSPVATASRI
FKSEHLEGLLSYKATIDLITSSKYSFLQYDTDPYLLSLIQERSFFPLSAALLSSPILHLPNGPA
AEILMGIASSIESIILSLLFCRSGLSFLLSQPEATELILLSLQDAEKMNKSECITLRQAFVLLSK
GFFCRPKEVGLITELHLKVGSAATRILSVPPNSDELLWVLWELCAISRSDSGRQALLALGY
FPEAISVLLRSLSSYKDLDSVMAENGGSPLGLAIFHSAAEILEVLVVDSTASSLESWIGFAV
DLHKALHSSSPGSNRKDAPTRLLEWIDAGVVYQRNGARGLLRYSAILASGGDAHLSSGN
VLVSDSMDVENVVADSNSRDGQVIDNLLGKLVADKYFDGVALCSTSVVQLTTAFRILAFIS
DDKPVASSLFEEGAITVIYVVLMNCKSMLERLSNSYDYLVDEGAELSSTTELLLDRTHEKA
IVDLMIPSLVLLINLLHILRETKEQYRNKKLLSSLLQLHREVSPRLAACAADLSFMFPTFAIG
FGVVCHLITSALACWPLYNWAPGLFHCLLENTEATNASVPLGPKAAFSLLCLLGDLFPDE
GIWLWKVELPSLSAIRSLSTGAVLGPQVEKGVNWYLHPEHVTILLVRLMPQLDRLARIIDN
FATSVLMVIQDMLRVFIVRVALEKIECAVVLLRPIFMWLDDKVDKTSLSERDTFKVHQLLQ
FTVKLSEHPTGKALLWRMGFTRILSKLLQNCSRASFPYDNQTFGRAHSKNDLMLKWRIPL
FKSIAYIFSIDPCNNEKADVEESLNEKSVHECSSVMHDLMMFCQVLPVGREMLACSLAFK
ELVYSYTCRSAVTLILSQIHTSNKDVLEKDESDPNHNSPSLDGWNCFSSLFKCWKKLVKY
VGSNERTDYLVETIYSLTLGAITLSQYGENLEGLLILRYLFGLPSDPSGSLESSGESPSEIELF
MKTYEEKICQGIENSATAVGKTLLRKLLNSIKLLRSILENSGQSADTVQMVLEEGTDSLSEI
ARSVVMTAHLMPSLANVSVNDESPFLFSNMWKVIVDSEEPLDCQEGEFAKRLVWELPDSS
LDRQLAPGQSARRKLALGESASRRVRDNQLPEPTGQFSRGLNTSNASSGHTRRDTFRQRK
PNTSRPPSMHVDDYVARERNIDGASSASNIVNSTPRGTLSGRPPSIHVDEFMARQRERQNH
APAPTGDAPQAKSQTTSLDDSLRTKPENLRQPKTDLDDDQEIEIVFDEESGSDDKLPFPQP
DDSLQSPPVIIGENSPGPVIDETENQENERVPFFQRATSIPKDDENLGIGISSQTAMLSEANSS
LELKHSVSSPGKNSFRDHAEESNYDSIGVSGRPSLQADHQQLSRRHEKRSPRKFSETSLSN
GFHGREHRHSNNHPPLPPMPPPISSVSMQNTDSVNRQSSSFSARDRPTPNPSGYPTQSFDSS
MPSAFTGLQGQTQYMLAGAGSSSANELPNVEAKLLWNTFPANRIPLETFSSGLPARPMPPL
TPYSAVATQHATMNSGSPANLYSQGSVVQPSPTASIISDSNLAMNSASGSMLASNLLPSFAS
QFLMSRPSMPTPFFGTPLQQVQFSSGLPQNISNPQPPVSIQPRPPPPPPPPPQQPHPSQTLQQL
GAIQLPHQDQQHSYPHSAILPQVTLQFPNQLPIPQVQLYHQSQQESGQTLRQVGEQSQLQN
QGMQADSFSQQQQDSGINLNQFFSSPEAIQSLLSDREKLCQLLEQNPKLMQMLQDRIGQL
>OsKitaake03g230201.1
MGRPEPVVLFAQTILHSQLDEYVDEVLFSEPVVITACEFLEQNASPSTPNISLVGATSPPSFA
LEVFVHCDGESRFRRLCQPFLYSHSSSNVLEVEAIVTNHLVLRGTYRSLTLVIYGNTAEDLG
QFNIELDLDHSLANVVSSPSEGKLEDLPPALHSSKFTFEESLSSLKPLSLQATELDLSIEVKKI
LLLALTMYQIPNVENLIPNLQSAVISAVLKYMPASTNCMSRNWNRDPANCFAEDNVDSQG
TSNTLLMEASNELFDIWKNVNSIVDNITFDDNGLAFRLEELPTTKHLFTLFDSCFPYYRNC
SLLDLECPFQSKRLVFSLSLVLLLCSSKESCFYFVDAGGMEQIINLLCWKTSISPATTLLVLG
IVEHATRYVVGCEAFLGWWPRSDDNNIPIGSSVGYCSLLKLLLEKERHDIACLATYVLQRL
RFYEILSRYESAVVNIVSNLPSEELSSDGVNFLSSASIELAELLKMINMCVPIEDPSPVLTARR
ICKFGHLEGLLSYNLTIGLITSSKYSFLQFDADPYMLSLIQERGFFPLSAALLSSPVLRLASG
PAAEILMEIASSIEALVLSLLFCRSGLSFLLGQPEATELILLSLQDGEDMSKTECMTLRQAFV
LLSKGFFCRPQEVAMITELHLKVGSAANRLLAVPPNSDELLWVLWELCSISRSDSGRQALL
TLGFFPEAVSVLLSSLSSYNDLDSVTNKNGGSPLGHAIFHSTAEILEVLVADSTASSLKSWIG
FAIDLHKALHSSSPGSNRKDAPTRLLEWIDAGVVYKRNGAVGLLRYSAILASGGDAHLSS
GNVLVSDSMDVENVVADPNNTDGQVIDNLLGKLVADKYFDGVALCSTSVVQLTTAFRILA
FISEEKAVASSLFEEGAINVIYVVLMNCKSMLERLSNSYDYLVDEGAELSSTTELLLDRTHE
QTLVDLMIPSLVLLINLLHILNETKEQYRNKKLLTALLQLHREVSPRLAACAADLSFMFPSF
AVSFGVVCHLVTSAIACWPLYNWAPGLFHCLLENVEATNAAVPLGPKDACSLLCLLGDLF
PDEGIWLWKVEVPSLTAIRSLSTGTVLGCQVEKHMNWYLHPEHVSILLVRLMPQLDRLAC
VIDNFATSALTVIQDMLRIFIVRIASEKIECAVVLLRPIFIWLNNKVDETSLSEREIFKVHQLL
QFIAKLSEHPNGKALLCKMGVARILRKLLQECSSMCYMEDNMISDKGVYSNDLLMLRWK
IPLLRSIASIFSTRPSSKEPTTVEELWNENACVEECSSIMYHLLMLCQVLPVGRDMFACSLA
FKEVASSYSCRGAVTSIFSQIQTSNKDESQKSESETCHDTSKVDNWCGFFPLLKCWKRLLQ
YICANRPTDYLVEIVYALTLGAIALSQSGQNLEGTIILRRLFGHPSVPSSSEASDEVTFLLKT
FQEKICQGFDNWSPYVGKPLLHQVRSSVRLLCSIIENSGPFTDSVRMVLEESTIPVGVFHNI
VMTSHLMPSIDFVSVNDDPALLFTNAWKAFGDFAEPFGCQVSDFSKRMVWELPDCSIDKQ
LIPSQSARRKLALGDSASRRVRDNQTHEPSGQFSRGLNTPSASIGHTRRDTFRQRKPNTSRP
PSMHVDDYVARERNIEGASSASNIVSSTPRGALSGRPPSIHVDEFMARQRERQNPVLAPSG
DATQVRSKATLDDNVSTKPEKPRQPKADLDDDQEINIIFDEESGSDDKLPFPQPDDSLQSPP
VIIGENSPGPVVDETENQQNGINLFSGTVVSESDEACETVISSQTAIRQESNIPSERKFSVSSP
EKVMFPDHADESPFISPTTGLKVIPGYSTHAAQATLRQLPPNMHRKRSPHKLAESSVSSGS
HGHDRTLYNSQPPLPPMPPPVSSTSLQNPDSIQRQPSSYIARDGPPPFPPSYLMQSFDACMP
SFVGHQVQTENVLPSTGDSSSNALPSVDAKFLWSTLPVNRIPMEHLSSGSSTRPVSPLPLRP
VLATQHAAMDSGPPGSLYNQGGSGVLQPSPPASLINDATLGTNPASGGALASNSLPSLASQ
YIIGRPSTPPFFGTPLQIQLSSGLAQSVSNPQPSLSSMQPRAPPPPPPQPHPSQTFQGSLQQPQ
EQPMPYPLNTIQPQVPLQFPNQLHVPQLQFYHQTQESVLQPIGQSAQQQMDSGMNLNHFF
SSPEAIQSLLSDRDKLCKLLEQNPKLMQMLQDRIGQL

>OsVIR
MGRPEPVVLFAQTILHSQLDEYVDEVLFSEPVVITACEFLEQNASPSTPNISLVGATSPPSFA
LEVFVHCDGESRFRRLCQPFLYSHSSSNVLEVEAIVTNHLVLRGTYRSLTLVIYGNTAEDLG
QFNIELDLDHSLANVVSSPSEGKLEDLPPALHSSKFTFEESLSSLKPLSLQATELDLSIEVKKI
LLLALTMYQIPNVENLIPNLQSAVISAVLKYMPASTNCMSRNWNRDPANCFAEDNVDSQG
TSNTLLMEASNELFDIWKNVNSIVDNITFDDNGLAFRLEELPTTKHLFTLFDSCFPYYRNC
SLLDLECPFQSKRLVFSLSLVLLLCSSKESCFYFVDAGGMEQIINLLCWKTSISPATTLLVLG
IVEHATRYVVGCEAFLGWWPRSDDNNIPIGSSVGYCSLLKLLLEKERHDIACLATYVLQRL
RFYEILSRYESAVVNIVSNLPSEELSSDGVNFLSSASIELAELLKMINMCVPIEDPSPVLTARR
ICKFGHLEGLLSYNLTIGLITSSKYSFLQFDADPYMLSLIQERGFFPLSAALLSSPVLRLASG
PAAEILMEIASSIEALVLSLLFCRSGLSFLLGQPEATELILLSLQDGEDMSKTECMTLRQAFV
LLSKGFFCRPQEVAMITELHLKVGSAANRLLAVPPNSDELLWVLWELCSISRSDSGRQALL
TLGFFPEAVSVLLSSLSSYNDLDSVTNKNGGSPLGHAIFHSTAEILEVLVADSTASSLKSWIG
FAIDLHKALHSSSPGSNRKDAPTRLLEWIDAGVVYKRNGAVGLLRYSAILASGGDAHLSS
GNVLVSDSMDVENVVADPNNTDGQVIDNLLGKLVADKYFDGVALCSTSVVQLTTAFRILA
FISEEKAVASSLFEEGAINVIYVVLMNCKSMLERLSNSYDYLVDEGAELSSTTELLLDRTHE
QTLVDLMIPSLVLLINLLHILNETKEQYRNKKLLTALLQLHREVSPRLAACAADLSFMFPSF
AVSFGVVCHLVTSAIACWPLYNWAPGLFHCLLENVEATNAAVPLGPKDACSLLCLLGDLF
PDEGIWLWKVEVPSLTAIRSLSTGTVLGCQVEKHMNWYLHPEHVSILLVRLMPQLDRLAC
VIDNFATSALTVIQDMLRIFIVRIASEKIECAVVLLRPIFIWLNNKVDETSLSEREIFKVHQLL
QFIAKLSEHPNGKALLCKMGVARILRKLLQECSSMCYMEDNMISDKGVYSNDLLMLRWK
IPLLRSIASIFSTRPSSKEPTTVEELWNENACVEECSSIMYHLLMLCQVLPVGRDMFACSLA
FKEVASSYSCRGAVTSIFSQIQTSNKDESQKSESETCHDTSKVDNWCGFFPLLKCWKRLLQ
YICANRPTDYLVEIVYALTLGAIALSQSGQNLEGTIILRRLFGHPSVPSSSEASDEVTFLLKT
FQEKICQGFDNWSPYVGKPLLHQVRSSVRLLCSIIENSGPFTDSVRMVLEESTIPVGVFHNI
VMTSHLMPSIDFVSVNDDPALLFTNAWKAFGDFAEPFGCQVSDFSKRMVWELPDCSIDKQ
LIPSQSARRKLALGDSASRRVRDNQTHEPSGQFSRGLNTPSASIGHTRRDTFRQRKPNTSRP
PSMHVDDYVARERNIEGASSASNIVSSTPRGALSGRPPSIHVDEFMARQRERQNPVLAPSG
DATQVRSKATLDDNVSTKPEKPRQPKADLDDDQEINIIFDEESGSDDKLPFPQPDDSLQSPP
VIIGENSPGPVVDETENQQNGINLFSGTVVSESDEACETVISSQTAIRQESNIPSERKFSVSSP
EKVMFPDHADESPFISPTTGLKVIPGYSTHAAQATLRQLPPNMHRKRSPHKLAESSVSSGS
HGHDRTLYNSQPPLPPMPPPVSSTSLQNPDSIQRQPSSYIARDGPPPFPPSYLMQSFDACMP
SFVGHQVQTENVLPSTGDSSSNALPSVDAKFLWSTLPVNRIPMEHLSSGSSTRPVSPLPLRP
VLATQHAAMDSGPPGSLYNQGGSGVLQPSPPASLINDATLGTNPASGGALASNSLPSLASQ
YIIGRPSTPPFFGTPLQIQLSSGLAQSVSNPQPSLSSMQPRAPPPPPPQPHPSQTFQGSLQQPQ
EQPMPYPLNTIQPQVPLQFPNQLHVPQLQFYHQTQESVLQPIGQSAQQQMDSGMNLNHFF
SSPEAIQSLLSDRDKLCKLLEQNPKLMQMLQDRIGQL
>Aco001714.1
MGRPEPYVLFAQTILHSQLDEYVDEVIFAEPVVITACEFLEQNASPSTPSISLIGATSPPSFAL
EVFVHCEGESRFRRLCQPFLYSHSSSNVLEVEAIVTNHLVLRGSYRSLTLVVYGNTAEDLG
QFNIEVDLDNSLTNVVCSPSEGRLEDLPLALHSSELAFEESVSSLKSLGFRSPEFDILPEVKQ
FLLLAFQICQLVDTNDMASNVVSAVESVASSCAVVNTDSALHSWDQELLSALVGSKRDSP
KFLNVLADARNELLEIWKNLQSENGSCELMEDELETQLPTTEMLVDMFYQCFPFFRKAST
LDLPFFSQSKNLVFALGLILLVCSSREGCSHFVSGGGMDQIIHLLHREIPKSTATTLLLLLIIE
CATRHGIGCESFLGWWPRRDFVVPFRVSDGYCYLLELLLEKQRDDIASLSTYVLHRLRFFE
ILSRYESAVVFLLTNLPADGQLATDGVTSLVDANSQLKHILERGFFPLSAALLSSATLRSASG
SAADSLSFLLVQPEATELLILAFQDGEDISKTECMTLRQATVLLSKGFFCRPQEVGMIIELYL
RVVTAVSRLLAAAPNSDEFLWALWELCAISRTDTGRKALLALGYFPEALSVLLEALRSYK
DLEQTAITSENVFGDSTSSSDGLVIDNLLGKLVSDKYFDGVPLTSTSIVQLTTALRILAYISED
SAVAATLFEEGAVTLVYVVLVNCKSMLERWSNSYDYLVDEGAESSSTTDLLFGRSHEKRL
LDLIIPSLETKEQYRNKKLLNALLQLHREVSPALGHGAICHLITSAVASWPIFGWAPGLFHS
LLENIQATSSAALGPKDVCSLLSLLGYLFPDEGIWLWKNEMPPLSAVRALSIATVLGPQVE
RQINWYLLPEHSSLLLIRLTPQLDKIAQVVLHYATSGLVVIQDMLRVLIARIASQRAECAVV
LLRPTISWLDNHVDESSLSDTDIFKVHQLLHFIASLLEHPNSTALLSKMGTVRILGKVLEIC
SNAFYSEGKLTRESRGPNQELEQDERDTDENYSNEYSWLQSLPFLKCWKKLLRSLDSNDS
CANFVVETVYVLSLSAVCLSLEGDSVEGINILKYLFGLPCEPGGAADISDEKLNEVVNLLK
TLEGNIAENENSTAIVGKSALDHVKCYHVKESLKAVSLLLHSTSDSLSNSQLAVCTESLETP
SNAIRSIVMTSQLMPSLSTLPLNEEAALFFSNAWKFIGDSEKTSNDFPNGEFLEKFVWECSD
SSLGKHLLPAQSAKRRLPPGDGSSKRTRDAAGSEATTSNVFSRGVNAQNAPLGPTRRDTF
RQRKPNTSRPPSMHVDDYVARERNIDGASSGSNIVNTNQRGGSISGRPPSIHVDEFMARQR
ERQNSIPTLAAGDASQPKSSDLLNNNGPPKPDQPQKLRADLDDDEIDIVFDEEPGSDDKLP
FPQPEENLPSPTVAVVKSSPGSVVEESEGDRNGNVRFNQIGSTPQASRSQISTTQELSVPSEK
NTSLRDRSESNYSSQPSTNIAGFPSPFSNARSSAPLPLQQFSSSYLYQGKSPQKEQTLGNAQ
PPLPPTPPPAAVVSSPFVNTSRDVQPPLPTGYPLQAFDVGGPNNVAGLQLQSENMLSTGNG
SWNSITGSRMHLPPLPPPPYSNPITHSPALHSGSPASLYNPSTSNVGTLTLPTPSLISDTGLGI
LPASGNNNLSAYSLPPFTPSLLINRPNSLPGSVFSPQQGQIPSNLSHILPNSQPSSIQPRPPPPP
PPPPQLPHPSQTPQQATPIQMPQPHAEQAMPFAQSSVQLQVPLQFQQQLHVPQMQFYYPT
QQLESVLPQPSQPAVEHQQPQNQGLQVDSLSQQQKDAGISLQQYFSSPEAIQSLLSDREKL
CQLLEQHPKLMQMLQERLGQI

>Pp3c22_17700V3.1
MAALPRCSLLFADSFHHDRLCQHVDEVRFTEPVVITACEVVELHQPSRCPTLALKGITSPE
SCALEFYVRSGRDSRFQRLGPAFLHSPAAIPFLDVQAAVTEHLVLRGSYKSLTLVIYGNLAS
ELCVDNNPDTNNVSQRATSKIVSLREILCTQPSSILSLSNLQLYTPPTKEQGTLLQRLIKYVD
GRDATQHMVSMLLTAAAAWHLSHQRQREGAQWCALKKDDPLDGSRSLLCDDSIREMSE
LHNLLQQKEGDDKHTSSDEANGRVLVQLSLHWLQVGLDPSAGLNSSISAVESLLGGLAAV
QLLFTSCPKDVPAFLAGGGMRLLHQVVEEISGTSALLLFALTSVECTLRHASGCEEFSAQD
KRILLSLLEKKQRPPVVSLLQRILQRLRCYELAVTITRLVEPLTNLGSAQNSDTIKEASKLLN
GLLIALSIKSFVESTSVEYNWVKQEAKLPTSAGSCYDDFTSLSEDVDCALLSILQETSFMES
LAAFSSVPHIWPANGSAPNVSIEFASATQRFLLRLLECRSGLLFLGADMESLTKMITGLKSA
SEQELEVLPIRHVTVLSSLGYLCTPGSMAETLHSRISMVSAADRFVGADAGSDAAFGALW
DLASMSRSEAGRQAILAIVSFPEVLTALIEAIHPSPDLAESMGGAGGHFATEALQKVFCDSA
LVNQIALTVHASALQTALQAACNSGMNSCSKLVEWVEAAVVYQKKGAVGLLKHATALIG
PLNTSMTIHVDGSMGDDANNLHDRAPGSELPNSISKGTGSTPIQYSAIHALTISLRLLASAG
WNLEVAASLYGDGAVGVVNIILEHCVAALQASSNEFDDEDTEEDEGRGEDFHKEQALLAL
LLPTLILLLSLLKCLQSVVQHFCSTRLLDLLTQLHHVISAKAGAHETAPPYSWTGEVLELG
AVQQVLASLLAFWPVQGWTPALMPRLFGSNSTSLPMEPTEACSVICLLEEFLPPECPRTFM
DKAAVLDVYRTVAVGNIHGVEVMPAVYWHTSPKHTDKMLHVLSPFLEQIGQLVVHLASC
TCDVVQERLTNLVVRLACQSSENAVIVLRPVLSVLRQRTAAAAKSLLSETDVLQVTHMLR
WIALLTAHASSKALLLQEGIVQMLLQVISLEATPSIPVGKSGWLMWSLRTVARLCDTEVCF
LPAASVARHISEDCPNYDDCCAIASSLLQHCQLMHVEPQIEVLADAFEKLASHDVGRAAV
ASVALGHAGCATHMNNHDTPESQISGVFHKASPPFLKLWQNLVSAMGLVELNPVLVRLVR
RFAQVAIVLTESGHSSVGGVALRALFGLDVSSESTESKTVGERDDKLEAPSCVVSILQQHLT
DIEDSNATFSVFRSSLHEALSAVSSMLNYLTASEKEIVLREEIELLINRLPAARPVAERRAEAI
VAQSSSCFGLVSFMDDGVKPMDTDISEAINGESKGTIPWDCPKLPHERPVMKMRTSKRRVI
STIEVASKRQRGDGATSAVASRTPVLVTSSRRDTFRLRKPNTSRPPSMHVDDYVAREKSSD
VLSGSSPAAAATTLQRSNSGTRRAPSIHVDEFMARQRENQQSDIPLIQGYAENGGSRAVLD
LLDGDNGADLCLTDRNLRISQTTAEYSSVSVIVSSVATFSAAPPPALAMMNTSVINSEVLQR
NPNFHSASEHTGFSSQRLGGMKMEQNNGGTCPGVEINSQPPSSVVKVKLERAPSMNSVS
AGLPSPFQPYEQEFNAAVAAAVKMEPRQQPDSNIMPRTLHAEVPAAPPQQQASQSDLSSHL
PPPPPPAPPSPWLDQPRRLDPSLLPPVIPRLIPGMNVLPYNHGSGSRPPADLQVGGVVLQPP
AGLWRDSTPVFSGLPPPAPPIPGPNPHGPQFVQQSFMGASRPLDTLQGYPGNHAGTASTSD
HRFGNLSGPPSGQGSSPLFQPPIPTGMPPSQNTLSSEQSVHRPPPLHLQESEIIAQQEPGAVL
QQILQSPDTIHELLKDTKKLQQLLEQHPKLVALLQEKISHGML

>Ptrif.0006s1372.2
MGRPEPCVLFAQTFVHPQLDEYVDEVLFAEPIVITACEFLEQNASSTSQAVSLVGATSPPSF
ALEVFVQCEGETRFRRLCQPFLYSHSSSNVLEVEAVVTNHLVVRGSYRSLSLIIYGNTAEDL
GQFNIDFDDSSLTDLVTSAEGKLEDLPLALHSINRTIEESLSSLKVLSLPVAPSDISIEVKQLL
HLMLKVFELPNPESAVHKTVSTVVLAASSFVAHDLGDTTRQKHLASGKLKSNEDDLQHA
VIKARKELIQLYAALQHDSGNDSAELLGDCAFFETEADVASSKELVDMLSQYFNFSGNSTS
LGHHNLPQNKSVILGLSVALLLCSGREGCFHFVDSGGMDQLAYVFSRDIENSTAIMLLVLG
AIEQATRHSIGCEGFLGWWPREDESIPSGISEGYSRLLNLLVQKPRHDVASLATFVLHRLRY
YEVASRYESAVLSVLGGLPAAGKVTAATSNMLISAKSQLKKLLKLINLRGPIEDPSPVSSAR
RSLTLVQAEGLLSYKVTSNLIASSTCGFSNSDIDPHLLTLLKERGFLSLSAALLSSSILRTEV
GDAMDVYLDIASSIGAIILSLLFCHSGLVFLLHHYEISATLIHALRGVTDLNKEECVPLRYAY
VLMSKGFTCGLQEVATIVEMHLRVVNAIDRLLTSTPQSEEFLWVLWELCGVSRSDCGRQA
LLTLGFFPEAVSMLIEALHSAKEQEPSTKSGGASPLSLAILHSAAEIFEIIVTDSTASSLGSWI
GRAMELHKALHSSSPGSNRKDAPTRLLEWIDPGVVYHKSGVIGLLRYAAVLASGGDAHLS
STSNLVSDLMEVENATGEPSSGSDFNVMENLVKIISEKSFDGVTLRDSSIAQLTTALRILAFI
SENSAVAAALYEEGAVTVVYTILVNCRFMLERSSNNYDYLIDDGTECNSSSDLLLERNREQ
CLVDLLVPSLVFLITILQKLQEGNEQHKNTKLMNALLRLHREVSPKLAACAADLSSPYPNS
ALSFGAVCRLVVSALAFWPIYGWTPALFHSLLVSVQTTSLLALGPKETCSLLCLLNDLFPEE
DIWLWRNGMPSLSALRTLAVGSLLGPQKEREVEWYLEPGRREKLLTQLRPHLDKIAQIIRH
YAISALIVIQDMLRVLIIRVASQKSENASLLLRPILAWIRDHVSDSSSPSDMDVYKVHRLLDF
LASLLEHPCAKAVLLKEGVPQMLIEVLKRCFEATDSDGKQFSDQLNSVKIGSTLTSWCLPV
FKSFSLLCCSQTPMQHPGSHDLYKFDNLSADDCSLILPHILKFCQVLPVGKELVFCLTAFRE
LVSGGEGQRALISIICHTHSTLEEFDSGRGHESNDDRSLLNEFEWRKNPPLLCCWTKLLNS
VDSNDGLSTYAVEAVCALSLGSLRFCLDGKSLNSNAIVALKYLFGLPDDKSGTESFPEENV
KLIQKMFTVLSKINDDNYYSAIPDLQTSLCQVLEFVKVLLLLLQKPTGSVDVDNVMFTEGI
LPSPNDIVLSNIHQMVGGNVEKDDDKLYLVGLEDKFMWECPETLPERLSQTALPAKRKMP
PVEGLSRRARGENSAAETNQNTFSRGLGPTTAPSGPTKRDSFRQRKPNTSRAPSLHVDDYI
AKERSGEGVSNSNVIIAQRVGSAGGRAPSVHVDEFMARERERQKRMVTVVGEATIQVKN
EAPTSGTQKEKVDKPKQLKTDPDDDLQGIDIIFDDEESEPDDKLPFPQLDDNLQQPAPVIV
EQSSPHSIVEETESDVNESGQFSHMSTPLASNADENAQSEFSSMMSVSRPDVPLTREPSVSS
DKKFFEQSDDSKNVITAKASGVFDSGAAANSPGFSASLYNNATGSSMPTDSRMNQNFYPK
NSPQHAANLPVGTGSRGLYDQKVMPNQPPLPPMPPPQAISPGMSQASDSIPSHSSPYVNSL
TEVQMSVPPGFQVQADYLSAFSGSSTPGGSSRPPLPPTPPPFSSSPYNLPSFKANSQMSMY
NQNIGGTTDLPQAQSSIVPMIDARLGSVSASAAGVSYPPPHIMPPLVFNRPASIPATLYGNTP

```
AQQQGEIMQNLSIPQSSIQSMHSLAPLQPLQPPQVPRPQQPPQHLRPPMQASQQLEQVTSL
QNPVQMQVHPLQTMQPPQVSPIHTYYQSQQQEFSPAQQQQQVERTQPQVQHHQGDIGSQ
QQQDPAMSLHEYFKSPEAIQSLLSDREKLCQLLEQHPKLMQMLQERLGHL
>DmVIR
MADVDDGSELLFFDTFSHEEVTDINLDLVQFPKPVFITQVRIIPLGARVQADFPGGVRLGAT
NPSKFDLEFFVNDLGMPAASAFENLGLLRYNQNDCIHLDCSQEKIVTDGLVLRGWYSTITL
AIYGIFTNSVTEPIASPTLPCEPVGPEIANLSGEVLLQEDVLKDEWQEPMQAELLTAHKGN
VSDYDPEDMEYGMSRDHYHQHAEEQEQREMRRLRRSTHSTDHSPPPPRRSHTHSESNDR
EYIRCSRDKGSRDWSRSPEYSSHRSRRKRSERSRSVVDEHKWPRTPPASIDSPTRPRSPDT
MDYEDEDSRSHYKMQSSHYRHSSESLHRGERDRDDEDRSCTPQEQFEPILSDDEIIGDDEE
DDAVDAAAIAEYERELEAAAAAAPPAIDAFEPWQKPLLVFEGDMAAHFCKELETLKLLFK
KLVLQTRCENVNAFSEEHGASVDEREQFVYLGEQLNNQLGYLAQHYKRRNFVLQQFFGN
DELHLRQAANVLQIALSFQAACMQPQPAFKIRHIKLGARMAELLGSSEELFQHLLKEHKF
DIFEAVFRLYHEPYMALSIKLQLLKAVYALLDTRMGIEHFMGAKNNGYQMIVEAIKTAKLT
RTKYALQAIIKKLHLWEGLESVQIWCRRLFVDRIIIPGNRDQMEDTVITCQQIEFAFEMLMD
ALFSSQLSYLQPRRFLPVSKKFEVVTDPTAQRSFGNALQSYLGQNSLAESLLVMLANCKE
LPATTYLSMLDLMHTLLRSHVGIDYFVDDAFPVTQTIVAILLGLDEVPRNPEEKEEKAEKS
DAEDKAMEVENEAVEAGGEKPTPPTADEEGKPVAAPISVPAPAAAPQVRPRPILRPVLPRL
ARLGIEMSYKVQTRYHLDAIAYAAAAPEYDAVKLATHMHAIYSQTCDPAGRQHTVEVLG
LNNNLKIFMDLIKKEQRLQTQRQLSSPGTKYKSPVLSYAVDMVDACVRYCEQLDYLIEHG
GVILELAKNHETFEPSVSAVLQEMYVYMKPLEAINVFVYDDIMPLVEVIGRSLDYLTTFPG
DLIMAMRILRYLSISKPLAGQKAPPVTEELKHRFVALQLYAADGVQLCIQIMERLCAYFEQ
PGAHAPALMTIQGVHCCQIMLPTLQILRELLSYAILCRDGTYKDLTAIDHLVKVYYLLYYFP
TRCQAGPEVEQCKMEVVQTLLAYTQPNEQDEESLHKSLWTLMIREVLKNVDGPAHFIPGL
KLLAELLPLPLPMPQPLCDQLQQQHKQRLITERKLWSAHLHPQSGQIAKLVEALAPSSFPQ
LSELLQRVCMQLSDLAPNMTLLIAKTITELLCNEYQTSNCIPTTNLERLLRFSTRLCAFAPL
KSSMLSILSGKFWELFQSLLALNEFNDVVSNCQEAVHRILDSFLDSGISLISHKSTASPALNL
AAALPPKELIPRIIDAVFSNLTSVEVTHGISILAVRNLVILTEHDFTFYHLAQLLKQKITEFQA
WMERVILHNETVEYNANIESLILLLRSLTQIEPPPAMSAMPHRTLKLGATELAQLVEFQDIE
LAKPPVLSRILTVMEKHKAVANEAALSDLKQLILLQASKQEILAGTSTETPPEAEGEANPSA
SSCSASLTVEPYLPQAEGIVTQYEARPIFTRFCATAENAQLTARYWLDPLPIELIEDMNEPIY
ERIACDLTDLANVCLNPDLNVAGDSKRVMNLSGSPQSNREMTPTAPCFRTRRVEVEPATGR
PEKKMFVSSVRGRGFARPPPSRGDLFRSRPPNTSRPPSLHVDDFLALETCGAQPTGPTGYN
KIPSMLRGSRVGRNRGSRISAAAAFRQKKMMRIGSPSSWAESPGSYRSASDSHFSSSDSHY
SSPHYSGRPRGRGLRSRPSYLR
>HsVIR
MAVDSAMELLFLDTFKHPSAEQSSHIDVVRFPCVVYINEVRVIPPGVRAHSSLPDNRAYGE
TSPHTFQLDLFFNNVSKPSAPVFDRLGSLEYDENTSIIFRPNSKVNTDGLVLRGWYNCLTL
AIYGSVDRVISHDRDSPPPPPPPPPPPPQPQPSLKRNPKHADGEKEDQFNGSPPRPQPRGPRTP
PGPPPPDDDEDDPVPLPVSGDKEEDAPHREDYFEPISPDRNSVPQEGQYSDEGEVEEEQQE
EGEEDEDDVDVEEEEDEDEDDRRTVDSIPEEEEEDEEEEGEEDEEGEGDDGYEQISSDEDG
IADLERETFKYPNFDVEYTAEDLASVPPMTYDPYDRELVPLLYFSCPYKTTFEIEISRMKDQ
GPDKENSGAIEASVKLTELLDLYREDRGAKWVTALEEIPSLIIKGLSYLQLKNTKQDSLGQ
LVDWTMQALNLQVALRQPIALNVRQLKAGTKLVSSLAECGAQGVTGLLQAGVISGLFEL
LFADHVSSSLKLNAFKALDSVISMTEGMEAFLRGRQNEKSGYQKLLELILLDQTVRVVTA
GSAILQKCHFYEVLSEIKRLGDHLAEKTSSLPNHSEPDHDTDAGLERTNPEYENEVEASMD
MDLLESSNISEGEIERLINLLEEVFHLMETAPHTMIQQPVKSFPTMARITGPPERDDPYPVLF
RYLHSHHFLELVTLLLSIPVTSAHPGVLQATKDVLKFLAQSQKGLLFFMSEYEATNLLIRAL
CHFYDQDEEEGLQSDGVIDDAFALWLQDSTQTLQCITELFSHFQRCTASEETDHSDLLGTL
HNLYLITFNPVGRSAVGHVFSLEKNLQSLITLMEYYSKEALGDSKSKKSVAYNYACILILVV
VQSSSDVQMLEQHAASLLKLCKADENNAKLQELGKWLEPLKNLRFEINCIPNLIEYVKQN
IDNLMTPEGVGLTTALRVLCNVACPPPPVEGQQKDLKWNLAVIQLFSAEGMDTFIRVLQKL
NSILTQPWRLHVNMGTTLHRVTTISMARCTLTLLKTMLTELLRGGSFEFKDMRVPSALVTL
HMLLCSIPLSGRLDSDEQKIQNDIIDILLTFTQGVNEKLTISEETLANNTWSLMLKEVLSSIL
KVPEGFFSGLILLSELLPLPLPMQTTQVIEPHDISVALNTRKLWSMHLHVQAKLLQEIVRSF
SGTTCQPIQHMLRRICVQLCDLASPTALLIMRTVLDLIVEDLQSTSEDKEKQYTSQTTRLLA
LLDALASHKACKLAILHLINGTIKGDERYAEIFQDLLALVRSPGDSVIRQQCVEYVTSILQS
LCDQDIALILPSSSEGSISELEQLSNSLPNKELMTSICDCLLATLANSESSYNCLLTCVRTMM
FLAEHDYGLFHLKSSLRKNSSALHSLLKRVVSTFSKDTGELASSFLEFMRQILNSDTIGCCG
DDNGLMEVEGAHTSRTMSINAAELKQLLQSKEESPENLFLELEKLVLEHSKDDDNLDSLL
DSVVGLKQMLESSGDPLPLSDQDVEPVLSAPESLQNLFNNRTAYVLADVMDDQLKSMWF
TPFQAEEIDTDLDLVKVDLIELSEKCCSDFDLHSELERSFLSEPSSPGRTKTTKGFKLGKHK
HETFITSSGKSEYIEPAKRAHVVPPPRGRGRGGFGQGIRPHDIFRQRKQNTSRPPSMHVDDF
VAAESKEVVPQDGIPPPKRPLKVSQKISSRGGFSGNRGGRGAFHSQNRFFTPPASKGNYSR
REGTRGSSWSAQNTPRGNYNESRGGQSNFNRGPLPPLRPLSSTGYRPSPRDRASRGRGGL
GPSWASANSGSGGSRGKFVSGGSGRGRHVRSFTR
```
