# Supplementary material for: Cotton (Gossypium hirsutum) VIRMA as an N6-Methyladenosine RNA Methylation Regulator Participates in Controlling Chloroplast-Dependent and Independent Leaf Development
Source: Int J Mol Sci. 2022 Aug 31;23(17):9887. doi: 10.3390/ijms23179887 (PMC9456376; doi:10.3390/ijms23179887)
Supplement: Supplementary file 1 [file ijms-23-09887-s001.zip › Table S2 primers.pdf]

Table S2. List of primers of this study used for different experiments.

Gene symbol	Gene name	Primers (5'-3')	Purpose
Gh_A11G2095	GhaTUB6 RT–F	ATCTGTGGCCGAGGTTTTCTC	qRT-PCR
Gh_D11G2411	GhaTUB6 RT–R	CTAGTAATCTTCTGGTTCTTC	qRT-PCR
Gh_A05G3802	GhbTUB6 RT–F	GTCTCTGACACCGTTGTGGAG	qRT-PCR
Gh_D05G1777	GhbTUB6 RT–R	CTTAGGCAGCATGTTACACCG	qRT-PCR
Gh_A12G0052	GhGID1B RT–F	CGATTGGATGGGAAGTATTTTG	qRT-PCR
Gh_D12G0066	GhGID1B RT–R	TTGCCAATCTTGAATGAGGTC	qRT-PCR
Gh_A02G0358	GhEIN2 RT–F	GCATGTTCTACTTGCTGATGA	qRT-PCR
Gh_D02G0417	GhEIN2 RT–R	CTACGGCTTCGGAACTTACAG	qRT-PCR
Gh_A07G0914	GhPTAC16 RT–F	TGAGGAGAAGCCTAAAGTCCA	qRT-PCR
Gh_D07G0989	GhPTAC16 RT–R	CAAACACCTTCCTCACTTCTG	qRT-PCR
Gh_A10G0408	GhmTERF1 RT–F	ACCTCTAACATCAAAGCTGAG	qRT-PCR
Gh_A10G0408	GhmTERF1 RT–R	GCAAGACAGGGTCTTGATAAG	qRT-PCR
Gh_A11G2019	GhSIG1 RT–F	CTCACCATAGTTACATTGCAG	qRT-PCR
Gh_D11G2299	GhSIG1 RT–R	CCTATGCGTTTACTGATGTCC	qRT-PCR
Gh_A08G0093	GhRPL12-A RT–F	GTTCGATGTGGTGATTGAGGA	qRT-PCR
Gh_D08G0135	GhRPL12-A RT–R	CTAAGCAATGGAAACTTTAGC	qRT-PCR
Gh_A02G0768	GhRPS13 RT–F	GAGCTCATTACGATTCGTGAT	qRT-PCR
Gh_D02G0814	GhRPS13 RT–R	CAGCAATGGCAACTCTCTTAC	qRT-PCR
Gh_A01G0976	GhPSI RT–F	GTCCACTGCTAAATCCACTTG	qRT-PCR
Gh_D01G1028	GhPSI RT–R	CTTGCACAAAGATGCCTAGCAT	qRT-PCR
Gh_A04G0428	GhPSII RT–F	GGTGTTTACCAATATGTTGACA	qRT-PCR
Gh_D05G3193	GhPSII RT–R	CAAAGCACTTGTATTGTAAACA	qRT-PCR
Gh_A02G0402	Gh50S RT–F	GAACAAAGCCAACAAAGTTTCG	qRT-PCR
Gh_D02G0455	Gh50S RT–R	TCATTCCTTGCGAAGATCTATC	qRT-PCR
Gh_A06G0106	Gh30S RT–F	GACATTGGGGTATGAAACGAG	qRT-PCR
Gh_D06G0079	Gh30S RT–R	CTGTGGAGCTTTACGAGCCTT	qRT-PCR
Gh_A01G0414	GhTCP3 RT–F	GTTCGTGCTTGGATAGACCAG	qRT-PCR
Gh_D01G0419	GhTCP3 RT–R	GCTTATTGGCAATGCTGTCGTG	qRT-PCR
Gh_A13G1272	GhTCP10 RT–F	TCAGAGGGGACCCCTTCAGTC	qRT-PCR
Gh_D13G1576	GhTCP10 RT–R	GAAGCAGAGGACAGCTTATTTG	qRT-PCR
Gh_A01G0139	GhGLK1 RT–F	GGACCCCGAAATTTTGGTTGAA	qRT-PCR
Gh_D01G0183	GhGLK1 RT–R	CATCGCCTTTACTCGTCGAA	qRT-PCR
Gh_A12G1283	GhGCN1 RT–R	TTTCCGCCTATGTCACCGTC	qRT-PCR
Gh_D12G1405	GhGCN1 RT–F	TGTTACACGTTTGGGGTGGT	qRT-PCR
Gh_A03G0593	GhPNSB4 RT–F	TGTTCACTTGCTGGGGATGT	qRT-PCR
Gh_D03G0881	GhPNSB4 RT–R	AATCAGCTCTTCACGCCACA	qRT-PCR
Gh_A08G1679	GhPSBW RT–F	CCAACAATGGCGAAGAAAGGG	qRT-PCR
Gh_D08G2029	GhPSBW RT–R	AGCTGGTGAGCTTGACATGA	qRT-PCR
Gh_A11G3190	GhPSAN RT–F	CTTCCCCAGTTATGTGCGGT	qRT-PCR
Gh_D11G2278	GhPSAN RT–R	AGCTGCCAAGTAGAGCACAG	qRT-PCR
Gh_A12G1042	GhGIF3 RT-F	AGCAACCTGGCATGTACCCTC	qRT-PCR
Gh_D12G1162	GhGIF3 RT-R	GCCTGATTCCCATTTGACCTT	qRT-PCR
Gh_A11G0749	GhGRF9 RT-F	AAAAGATGGCGATGCAGCAGG	qRT-PCR
Gh_D11G0870	GhGRF9 RT-R	TGGACGGCAATCACCAACACT	qRT-PCR
Gh_A07G2353	GhARF19 RT-F	CTCGTACAGAGCCAACAGCA	qRT-PCR
Gh_D07G0132	GhARF19 RT-R	GAGACTGCGACAGTGATCCA	qRT-PCR
EU604080	GhUBI1 RT-F	CTGAATCTTCGCTTTCACGTTATC	qRT-PCR
EU604080	GhUBI1 RT-R	GGGATGCAAATCTTCGTGAAAAC	qRT-PCR
Gh_A04G1327	GhVIR RT-F	ATTTATCTTCGGTTAGTGGC	qRT-PCR
Gh_D04G0886	GhVIR RT-R	GACGAGCATCAATAGTAGGTC	qRT-PCR
Gh_D04G0886	GhVIR EcoRI-F	CCGGAATTCGAAGCTTTGCATTCTGTCAAG	VIGS-TRV
Gh_D04G0886	GhVIR BamHI-R	CGCGGATCCGGACTTCATAGAGATAATGCT	VIGS-TRV
Gh_D04G0886	GhVIR PacI-F	GGGTTAATTAAGTTTTGATTGAAGCTTTGCATT	VIGS-CLCrV
Gh_D04G0886	GhVIR AvrII-R	CTTCCTAGGGTCAGCTGGGCTATAGAAGAGT	VIGS-CLCrV
