# Supplementary figures and images for: Cotton (Gossypium hirsutum) VIRMA as an N6-Methyladenosine RNA Methylation Regulator Participates in Controlling Chloroplast-Dependent and Independent Leaf Development
Source: Int J Mol Sci. 2022 Aug 31;23(17):9887. doi: 10.3390/ijms23179887 (PMC9456376; doi:10.3390/ijms23179887)

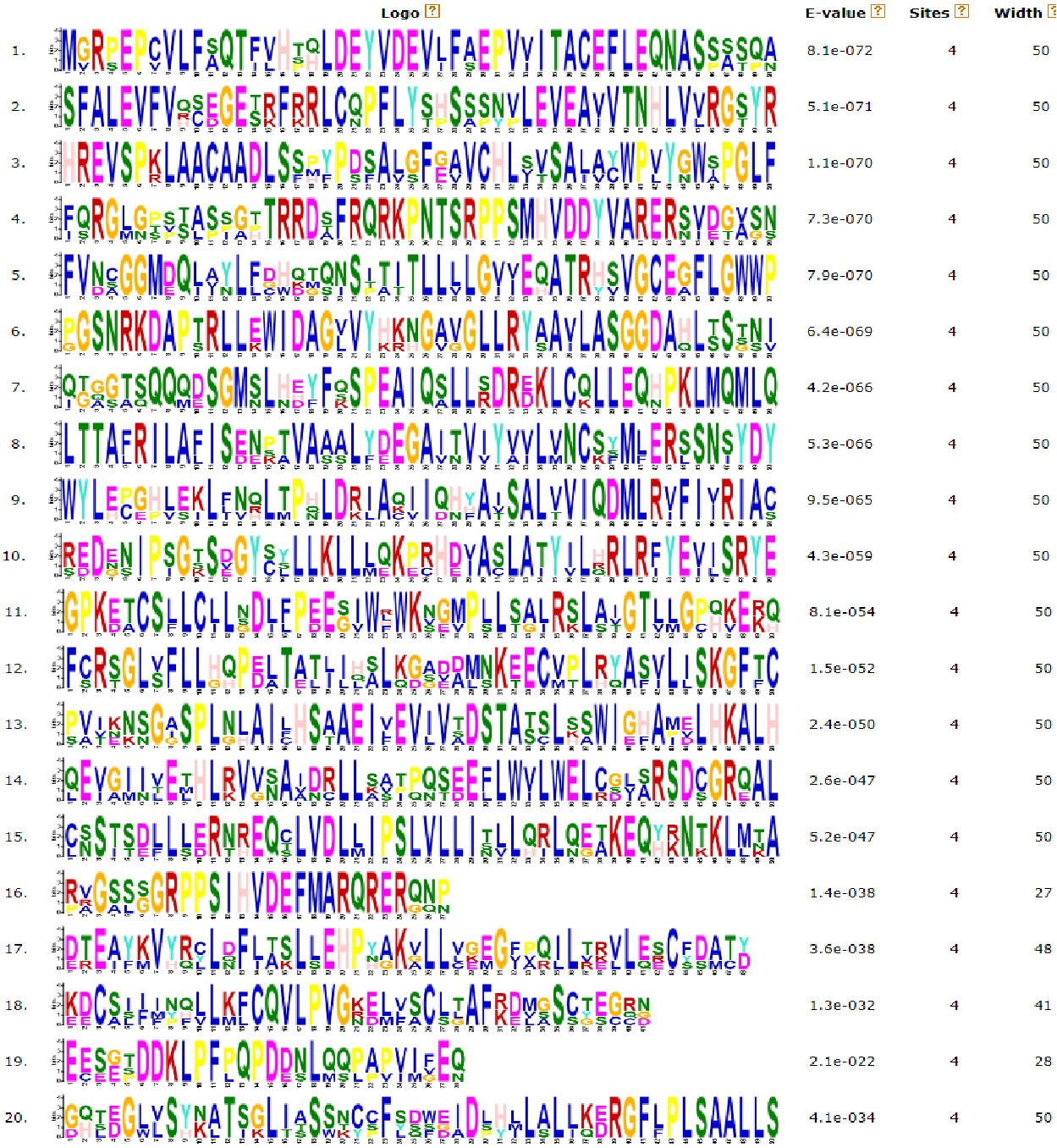

Supplement: Supplementary file 1 [file ijms-23-09887-s001.zip › Figure S2.jpg]

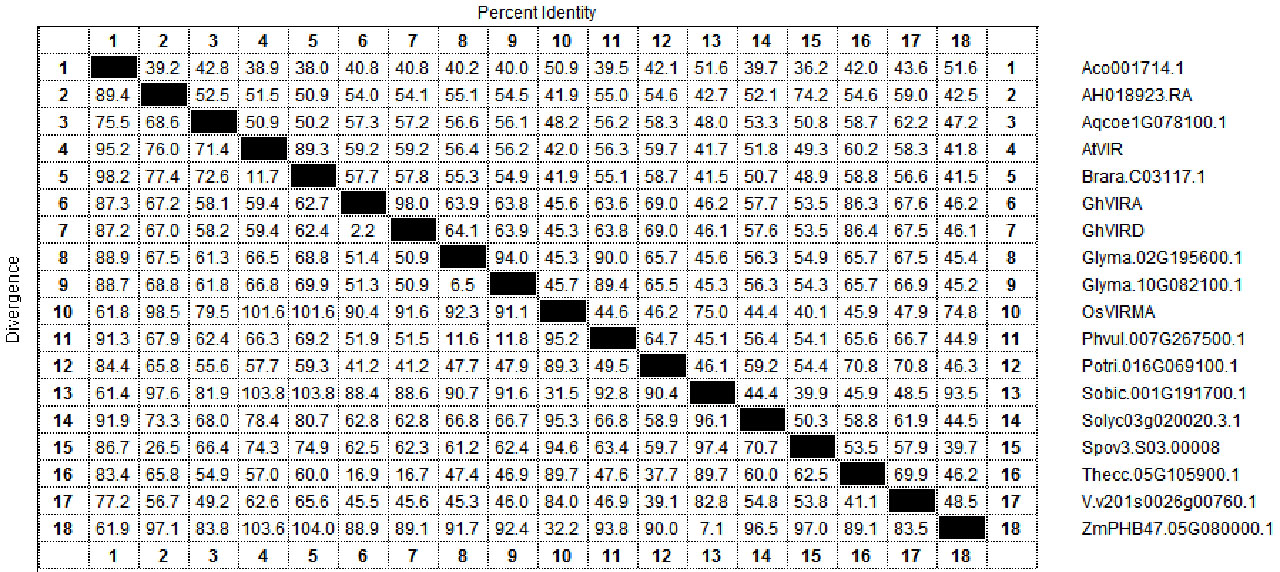

Supplement: Supplementary file 1 [file ijms-23-09887-s001.zip › Figure S3.jpg]

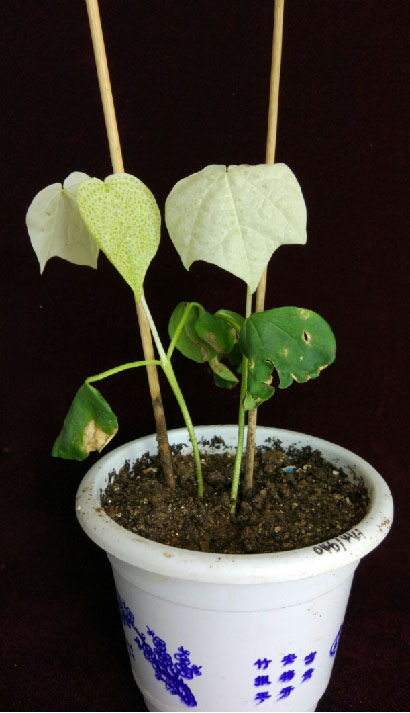

Supplement: Supplementary file 1 [file ijms-23-09887-s001.zip › Figure S4.jpg]

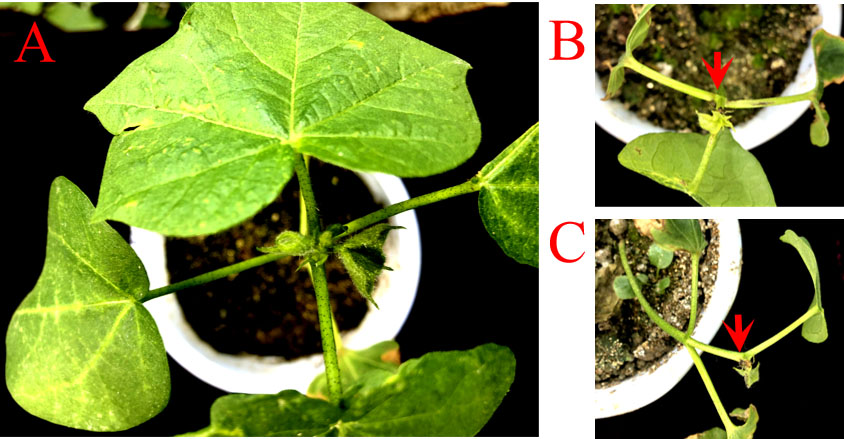

Supplement: Supplementary file 1 [file ijms-23-09887-s001.zip › Figure S5.jpg]
